# Promoting sustainable human mobility for income segregation mitigation

**DOI:** 10.1016/j.patter.2025.101477

**Published:** 2026-03-02

**Authors:** Yong Chen, Chenlei Liao, Zeen Cai, Wanru Wang, Yingji Xia, Xiqun (Michael) Chen, Jianjun Wu, Ziyou Gao

**Affiliations:** 1 School of Information Technology and Artificial Intelligence, Zhejiang University of Finance and Economics, Hangzhou 310018, China; 2 Institute of Intelligent Transportation Systems, College of Civil Engineering and Architecture, Zhejiang University, Hangzhou 310058, China; 3 School of Management, Zhejiang University of Finance and Economics, Hangzhou 310018, China; 4 School of Economics and Management, Dalian University of Technology, Dalian 116024, China; 5 School of Systems Science, Beijing Jiaotong University, Beijing 100044, China

**Keywords:** human mobility, urban sustainability, inequality, travel behavior, income segregation, air pollution

## Abstract

Unraveling urban income segregation fosters social cohesion, urban sustainability, and equitable access to public resources and opportunities for all socioeconomic groups. Here, we show that locations with different segregation levels exhibit biased collective mobility patterns, tending to visit locations with lower segregation levels, which escalate with city size and infrastructure accessibility, and cannot be explained solely by distance and population. Using 1.4 million data points on human mobility, socioeconomic factors, and environmental pollution from 16,093 census tracts in 10 large US cities, we introduce the segregation visitation index to quantify this tendency and develop a human mobility model incorporating segregation constraints and a transfer ensemble optimization component, providing a structural interpretation for the discovered biased mobility. Our results reveal the intricate interplays among urban income segregation, mobility, and environmental exposure, emphasizing the importance of accounting for location-specific mobility differences in developing sustainable income segregation mitigation strategies.

## Highlights

• Reveal biased travel patterns from highly segregated to less segregated areas
• Propose a segregation visitation index to quantify biased human mobility
• Develop a segregation-constrained mobility model capturing structural flow dynamics
• Reveal interplays among urban income segregation, mobility, and pollution exposure

## The bigger picture

Urban income segregation arises from uneven access to amenities and opportunities, shaping not only where people live but also how they move through the city. Yet, most research stops at measuring segregation rather than examining how it influences daily mobility, which is central to social interaction, service access, and environmental exposure. Here, we combined large-scale mobility, socioeconomic, and environmental data from major US cities and developed a framework that moves beyond static spatial metrics. We categorized neighborhoods by segregation level, quantified their mobility tendencies through a simple segregation visitation index, and constructed a segregation-constrained mobility model that embeds structural limitations into travel prediction. Together, these tools allow us to isolate behavioral differences that distance, population, and urban scale alone cannot explain.

Our analyses reveal a consistent and previously overlooked pattern: residents from highly segregated neighborhoods systematically travel toward less segregated areas, reflecting gaps in access to essential services and becoming more pronounced in larger, more urbanized cities. These mobility patterns affect daily experiences—for example, by increasing air pollution exposure—demonstrating that segregation shapes inequality not only through where people live but also through how they move. By integrating segregation, mobility, and environmental conditions, our framework provides actionable insights for urban planning, including targeted improvements to public transit, facility distribution, and neighborhood amenities. Understanding these mobility-driven disparities can help cities reduce inequality, enhance community well-being, and foster inclusive, resilient, and sustainable urban development over time.

## Teaser

Despite extensive studies that have addressed the quantification of income segregation, its impact on human mobility remains unclear. This study introduces a segregation visitation index and a mobility prediction model to reveal biased travel patterns, showing how segregation and mobility reinforce inequalities and guide more inclusive, sustainable urban planning.

## Introduction

Urban inequality is a complex and widespread social phenomenon, with income,^1^ health,^2^ and employment^3^ inequalities being its primary manifestations. Due to the uneven distribution of social services and opportunities within large cities, various income groups experience unequal access to social resources. Wealthier groups typically have better access to healthcare services and educational resources and have more transportation options.^4^ These disparities lead to severe income segregation, visible in the concentration and separation of activity spaces for distinct social groups within the city.^5^ Income segregation, in turn, exacerbates urban inequality even further. Therefore, as the central force undermining urban social cohesion, it is essential to understand the income segregation induced by factors such as income, land use, air quality, and the built environment.

Over the past few decades, substantial efforts have been made to quantify income segregation. Due to the challenge of accurately characterizing travelers’ physical exposure, researchers generally quantify segregation by approximating the social interactions of various social groups within an area (e.g., neighborhoods and census tracts).^6^^,^^7^ For example, residential income segregation is measured by the degree to which different social groups are segregated within a place of residence.^8^^,^^9^ Nevertheless, most social interactions occur outside residential areas,^10^^,^^11^^,^^12^^,^^13^ where people engage in recreation or work. As mobility data, such as mobile phone signaling data and location-based service data, have become increasingly available, research on quantifying segregation has evolved from focusing on residential income segregation^6^^,^^7^^,^^8^ to encompassing more dynamic segregation^10^^,^^11^^,^^12^^,^^13^^,^^14^^,^^15^ experienced in human travels. This type of segregation is no longer solely induced by a city’s geographical boundaries; it influences where people go, what services they access, and who they encounter. Therefore, analyzing human travel behavior is a crucial means of understanding the dynamics of income segregation in large cities.

Understanding human travel behaviors has long been a hotspot in transportation research, urban studies, geography, and economics.^16^ Previous research has unveiled universal patterns and laws of human travel using diverse transportation data.^17^^,^^18^^,^^19^^,^^20^ Both individual-level (e.g., the exploration and preferential return [EPR] model^21^ and container model^22^) and population-level (e.g., the gravity model [GM]^23^ and radiation model^24^) models have been developed to reproduce and depict these patterns. Empirical studies have shown correlations between human travel behavior and socioeconomic dynamics,^25^ indicating that individuals often engage in activities in places aligned with their socioeconomic categories and interact with individuals of similar socioeconomic backgrounds. Further, some studies have measured the degree of segregation experienced in various locations based on travel data,^6^^,^^12^^,^^26^ comparing the travel patterns of different income groups to illustrate how mobility shapes income segregation.^15^^,^^26^ Conversely, income segregation constrains local collective mobility patterns, which in turn encode internal drivers of segregation. For example, people in locations with different segregation levels exhibit different complementary needs for services due to unequal access to urban facilities. However, prior research has primarily focused on describing the organizational structure of urban mobility^27^^,^^28^ or quantifying the degree of spatial segregation across locations,^12^^,^^26^^,^^29^ without delving into how people living in differently segregated areas actually move and behave within the urban system. In particular, it remains unclear whether and how travel scales or collective mobility structures differ across segregation levels and what consequences such disparities entail. These variations are not merely statistical—they may translate into tangible sustainability challenges, such as longer travel distances and heightened mobility burdens for disadvantaged groups, and increased exposure to air pollution. Therefore, gaining a more thorough understanding of segregation-constrained collective mobility patterns can aid in analyzing the intrinsic factors that shape income segregation and facilitate tailored sustainable measures to reduce regional disparities in resource allocation.

To bridge these gaps, we propose a general framework to elucidate how different degrees of income segregation constrain human travel behaviors between locations within large cities. First, we couple mobility data and socioeconomic data from 10 large US cities to calculate income segregation values for all locations and employ a parameter-free method to categorize segregation levels. Second, we analyze the scaling laws of human mobility across locations with different segregation levels and introduce the segregation visitation index (SVI) to explore and quantify the tendency of collective human flows in these locations under segregation constraints. The SVI allows us to establish connections with urban indicators, including city size, infrastructure accessibility, and pollutant emissions. Third, by examining the predictability and transferability of human mobility in locations with different segregation levels, we develop a segregation-constrained human mobility (SCHM) model, which embeds a transfer ensemble algorithm to estimate mobility flows between locations. The proposed model can capture the scaling laws of human mobility at individual and population levels while depicting segregation-constrained visitation patterns. Our model is general and outperforms baseline models across 10 large cities. Our study offers a perspective on exploring the interplay among income segregation, travel behaviors, and other complex variables, providing important implications for sustainable urban management, policy guidance, and mitigating urban inequality.

## Results

### Income segregation level

**Figure 1:**
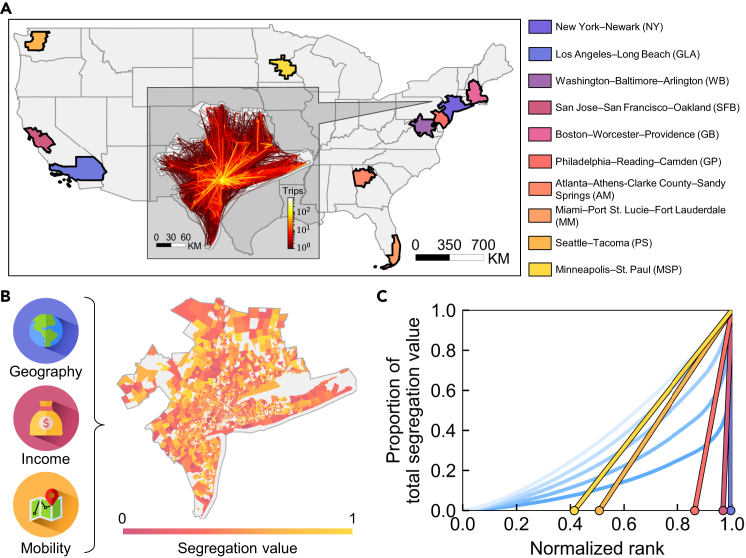
Categorization of income segregation levels (A) Geographical distribution of 10 large cities (combined statistical area [CSA]). The inset illustration represents the human flow network of all individuals within the New York-Newark CSA observed in the dataset. Line color indicates the number of trips between locations. Brighter colors indicate greater mobility flow, and vice versa. (B) Distribution of income segregation values experienced in all locations within the New York-Newark CSA. The color of each census tract indicates its corresponding segregation value. Income data are used to classify socioeconomic levels, and mobility data are used to determine the composition of income groups visiting each location. (C) Division of income segregation levels based on the Lorenz curve. The horizontal axis represents the normalized ranking of each location in ascending order of segregation value. The vertical axis represents the proportion of the cumulative segregation value to the total segregation value. A parameter-free method^27^^,^^31^ (see methods for details) is adopted to determine categorization thresholds for segregation levels adaptively.

To uncover collective mobility patterns among locations with different levels of income segregation, we use 7.1 million anonymized user check-in mobility data (see methods for details) to quantify income segregation across locations (i.e., census tracts) within 10 large cities (i.e., combined statistical areas [CSAs]) in the US (Figure 1A). To ensure both data completeness and representativeness, 10 CSAs with extensive mobility data coverage and long-term socioeconomic statistics were selected. These 10 CSAs have different populations and city sizes and encompass a wide range of historical and spatial contexts—ranging from early industrial and post-industrial centers with rich historical legacies (e.g., Philadelphia and Boston) to global innovation and financial hubs (e.g., New York and San Francisco) and rapidly expanding metropolitan regions undergoing economic diversification and demographic transformation (e.g., Atlanta and Minneapolis). This selection captures the heterogeneity of urban evolution, economic structures, and spatial organization across the US. It is worth noting that our study is applicable to cities with larger or smaller sizes. These 10 CSAs include New York-Newark (NY), Los Angeles-Long Beach (GLA), Washington-Baltimore-Arlington (WB), San Jose-San Francisco-Oakland (SFB), Boston-Worcester-Providence (GB), Philadelphia-Reading-Camden (GP), Atlanta-Athens-Clarke County-Sandy Springs (AM), Miami-Port St. Lucie-Fort Lauderdale (MM), Seattle-Tacoma (PS), and Minneapolis-St. Paul (MSP). Following established approaches,^30^ we infer each individual’s home location by examining the types of locations they checked in and identifying the most frequently visited locations between 9:00 p.m. and 6:00 a.m. We employ the per-capita income at that location as a proxy for the individual’s socioeconomic status (see methods for details). Meanwhile, all individuals within each CSA are divided into four quantiles. Based on the mobility data of all individuals, we quantify the degree of income segregation in each location by measuring the income diversity of its visitors^12^ (see Note S1 for details). The segregation value ranges from 0 (no segregation) to 1 (complete segregation), indicating whether a place is frequented by individuals from a single or diverse set of income groups. As an illustration, Figure 1B shows the segregation distribution within the NY CSA. Meanwhile, as shown in Figure S8, the selected 10 cities exhibit distinct degrees of income segregation: while regions within the NY and SFB CSAs show relatively high segregation values, most areas in the GLA, WB, GP, and AM CSAs display lower segregation values. This variability enables the capture of collective mobility patterns across contrasting segregation contexts, ensuring that our findings and the proposed model can be applied to both highly segregated and relatively integrated urban settings. Unlike residential segregation, the activity-based income segregation shows more continuous spatial variation and less pronounced block clustering.^8^ Our results show that the calculated segregation value distribution is robust to different quantile definitions, income group selections, and segregation metrics (see Notes S2 and S3 and Tables S2–S4).

Further, in the NY CSA, Spearman correlations between the segregation value of each location and both mobility flow and travel degree are −0.31 (*p* ≪ 0.05) and −0.39 (*p* ≪ 0.05), respectively (see Figure S9). This indicates that highly segregated locations tend to attract fewer visitors and exhibit lower travel diversity—that is, individuals visit a smaller variety of destinations. Similar robust statistical results are observed across other CSAs (see Table S7 for details). Interestingly, the segregation value of a location is only weakly correlated with its average local income (e.g., Pearson correlation coefficient of 0.35, *p* ≪ 0.05, in the NY CSA), suggesting that segregation arises from more intricate behavioral and spatial dynamics rather than income alone.

To further categorize locations by their segregation level, we adapt a parameter-free method based on the Lorenz curve^27^^,^^31^ (see Figure 1C). Locations are first ranked in ascending order based on their segregation values to construct a cumulative distribution curve of income segregation (i.e., Lorenz curve) within each city. The steepness of various points on the curve depicts the concentration of segregation values. Consequently, the derivative at the point (1, 1) of the Lorenz curve is determined and extrapolated to the intersection with the *x* axis to establish the threshold for segregation-level categorization. Then, all locations with segregation values greater than this threshold are removed, and the Lorenz curve is regenerated. This process is repeated to categorize all locations into respective segregation levels (see methods for details).

### Differences in travel scales of collective human flows

**Figure 2:**
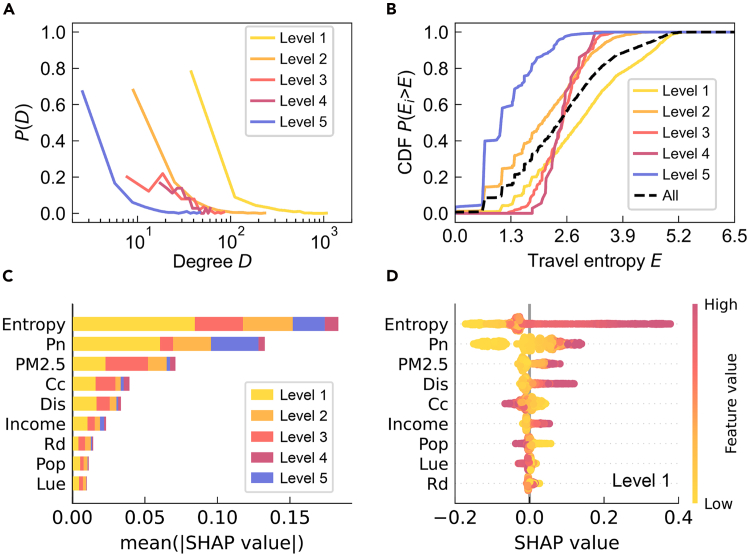
Heterogeneous collective mobility patterns across different segregation levels (A) Travel degree distribution of locations across different segregation levels. (B) Travel entropy distribution of locations across different segregation levels. The black dashed line represents the travel entropy distribution for all locations. (C) Comparison of the relative importance of features across segregation levels, with longer bars indicating greater significance. Pn, Pop, Rd, Dis, Cc, Lue, and PM2.5 denote the number of POI categories, population size, road density, travel distance, clustering coefficient, land-use entropy, and local annual PM2.5 emissions, respectively. (D) Distribution of SHAP values of all features within a single segregation level. Each scatter point represents a location, with color indicating the feature value. Points positioned to the right of the *x* axis signify a positive impact on model output, and vice versa.

To examine how collective mobility patterns vary across groups in locations with different segregation levels, we construct sub-networks of the citywide human flow network based on the segregation level of departure locations. For example, sub-network 1 includes all trips originating from locations with segregation level 1. For each sub-network, we measure the distributions of travel degree *D* and travel entropy *E*, which quantify the number and diversity of destinations reached from a given location, respectively (as shown in Figures 2A and 2B; see Note S3 and Figures S10 and S11 for details). Our analysis reveals clear differences in travel scales across segregation levels. Locations with lower segregation (e.g., level 1) exhibit broader and more diversified travel behaviors, reflected in higher values of travel degree and entropy. In contrast, highly segregated locations are associated with narrower and more localized mobility patterns. This trend is consistent across other mobility indicators, such as average travel distance and travel clustering coefficient (Cc) (see Note S5 and Figures S12 and S13 for details). Average travel distance reflects the spatial extent of movement, while Cc captures the transitivity of origin-based flows, indicating how tightly clustered individuals’ travel patterns are. Interestingly, locations with moderate segregation levels (e.g., levels 2 and 3) exhibit mixed mobility characteristics, acting as bridges that connect high- and low-income groups. This finding aligns with previous empirical studies^32^ and suggests that promoting inclusive land use and mixed-purpose development in such transitional areas may enhance social integration and reduce long-distance commuting.

Furthermore, we use the above mobility measures along with additional urban indicators as input to construct a segregation level classification model based on extreme gradient boosting^33^ (XGBoost) and evaluate the impact of each variable using Shapley additive explanations^34^ (SHAP) (see Note S6 for details). XGBoost identifies the most informative predictors of segregation level, while SHAP quantifies each feature’s marginal contribution to classification outcomes. Together, these analyses reveal which urban mobility and spatial characteristics most strongly influence segregation patterns, offering data-driven insights for designing policies that foster mobility equity and sustainable urban development. Figure 2C shows the relative importance of different variables across segregation levels, while Figure 2D illustrates their SHAP values at level 1 (see Figure S14 for further details). Among them, the number of points of interest (POI) categories (Pn) and land-use entropy (Lue) signify the service diversity of a location, road density (Rd) reflects the transportation convenience around a location, and the total annual emissions of particulate matter (PM2.5) measures the air quality of a location. We find that the relative impact of different variables varies among segregation levels. Specifically, at level 5, travel is predominantly influenced by Pn and has a lesser dependence on Rd. Locations (e.g., industrial parks) with higher segregation levels exhibit reduced service diversity and are typically frequented by minority groups addressing specific needs, potentially showing less concern for accessibility. Conversely, at level 1, a more convenient transportation infrastructure has a greater impact, encouraging a broader range of travel activities. Here, higher Rd supports greater travel variety, emphasizing the importance of investing in multimodal and sustainable transport networks to maintain inclusive accessibility. Notably, these low-segregation zones are also associated with higher PM2.5 emissions, indicating that greater mobility diversity may come at the cost of increased environmental exposure—a trade-off relevant for urban sustainability planning.

### SVI

**Figure 3:**
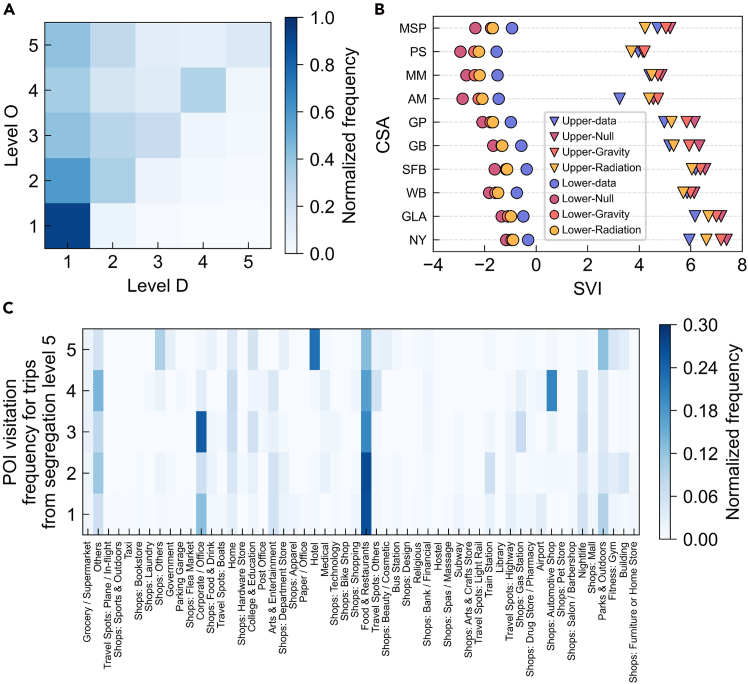
Segregation-constrained collective human flow patterns (A) Segregation-constrained visitation matrix for the New York-Newark CSA. Color indicates the visitation frequency between each segregation level, normalized based on the total number of trips from the origin level. (B) Comparison of observed SVI with three reference models across 10 large cities. “Upper” and “lower” represent downward and upward visitation tendencies, respectively. “Null,” “gravity,” and “radiation” represent the mobility flows generated based on a null model, a gravity model, and a radiation model, respectively (see Note S7 for details). The vertical axis displays the abbreviations of the 10 CSAs (see Table S1 for more information). (C) Distribution of POI visitation frequencies for trips from locations with segregation level 5 to other locations in the New York-Newark CSA. Color indicates the visit frequencies between each segregation level, normalized based on the total number of trips to the destination level.

To examine how income segregation shapes collective mobility tendencies, we construct a segregation-constrained visitation matrix SV that quantifies trip flows between locations of different segregation levels (see Figure 3A). Each matrix element represents the normalized probability of traveling from an origin level to a destination level. Our findings reveal that, aside from visiting locations with similar segregation levels, groups in locations at each segregation level consistently exhibit a downward visitation tendency (see Figure S15 for details), i.e., they prefer to visit locations with a lower segregation level. As shown in Figure 3C, we visualize the frequency distribution of POI visits from locations with segregation level 5 to locations with other segregation levels. Locations with low segregation levels better cater to needs such as dining and office spaces (e.g., “food & restaurant” and “corporate/office” POIs) and offer a wider range of leisure and entertainment services (e.g., “nightlife” and “arts & entertainment” POIs). Comparisons of POI diversity between origin and destination locations (see Figure S18) confirm that destinations generally offer a broader range of facilities and services. This suggests a potential association between higher segregation levels and reduced diversity or adequacy of local facilities. Groups in high-segregation locations tend to travel to destinations with more diverse services, potentially reflecting compensatory behavior in response to limited local service availability. These results highlight the value of expanding service diversity and improving local amenities in high-segregation locations, which could reduce long-distance travel and promote more equitable access to urban opportunities. It should be noted that these downward visits do not necessarily indicate higher consumption intensity; rather, they often involve routine activities such as commuting to workplaces, schools, or major public facilities. In contrast, due to the relative completeness of infrastructure in low-segregation locations, groups there show a lower tendency to travel to high-segregation locations, except when visiting essential places such as workplaces, schools, and transportation stations (see Figures S16 and S17 for details). Meanwhile, individuals from medium-segregation locations display more diversified mobility patterns, consistent with their mixed land-use context. Such locations function as bridging spaces, simultaneously receiving inflows from higher-segregation locations and sending flows toward more integrated locations. Leveraging this bridging role—through targeted planning to enhance transport accessibility and service complementarity—could strengthen spatial and social integration across the urban landscape. It is essential to emphasize that we do not consider income segregation a direct indicator of facility availability. Instead, we examine the distribution of visits to different types of POIs across segregation levels to explore the potential relationship between income segregation and access to urban services.

We further propose the SVI (see methods for details) to quantify the directional tendency of collective movements across different segregation levels. Let the discrete segregation levels be indexed by 1, …, *L*, where larger indices correspond to higher segregation levels. Based on the segregation-constrained visitation matrix SV, the SVI is defined as

(Equation 1) $$\text{SVI}=\sum_{l_{i},l_{j}=1}^{L}si_{l_{i},l_{j}}\cdot \left(l_{i}-l_{j}\right)$$

where $si_{l_{i},l_{j}}$ denotes the normalized visitation frequency with which a group starting from segregation level *l*_*i*_ visits segregation level *l*_*j*_. (*l*_*i*_ − *l*_*j*_) represents the span between two segregation levels. The value of SVI reflects the overall directionality of visitation tendencies within the city. If the diagonal elements of matrix SV are one, the SVI value equals zero (i.e., individuals of all levels exclusively visit locations of their level). A positive SVI indicates a downward visitation tendency (i.e., a preference for visiting locations with a lower segregation level than one’s own), while a negative value indicates an upward visitation tendency. When only one direction is considered (i.e., *l*_*i*_ < *l*_*j*_ or *l*_*i*_ > *l*_*j*_), we obtain the lower SVI and the upper SVI, respectively. To assess whether the observed tendencies arise from biased visits among groups in locations with different segregation levels and whether they can be replicated by common spatial interaction patterns, we construct three reference models (i.e., null-based, gravity-based, and radiation-based models) with distinct movement constraints for comparative analysis. The null-based model assumes random travel with equal probabilities, the gravity-based model incorporates distance decay, and the radiation-based model integrates intervening opportunities^24^ (e.g., population size) (see Note S7 for details). Figure 3B presents a comparative analysis between empirical SVI values and those generated by the reference models, with the SVI decomposed into upper and lower components whose sum represents the total SVI. We further compare observed SVI values against those from the null-based model to reveal deviations between empirical human flows, physically modeled flows, and random mobility patterns (see Figure S19). The results show that none of the reference models successfully replicates the empirical SVI patterns. All models tend to overestimate upward visitation tendencies and either over- or underestimate downward tendencies to varying degrees. Although the gravity and radiation models consider distance and population effects, they inherently assume symmetric movement potentials between locations and therefore fail to capture the socio-spatial asymmetry embedded in real mobility. In contrast, empirical mobility exhibits persistent directional biases across segregation levels—biases cannot be explained by spatial frictions alone. These results underscore that collective mobility patterns are not merely a function of distance or opportunity structures but are systematically shaped by income-based socio-spatial inequalities. Furthermore, by incorporating alternative segregation metrics and performing uncertainty estimation analyses (see Note S4, Tables S5 and S6, and Figures S20–S22), we validate the robustness of the proposed SVI formulation.

**Figure 4:**
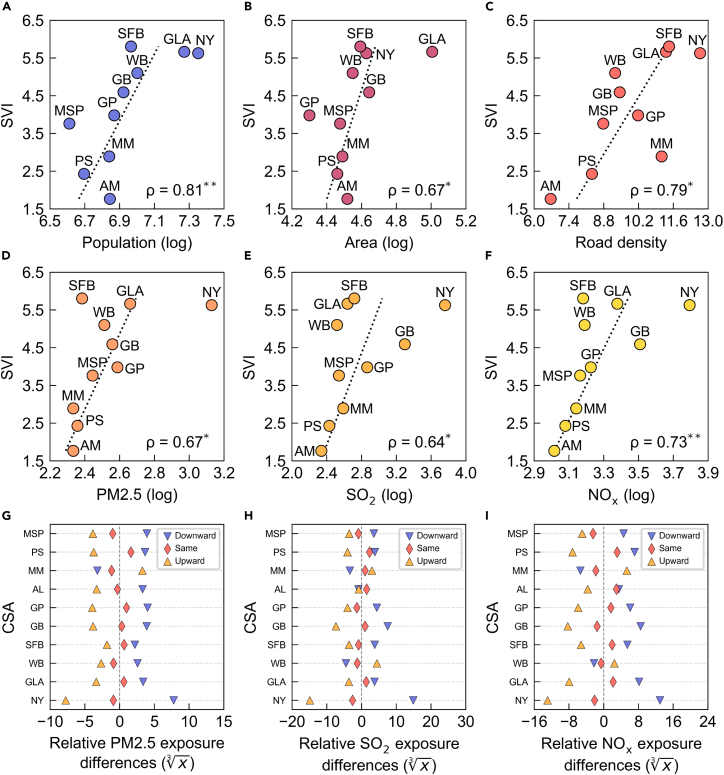
Correlation between SVI and urban indicators Correlation between SVI and urban indicators: (A) population, (B) area, (C) road density, (D) PM2.5 emissions, (E) SO_2_ emissions, and (F) NO_x_ emissions. Pollutant emissions are the total annual emissions in tons. *ρ* denotes the Spearman correlation coefficient. ∗∗*p* < 0.01 and ∗∗*p* < 0.05, respectively. (G–I) Average relative differences in pollutant emissions between travel origins and destinations across 10 large cities. “Upward,” “downward,” and “same” represent trips to locations with higher, lower, and the same segregation levels compared to the origin, respectively.

Furthermore, human mobility is shaped by the spatial distributions of urban infrastructure and population. To explore potential associations between segregation visitation patterns and broader urban characteristics, we examine correlations between the SVI and several urban indicators, including population size, urban area, average Rd, and pollutant emissions (i.e., PM2.5, SO_2_, and NO_x_). These indicators are closely linked to decision-making for sustainable urban governance and public health management.^35^^,^^36^ As shown in Figures 4A–4F, the SVI exhibits significant positive correlations with these indicators (*p* < 0.05). Please refer to Figures S20–S22 for more results on uncertainty estimation analysis. For example, larger cities with more extensive transportation infrastructures (e.g., New York and GLA) tend to facilitate greater downward visitation tendencies—individuals from highly segregated locations more frequently visit less segregated destinations. In such cities, well-developed transit systems expand the accessible spatial range, thereby increasing the likelihood of cross-segregation travel toward lower segregation levels. In contrast, smaller cities (e.g., PS and AM) typically feature more balanced land and housing accessibility and lower economic concentration, leading to weaker downward visitation tendencies. This implies that, in large cities, improving connectivity and transportation accessibility within highly segregated areas can promote equitable mobility, while in smaller cities, enhancing local service diversity and targeted amenities may be more effective than expanding transit infrastructure. Figures 4D–4F further illustrate the relationship between the SVI and the average annual total air pollutant emissions (tons per year) at travel destinations within each CSA. We find that higher SVI values are associated with increased exposure to air pollution during travel, as destinations of downward visits tend to exhibit higher pollutant emissions. Industrialized and densely urbanized cities (e.g., NY and GB) show particularly elevated exposure risks, reflecting the compounding effects of long-distance mobility constrained by segregation. To further assess emission disparities across segregation levels, we calculate the relative differences in pollutant emissions for trips to destinations with the same (same), higher (upward), and lower (downward) segregation levels as the origin. As demonstrated in Figures 4G–4I, downward trips consistently exhibit greater pollution burdens at destinations, whereas upward trips experience notably lower emission levels; same-level trips show minimal differences. These findings indicate that reducing cross-segregation travel—for instance, by increasing local amenities in high-segregation neighborhoods or promoting low-emission mobility modes—can yield dual benefits: enhancing mobility equity and advancing environmental sustainability.

### SCHM prediction

**Figure 5:**
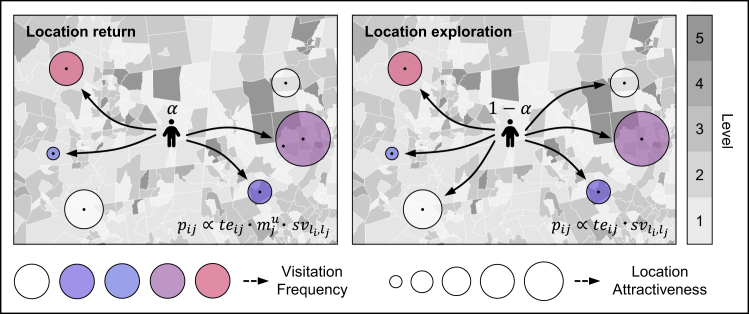
Illustration of a segregation-constrained human mobility model The individual selects returning to a location with probability *α* or engaging in location exploration with probability 1 − *α*. When individual *u* is located at location *i*, he/she will consider memory constraints (i.e., historical visit frequency $m_{j}^{u}$; depicted as the circle’s color) and location attractiveness constraints (*te*_*j*_; depicted as the circle’s size) to select a location to visit. Meanwhile, the location selection of the individual will be influenced by the income segregation of the current location, characterized by the visitation frequency ($sv_{l_{i},l_{j}}$; depicted as color shades on the map) between locations with different segregation levels. The location exploration performs similar steps but does not consider memory constraints.

To accurately reproduce and characterize mobility patterns under income segregation constraints, we develop an SCHM model (see Figure 5). The model comprises location exploration and return by jointly considering location attractiveness, individual memory, and segregation constraints. For each individual *u*, he/she decides with probability *α* whether to engage in travel activities subject to memory constraints. When the individual opts to return to a previously visited location, the probability of moving from location *i* to location *j* is $p_{ij}\propto {te}_{j}\cdot m_{j}^{u}\cdot sv_{l_{i},l_{j}}$. Location attractiveness *te*_*j*_ reflects the popularity or pull of a destination—the higher its attractiveness, the greater the likelihood of visitation. Historical memory $m_{j}^{u}$ encodes individual preferences for previously visited locations. In this study, $m_{j}^{u}$ is defined by the historical visitation frequency of individual *u* to each location. *te*_*j*_ is characterized by the sum of the travel probabilities from other locations to the target location. In this study, we introduce a transfer ensemble model to enhance the estimation accuracy of the travel distribution between locations. In addition, an individual’s travel choice is also constrained by the visitation probability between different segregation levels (i.e., $sv_{l_{i},l_{j}}$), ensuring that travel decisions follow empirically observed directional biases between segregation levels (e.g., downward or upward visits). The probability $sv_{l_{i},l_{j}}$ is derived from the segregation-constrained visitation matrix SV. In the absence of historical memory constraints, travel choice simplifies to $p_{ij}\propto {te}_{j}\cdot sv_{l_{i},l_{j}}$, indicating that mobility is jointly determined by destination attractiveness and segregation-level visitation tendencies.

**Figure 6:**
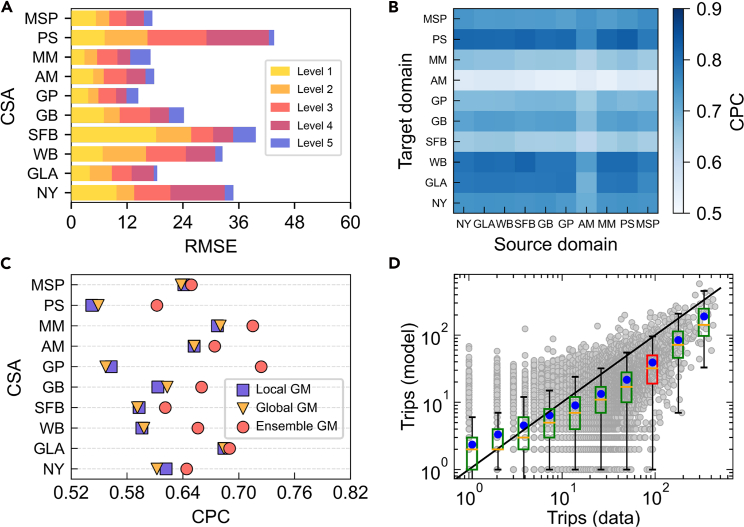
Predictability and transferability analysis of collective human flows in different cities (A) Comparison of the predictability of collective human flows across segregation levels, with longer bars indicating greater RMSE. (B) Transfer prediction performance for collective human flows at locations with the same segregation level (level 5) between different large cities. (C) Performance comparison of the proposed transfer ensemble model and baseline models. (D) Paired comparisons of predicted and real trips within the New York-Newark CSA. Gray points indicate observed and predicted location pairs. The boxplot illustrates the distribution of predicted trips across various ranges of observed trip counts. The green-shaded box indicates that the diagonal line *y* = *x* falls within the 5th and 95th percentiles, and red otherwise. Blue points represent the average predicted trip counts across different bins.

For our proposed transfer ensemble model, we first observe variations in the predictability of collective human flows across segregation levels using origin-constrained GMs^16^ (Figure 6A). The root-mean-square error (RMSE) decreases as the segregation level increases, indicating that mobility patterns in highly segregated locations (e.g., level 5) are more regular and predictable, whereas those in low-segregation areas are more diverse and dynamic. This heterogeneity poses challenges for constructing a single, citywide GM (global GM) capable of accurately capturing mobility across all segregation levels. In contrast, separate models trained for each level (local GM) better fit level-specific dynamics but fail to generalize across the urban system (see Note S8 for model details). To examine whether mobility patterns are transferable between cities, we estimate a GM using collective human flow data from locations within a large city at a certain segregation level (i.e., source domain). We then transfer this model to predict human flows for locations in other large cities with the same segregation levels (i.e., target domain) (see methods for details). As shown in Figure 6B, transfer predictions for level-5 flows achieve performance comparable to models trained directly on target-city data, suggesting that human mobility behaviors exhibit cross-city regularities within the same segregation strata (see Figure S23 for details). “CPC” denotes the common part of commuters,^16^ measuring the similarity between predicted and actual flows. Building on this observation, we introduce a transfer ensemble model (i.e., ensemble GM) to enhance estimation accuracy beyond traditional gravity-based approaches. Specifically, we train multiple origin-constrained GMs using data from several cities sharing the same segregation level. These models generate a set of transfer-based predictions for the target city, which are aggregated into a feature matrix and fed into an XGBoost meta-learner to produce the final human flow estimation (see methods for details). As shown in Figures 6C and 6D, the proposed ensemble GM consistently outperforms both the local GM and the global GM across major cities. In the NY CSA, for example, the estimated trips closely match observed flows, with a Pearson correlation of 0.75 (*p* ≪ 0.001) (see Figure S24 for more examples). The transferability of mobility patterns suggests that lessons learned from one city can be applied to others with similar segregation profiles, potentially guiding scalable strategies for improving equitable access to services and mitigating associated environmental impacts, including pollution exposure.

**Figure 7:**
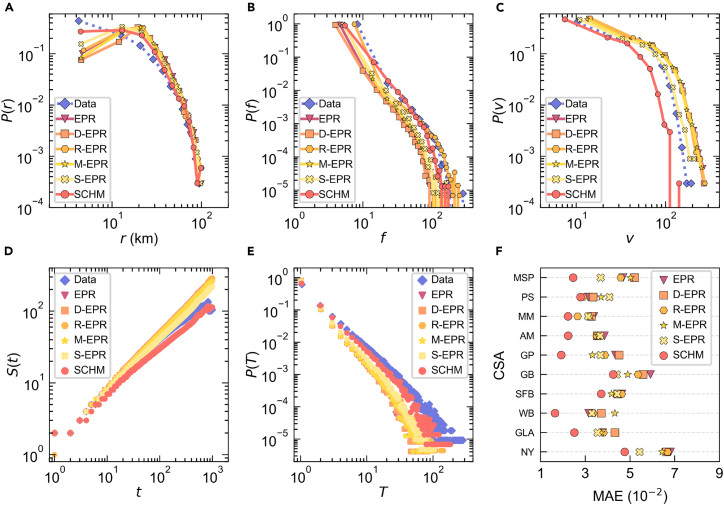
Human mobility prediction performance (A) Distribution of radius of gyration in the New York-Newark CSA. (B) Frequency distribution of individuals visiting a location in the New York-Newark CSA. (C) Distribution of the number of locations visited by individuals in the New York-Newark CSA. (D) Distribution of the total number of locations visited within *t* trips in the New York-Newark CSA. (E) Comparison between predicted and actual distributions of the different number of trips *T* between two locations in the New York-Newark CSA. (F) Distribution of prediction errors in visitation probability between locations with different segregation levels for 10 large cities.

Finally, we utilize a subset of historical mobility data from 10 large cities to fit the SCHM model and generate synthetic travel trajectories for each (see methods for details). The travel sample encompasses 12,937 census tracts across 201 counties in the US. To evaluate the model’s accuracy, we compare the generated trajectories against empirical travel data across six key metrics (see Figure 7): the distribution of radius of gyration, the distribution of individuals’ location visitation frequency, the distribution of the number of locations visited by individuals, the total number of location visits within *t* trips, the number of trips *T* between locations, and the visitation frequency error between locations with different segregation levels. These indicators jointly assess model performance from individual, collective, and segregation perspectives. Please refer to Figures S25–S31 for more performance evaluations. For benchmarking, we introduce the EPR model^21^ and its four variant models, i.e., gravity-based EPR (D-EPR),^19^ recency-based EPR^37^ (R-EPR), memory-based EPR^38^ (M-EPR), and social EPR^12^ (see Note S9 for details), for performance comparison. All models were run under identical initial conditions and simulation settings, following consistent trajectory-generation procedures and parameter calibrations drawn from prior empirical studies.^19^^,^^21^^,^^37^^,^^38^

As shown in Figure 7, SCHM markedly outperforms all EPR-based baselines (see Table S8). The synthetic trajectories produced by SCHM closely replicate the empirical distributions of key mobility indicators. Compared with EPR-based models, which primarily encode memory- or distance-based returns and exploration strategies, SCHM integrates a transfer ensemble method that leverages cross-city, segregation-level-consistent mobility patterns, substantially enhancing the generalizability and predictive accuracy of gravity-based flow estimation. These design choices enable SCHM to more accurately reproduce empirical scaling laws at individual and population levels while significantly reducing prediction errors in human flows between locations. For instance, at the individual level, the prediction results of SCHM are closer to the empirical distribution than those of baseline models in describing scaling laws, such as the radius of gyration, location visitation frequency, and the number of locations visited. Additionally, SCHM accurately captures the scaling laws of the algebraic increase in the total number of location visits. At the population level (Figure 7E), SCHM achieves superior accuracy in predicting aggregate trips between locations, closely matching the empirical algebraic decay and convergence patterns of inter-location flows. Moreover, SCHM yields the smallest visitation-frequency error between locations with different segregation levels for all large-city datasets (Figure 7F; see Table S9 and Figure S31 for details). Among the EPR-based models, S-EPR performs better by incorporating income-segregation constraints, yet it still falls short of SCHM’s performance. These results underscore SCHM’s ability to capture the structural heterogeneity of urban mobility by integrating segregation-level-aware visitation constraints—an essential aspect often overlooked by traditional mobility models (e.g., D-EPR and R-EPR). The approach yields more realistic trajectory generation and a more accurate depiction of cross-segregation-level flow dynamics. From a practical standpoint, SCHM offers a scalable and generalizable framework for quantifying how income segregation shapes the scale and direction of urban travel. By explicitly modeling segregation-specific behavioral regularities and their transferability across cities, SCHM enables urban planners and policymakers to diagnose mobility inequities and to simulate systemic impacts of interventions. For example, the model can evaluate how improving transit accessibility in high-segregation neighborhoods, redistributing essential services, or adjusting land-use configurations would alter cross-level mobility flows and overall network efficiency.

## Discussion

Urbanization is rapidly accelerating, making urban income segregation an increasingly prominent and pressing issue. This study provides a lens for disentangling the complex interplay between income segregation and human mobility patterns, moving beyond traditional location-level metrics.^11^^,^^12^ By categorizing urban locations into different segregation levels, we uncover pronounced heterogeneity in collective mobility patterns among locations experiencing various degrees of income segregation. In particular, locations with moderate segregation serve as crucial “bridging zones” that foster cross-group connectivity between high- and low-income groups. Moreover, we reveal that segregation levels are closely associated with disparities in local service provision, which, in turn, drive biased visitation patterns and reinforce unequal access to urban amenities. A consistent downward visitation trend—where groups from highly segregated areas travel to less segregated zones—is observed across major cities and becomes increasingly pronounced in larger, more infrastructure-intensive urban environments. Notably, this structural mobility bias also leads to disproportionate exposure to air pollution, highlighting a neglected dimension of environmental injustice.

To model these nuanced dynamics, we propose a SCHM model embedded with a transfer ensemble framework. This model not only replicates empirical scaling laws but also offers a robust structural account of how structural inequalities shape behavioral mobility decisions. The integration of segregation-aware constraints enables us to capture the cognitive trade-offs individuals make between accessibility, opportunity, and environmental exposure. From a behavioral perspective, residents in highly segregated locations tend to exhibit compensatory mobility strategies—traveling further to satisfy unmet service or social needs—whereas individuals in less segregated locations display higher spatial inertia due to localized service sufficiency. These behavioral adaptations provide a human-centered interpretation of how structural inequalities manifest in aggregate mobility flows.

Taken together, these findings highlight the importance of incorporating segregation effects into the modeling and planning of sustainable and equitable urban mobility systems. Beyond theoretical contributions, our work carries concrete societal and policy relevance. It directly informs the design of spatially targeted interventions that can alleviate inequality while promoting long-term sustainability. Empirically, we demonstrate that groups from highly segregated locations may compensate for deficiencies in essential services by visiting locations with lower segregation levels. This insight guides urban planners in prioritizing specific service facilities around locations with high segregation levels, such as large convenience supermarkets, outdoor recreational facilities, and public transportation stations. These actions not only reduce travel burdens and carbon emissions but also foster local economic vitality, social cohesion, and community well-being—key dimensions of sustainable and inclusive urban development.

Furthermore, our results reveal a trade-off between urban expansion and intra-city segregation intensity in large cities. In cities with higher urbanization, individuals exhibit a stronger preference for low-segregation destinations, suggesting an imbalance in urban facility distribution. This underscores the need for policymakers to align infrastructure investment and service allocation with equitable development objectives. At the same time, our findings underscore that compensatory long-distance travel may inadvertently increase environmental and health risks^39^ through greater air pollution exposure. Addressing this dual challenge requires integrative policy design—combining service accessibility improvement with targeted pollution mitigation. For example, implementing stricter emission controls in high-traffic bridging zones or expanding low-emission public transit routes can jointly reduce exposure disparities and enhance environmental justice.

Crucially, these policy insights are operationalizable through our SCHM framework. Our SCHM framework provides a computational platform that allows policymakers to simulate and evaluate the real-world impacts of intervention strategies—such as facility redistribution, zoning reforms, and emission controls—on both mobility behavior and social equity outcomes. By incorporating segregation-level-aware visitation constraints and a transfer-based ensemble learning mechanism, SCHM facilitates fine-grained “what-if” analyses, empowering urban planners to design strategies that are evidence based, equitable, and sustainability oriented. In this sense, our model functions as a decision-support tool that translates behavioral insights into operational policy guidance.

In contrast to existing location-based or individual-based segregation measures, this study focuses on uncovering variations in collective mobility patterns among groups in locations across different levels of segregation and provides actionable insights into mitigating income segregation and promoting sustainable urban development. Nevertheless, several limitations should be acknowledged. First, our analysis uses income-based segregation as a proxy for broader urban segregation, potentially overlooking other dimensions such as race, education, or occupation. While our framework is extensible, future work could benefit from a multidimensional segregation characterization. Second, our mobility analysis relies on check-in data, which may not fully represent contemporary urban mobility behaviors or demographic conditions. Nevertheless, the validity of our conclusions primarily rests on the structural and behavioral patterns uncovered rather than the specific temporal context of the data. The fundamental relationships identified—such as the compensatory visitation behaviors among groups in highly segregated areas and the bridging role of moderately segregated zones—reflect enduring spatial and socioeconomic dynamics that are likely relevant across periods. However, the generalizability of the empirical results to present-day urban contexts should be interpreted with caution, as the magnitude of segregation-mobility interactions may shift with evolving spatial configurations and socioeconomic conditions. To further enhance validity and generalizability, future research should recalibrate and validate the SCHM framework using more recent and diversified mobility datasets—such as mobile phone signaling or transit smart-card records—that better represent population heterogeneity and trip purposes. Moreover, longitudinal analyses could help assess the temporal persistence of the observed patterns and disentangle structural regularities from context-dependent effects. Third, socioeconomic attributes are estimated at the location level rather than the individual level due to privacy constraints. Future studies could improve granularity by integrating additional data sources, such as anonymized income, housing, or consumption data. Finally, although SCHM supports policy simulation under hypothetical scenarios, it assumes static segregation during simulations. Future extensions could integrate feedback mechanisms between mobility behaviors, segregation dynamics, and policy responses to better reflect the evolving nature of urban systems.

## Methods

### Ethics and inclusion statement

The research did not include local researchers. The roles and responsibilities were agreed upon among collaborators ahead of the research. This research did not include biological materials, animals, or human subjects, for whom ethical approval is required.

### Dataset

We leverage mobility data derived from user check-ins gathered on the Weeplaces^40^ website, which integrates location-based check-in information from various service platforms (e.g., Facebook and Gowalla). This dataset is a collated and anonymized dataset containing 7,164,507 travel records (i.e., check-ins) of 15,790 users spanning January 2010 to June 2011. Each record includes the anonymous user’s identifier, travel time, travel location (including location’s identifier, longitude, and latitude), and activity type. This study uses census tracts as the fundamental unit of travel analysis. The movement of an individual from one location to another is considered a trip (or a new location visit), regardless of whether the destination is their place of residence. The maximum interval between an activity being considered as a trip was 24 h (see Figure S1). For consecutive records in the same census tract, we retained only one trip; that is, intra-zonal trips were not considered. At the same time, we also removed individuals with fewer than 8 trips to reduce random bias. Based on the activity types of all users’ historical travel, we can count the number of POI categories in a census tract, which measures the service diversity of a location. Finally, we exclusively consider trips occurring within 10 large cities (i.e., CSAs), which cover 16,093 census tracts across 201 US counties (see Figure 1A; Table S1) and contain 1,453,936 trips for 10,524 individuals. The distribution of trips of all individuals is shown in Figure S2. The distribution of the number of visits to each census tract (including residents and non-residents) is shown in Figure S3. Since some places were visited by fewer people, we excluded census tracts that were visited fewer than 30 times to reduce the bias of the results. A CSA comprises multiple metropolitan areas where individuals often participate in economically, politically, and culturally relevant spatial interactions. We employ a standardized procedure^30^ to infer each individual’s home location by considering the location category of the individual’s check-ins and the most frequently visited location between 9:00 p.m. and 6:00 a.m. Further, we use the per-capita income of the census tract, where the home is situated as a proxy,^10^^,^^41^ for the income level of the target individual. We follow the established pipeline^26^ to verify that the inferred individual income levels within each CSA accurately reflect the population distribution (see Note S2 and Figure S4). Meanwhile, the income of all individuals within a large city is divided into four equal-sized quantile intervals. Each individual is classified into groups of different economic levels: low income, low-middle income, higher-middle income, and high income. All administrative boundary data and socioeconomic data (i.e., income and population) are obtained from the official website of the US Census Bureau.^42^ Moreover, urban indicator data related to the city, including urban area, road network density, and land-use data, are gathered from the OpenStreetMap^43^ database. Air pollutant emission data are collected from the global atmospheric research emission database (EDGAR).^44^ Meanwhile, Lue is calculated using the information entropy method.^45^ EDGAR provides a gridded annual total of pollutant emissions, which we project to each census tract based on the grid center coordinates (see Figures S5–S7).

### Segregation-level categorization

The Lorenz curve is widely utilized for calculating the Gini coefficient, which measures the equality of income distribution in countries and regions.^46^ Meanwhile, the slopes of the curve also contain valuable information. This study adapts the LouBar method^27^^,^^31^ to adaptively establish thresholds for categorizing different levels of income segregation.

We first rank the segregation values of all locations within a target city in ascending order and subsequently obtain the cumulative distribution curve (i.e., Lorenz curve) of the segregation values of all locations (Figure 1C). The curve’s horizontal axis represents the ranking of each location, and the vertical axis represents the proportion of total segregation value. If the probability of occurrence for all segregation values is uniformly distributed, the Lorenz curve will form a straight line from 0, 0 to 1, 1. Since the slopes of different points on the curve indicate the degree of aggregation for segregation values, points with a larger slope have a lower degree of aggregation. Consequently, we take the derivative at the point (1, 1) of the Lorenz curve and extrapolate to the intersection of the *x* axis to obtain the segregation threshold *T*_*L*_. We then remove the locations with segregation values greater than or equal to *T*_*L*_ from the curve, generate a new Lorenz curve (light blue curve in Figure 1C), and so forth. Ultimately, all locations are categorized into different segregation levels. To facilitate the comparison of various large cities, we have categorized them into up to five segregation levels.

### Transfer ensemble model

Building on the predictability and transferability analysis of different segregation levels in collective human flows across various large cities, we propose a transfer ensemble model to enhance the accuracy of human flow estimation between various locations within large cities. To illustrate, considering the estimation of collective human flows of all locations with the segregation level equal to level 1 in the NY CSA, we utilize travel data (labeled as D_1_–D_10_) from locations with the same segregation level in 10 large cities to estimate 10 GMs, respectively. Meanwhile, we partition the dataset into a training set and a test set with a ratio of 8:2. Subsequently, we employ the estimated 10 GMs to perform transfer predictions on the training dataset of the NY CSA (D_1_), resulting in 10 sets of transfer prediction results for D_1_. For each data point, we stack 10 prediction results to create a new dataset, denoted as D_new_. Finally, we use XGBoost^33^ as the meta-estimator to establish a stacking-based ensemble prediction model and utilize D_new_ for training and estimating the travel distribution of the NY CSA. Leveraging transfer prediction and ensemble techniques at the same segregation level across large cities, the proposed model significantly enhances the accuracy of collective human flow estimation (see Figures 5C and 5D).

### Travel trajectory generation

We model individuals’ travel transitions by incorporating constraints across three dimensions: location attractiveness, individual memory, and visitation frequency between different segregation levels. The simulation involves generating individual travel trajectories through the following operations.

(1) Initialize the travel status information. Assume the travel length of individual *u* in the actual observation dataset is *Tl*_*u*_. Utilize historical travel data consisting of $\frac{1}{3}{Tl}_{u}$ steps to initialize the historical memory vector m^*u*^ for the target individual *u*, representing the frequency of visits to each location. Similarly, calculate the segregation-constrained visitation matrix SV using one-third of the historical travel data of all individuals. Moreover, employ the proposed transfer ensemble model to estimate the travel distribution between various locations and obtain location attractiveness vectors te.
(2) Next, assuming that the individual is currently at location *i*, based on the proposed human mobility model, the individual chooses a location to explore or return to.
(3) Update the current location of individual *u*, memory information, and cross-segregation level visitation information.
(4) Repeat steps 2 and 3 to generate a travel trajectory with $\frac{2}{3}{Tl}_{u}$ travel steps.

## Resource availability

### Lead contact

Further information and requests for resources and reagents should be directed to and will be fulfilled by the lead contact, Xiqun (Michael) Chen (chenxiqun@zju.edu.cn).

### Materials availability

This study did not generate new unique materials.

### Data and code availability

The travel data and other data related to this study are available from Zenodo^47^ (https://doi.org/10.5281/zenodo.15314012). National administrative division and socioeconomic data of the US are publicly available at https://www.census.gov/data.html. Road network and regional land-use data are publicly available at https://www.openstreetmap.org. Air pollutant emission data are publicly available at https://edgar.jrc.ec.europa.eu/. The code used for data processing and analysis, segregation calculation, segregation-level categorization, travel pattern analysis, and mobility model building and training is available from Zenodo^48^ (https://doi.org/10.5281/zenodo.13742568). The accession number for the code and pre-computed data reported in this paper is Zenodo: https://doi.org/10.5281/zenodo.13742568.

## Acknowledgments

This research is financially supported by the 10.13039/501100001809National Natural Science Foundation of China (72288101, 72525009, 72431009, 72171210, and 72350710798) and the 10.13039/501100004731Zhejiang Provincial Natural Science Foundation of China (LZ23E080002, LQN26E080004).

## Author contributions

Y.C., Z.G., and X.(M.)C. proposed the question. Y.C., C.L., W.W., and Y.X. designed and conducted the experiments. Y.C., Z.C., and X.(M.)C. developed the algorithms. Y.C., J.W., Z.G., and X.(M.)C. wrote the paper.

## Declaration of interests

The authors declare no competing interests.

## Declaration of generative AI and AI-assisted technologies in the writing process

The authors declare that no generative AI tools were used in the writing of this manuscript or in the editing of any associated figures.

## Supplemental information

Document S1. Figures S1–S31, Tables S1–S9, and Notes S1–S10

Document S2. Article plus supplemental information

## References

[1] Ravallion M. (2014). Income inequality in the developing world. Science.

[2] Bor J., Cohen G.H., Galea S. (2017). Population health in an era of rising income inequality: USA, 1980–2015. Lancet.

[3] Calvo-Armengol A., Jackson M.O. (2004). The effects of social networks on employment and inequality. Am. Econ. Rev..

[4] Macedo M., Lotero L., Cardillo A., Menezes R., Barbosa H. (2022). Differences in the spatial landscape of urban mobility: Gender and socioeconomic perspectives. PLoS One.

[5] Gambetta D., Mauro G., Pappalardo L. (2023). Mobility constraints in segregation models. Sci. Rep..

[6] Wang Q., Phillips N.E., Small M.L., Sampson R.J. (2018). Urban mobility and neighborhood isolation in America's 50 largest cities. Proc. Natl. Acad. Sci. USA.

[7] Van Ham M., Uesugi M., Tammaru T., Manley D., Janssen H. (2020). Changing occupational structures and residential segregation in New York, London and Tokyo. Nat. Hum. Behav..

[8] Tammaru T., Marcińczak S., Aunap R., van Ham M., Janssen H. (2020). Relationship between income inequality and residential segregation of socioeconomic groups. Reg. Stud..

[9] Reardon S.F., Bischoff K. (2011). Income inequality and income segregation. Am. J. Sociol..

[10] Nilforoshan H., Looi W., Pierson E., Villanueva B., Fishman N., Chen Y., Sholar J., Redbird B., Grusky D., Leskovec J. (2023). Human mobility networks reveal increased segregation in large cities. Nature.

[11] Athey S., Ferguson B., Gentzkow M., Schmidt T. (2021). Estimating experienced racial segregation in US cities using large-scale GPS data. Proc. Natl. Acad. Sci. USA.

[12] Moro E., Calacci D., Dong X., Pentland A. (2021). Mobility patterns are associated with experienced income segregation in large US cities. Nat. Commun..

[13] Wang S., Zheng Y., Wang G., Yabe T., Moro E., Pentland A.‘. (2024). Infrequent activities predict economic outcomes in major American cities. Nat. Cities.

[14] Bonaccorsi G., Pierri F., Scotti F., Flori A., Manaresi F., Ceri S., Pammolli F. (2021). Socioeconomic differences and persistent segregation of Italian territories during COVID-19 pandemic. Sci. Rep..

[15] Li X., Huang X., Li D., Xu Y. (2022). Aggravated social segregation during the COVID-19 pandemic: Evidence from crowdsourced mobility data in twelve most populated US metropolitan areas. Sust. Cities Soc..

[16] Barbosa H., Barthelemy M., Ghoshal G., James C.R., Lenormand M., Louail T., Menezes R., Ramasco J.J., Simini F., Tomasini M. (2018). Human mobility: Models and applications. Phys. Rep..

[17] Brockmann D., Hufnagel L., Geisel T. (2006). The scaling laws of human travel. Nature.

[18] Gonzalez M.C., Hidalgo C.A., Barabasi A.L. (2008). Understanding individual human mobility patterns. Nature.

[19] Pappalardo L., Simini F., Rinzivillo S., Pedreschi D., Giannotti F., Barabási A.L. (2015). Returners and explorers dichotomy in human mobility. Nat. Commun..

[20] Schläpfer M., Dong L., O’Keeffe K., Santi P., Szell M., Salat H., Anklesaria S., Vazifeh M., Ratti C., West G.B. (2021). The universal visitation law of human mobility. Nature.

[21] Song C., Koren T., Wang P., Barabási A.L. (2010). Modelling the scaling properties of human mobility. Nat. Phys..

[22] Alessandretti L., Aslak U., Lehmann S. (2020). The scales of human mobility. Nature.

[23] Zipf G.K. (1946). The P_1_ P_2_/D hypothesis: On the intercity movement of persons. Am. Sociol. Rev..

[24] Simini F., González M.C., Maritan A., Barabási A.L. (2012). A universal model for mobility and migration patterns. Nature.

[25] Boterman W.R., Musterd S. (2016). Cocooning urban life: Exposure to diversity in neighbourhoods, workplaces and transport. Cities.

[26] Hilman R.M., Iñiguez G., Karsai M. (2022). Socioeconomic biases in urban mixing patterns of US metropolitan areas. EPJ Data Sci..

[27] Bassolas A., Barbosa-Filho H., Dickinson B., Dotiwalla X., Eastham P., Gallotti R., Ghoshal G., Gipson B., Hazarie S.A., Kautz H. (2019). Hierarchical organization of urban mobility and its connection with city livability. Nat. Commun..

[28] Xu Y., Belyi A., Santi P., Ratti C. (2019). Quantifying segregation in an integrated urban physical-social space. J. R. Soc. Interface.

[29] Xu F., Wang Q., Moro E., Chen L., Salazar Miranda A., González M.C., Tizzoni M., Song C., Ratti C., Bettencourt L. (2025). Using human mobility data to quantify experienced urban inequalities. Nat. Hum. Behav..

[30] McNeill G., Bright J., Hale S.A. (2017). Estimating local commuting patterns from geolocated Twitter data. EPJ Data Sci..

[31] Louail T., Lenormand M., Cantu Ros O.G., Picornell M., Herranz R., Frias-Martinez E., Ramasco J.J., Barthelemy M. (2014). From mobile phone data to the spatial structure of cities. Sci. Rep..

[32] Leo Y., Fleury E., Alvarez-Hamelin J.I., Sarraute C., Karsai M. (2016). Socioeconomic correlations and stratification in social-communication networks. J. R. Soc. Interface.

[33] Chen T., Guestrin C. (2016). Proceedings of the 22nd ACM SIGKDD International Conference on Knowledge Discovery and Data Mining, San Francisco.

[34] Lundberg S.M., Lee S.I., von Luxburg Ulrike, Guyon Isabelle (2017). Proc. 31st Conf. on Neural Inf. Process. Syst..

[35] Martilli A. (2014). An idealized study of city structure, urban climate, energy consumption, and air quality. Urban Clim..

[36] Stone B. (2008). Urban sprawl and air quality in large US cities. J. Environ. Manage..

[37] Barbosa H., de Lima-Neto F.B., Evsukoff A., Menezes R. (2015). The effect of recency to human mobility. EPJ Data Sci..

[38] Alessandretti L., Sapiezynski P., Sekara V., Lehmann S., Baronchelli A. (2018). Evidence for a conserved quantity in human mobility. Nat. Hum. Behav..

[39] Hayes R.B., Lim C., Zhang Y., Cromar K., Shao Y., Reynolds H.R., Silverman D.T., Jones R.R., Park Y., Jerrett M. (2020). PM2.5 air pollution and cause-specific cardiovascular disease mortality. Int. J. Epidemiol..

[40] Liu Y., Wei W., Sun A., Miao C. (2014). Proc. 23rd ACM Int. Conf. on Inf. Knowl. Manage..

[41] Abbiasov T., Heine C., Sabouri S., Salazar-Miranda A., Santi P., Glaeser E., Ratti C. (2024). The 15-minute city quantified using human mobility data. Nat. Hum. Behav..

[42] U.S. Census Bureau. American community survey. https://www.census.gov/data.html.

[43] OpenStreetMap contributors. OpenStreetMap. https://www.openstreetmap.org.

[44] Crippa M., Guizzardi D., Pagani F., Schiavina M., Melchiorri M., Pisoni E., Graziosi F., Muntean M., Maes J., Dijkstra L. (2024). Insights into the spatial distribution of global, national, and subnational greenhouse gas emissions in the Emissions Database for Global Atmospheric Research (EDGAR v8.0). Earth Syst. Sci. Data.

[45] Lei D., Chen X., Cheng L., Zhang L., Ukkusuri S.V., Witlox F. (2020). Inferring temporal motifs for travel pattern analysis using large scale smart card data. Transp. Res. Pt. C-Emerg. Technol..

[46] Blesch K., Hauser O.P., Jachimowicz J.M. (2022). Measuring inequality beyond the Gini coefficient may clarify conflicting findings. Nat. Hum. Behav..

[47] Chen, Y. (2025). Data for Promoting sustainable human mobility for income segregation mitigation. Zenodo. 10.5281/zenodo.15314012. PMC13100684 42028405

[48] Chen, Y. (2024). Segregation-constrained human mobility model. Zenodo. 10.5281/zenodo.13742568.

