# Supplementary material for: Promoting sustainable human mobility for income segregation mitigation
Source: Patterns (N Y). 2026 Mar 2;7(3):101477. doi: 10.1016/j.patter.2025.101477 (PMC13100684; doi:10.1016/j.patter.2025.101477)
Supplement: Document S1. Figures S1–S31, Tables S1–S9, and Notes S1–S10 [file mmc1.pdf]

Patterns, Volume *7*

Supplemental information

Promoting sustainable human mobility

for income segregation mitigation

Yong Chen, Chenlei Liao, Zeen Cai, Wanru Wang, Yingji Xia, Xiqun (Michael) Chen, Jianjun Wu, and Ziyou Gao

Supplemental Information

Supplemental figures

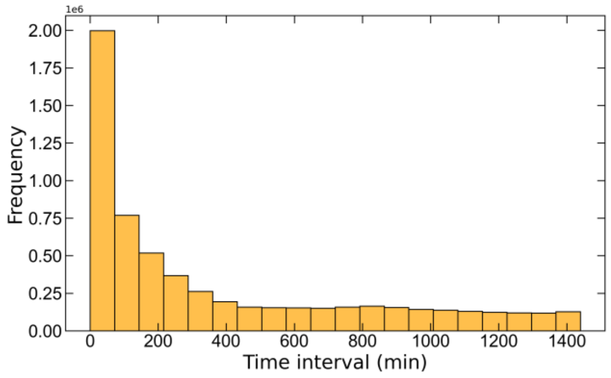

Figure S1. Time interval distribution between two consecutive trips for all individuals, with most individuals exhibiting time intervals within four hours between visits.

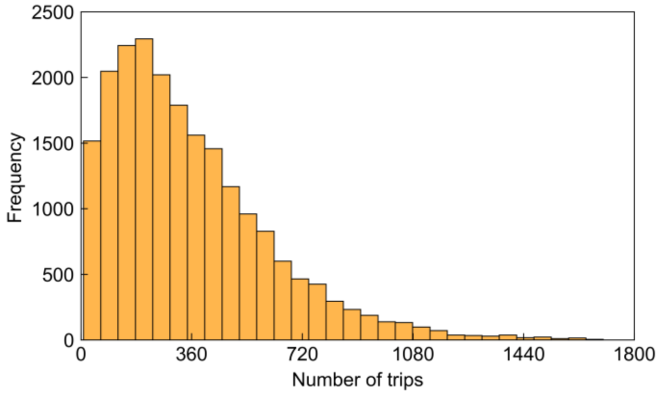

Figure S2. The distribution of trips of all individuals.

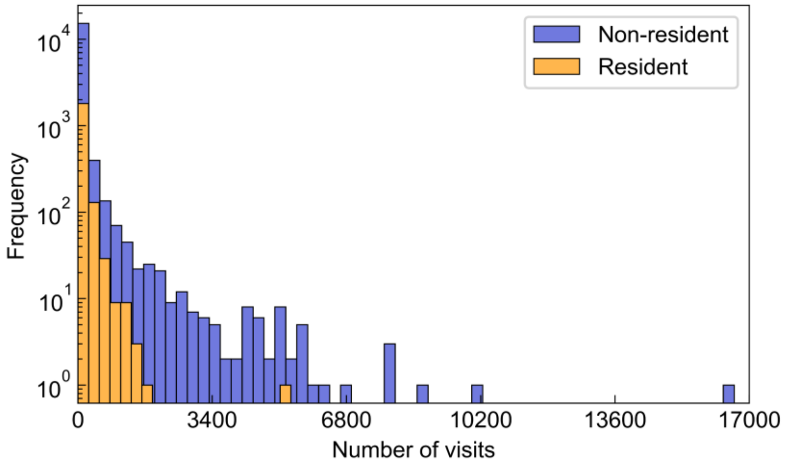

Figure S3. Distribution of visits to each census tract.

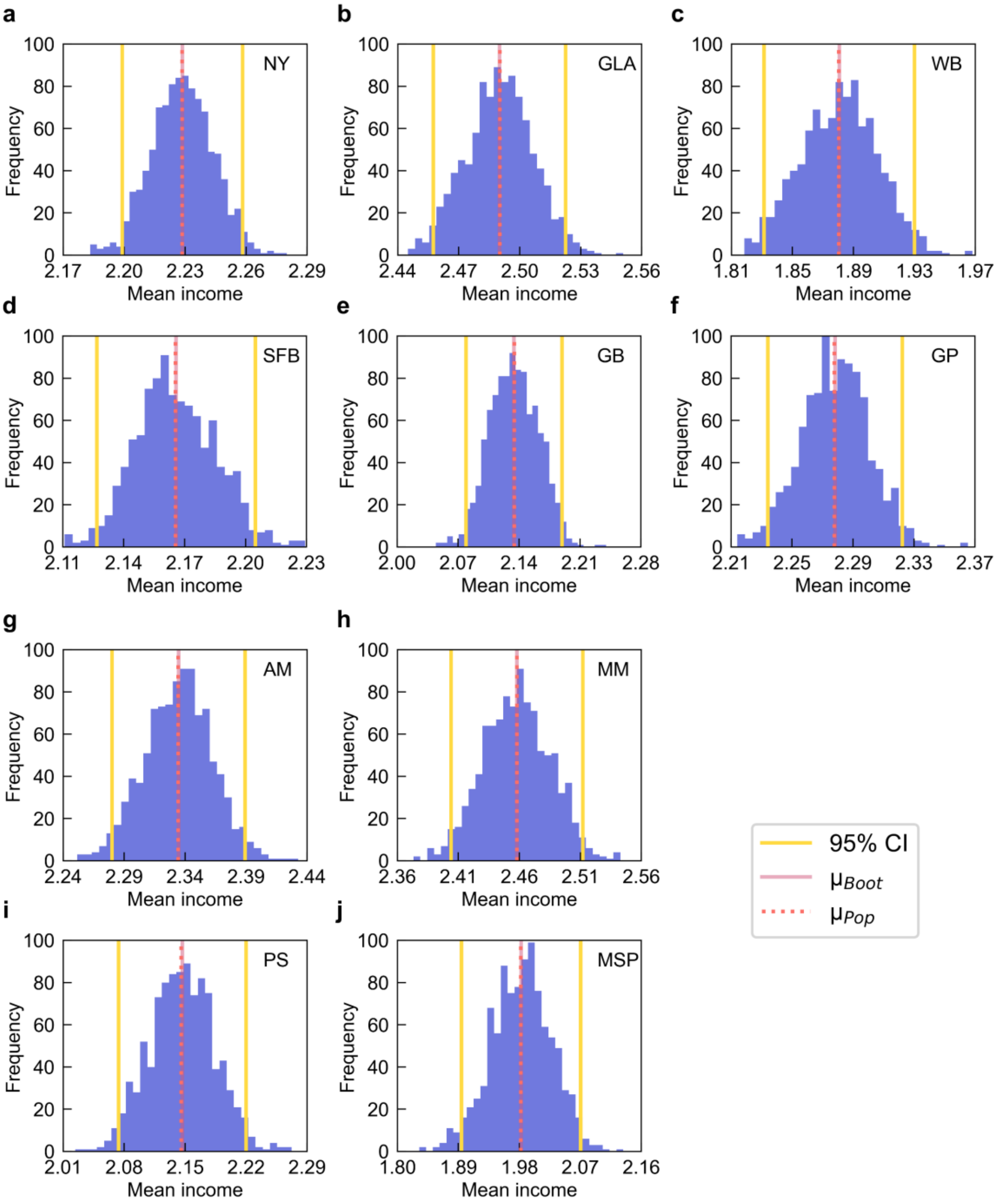

Figure S4. Bootstrapping results. The histogram represents the distribution of mean user income across the 1,000 resampled datasets obtained via bootstrapping. The yellow line denotes the 95% confidence interval for the mean income distribution. The pink line indicates the mean income across all sample sets. The red dashed line represents the mean income value of the actual user population.

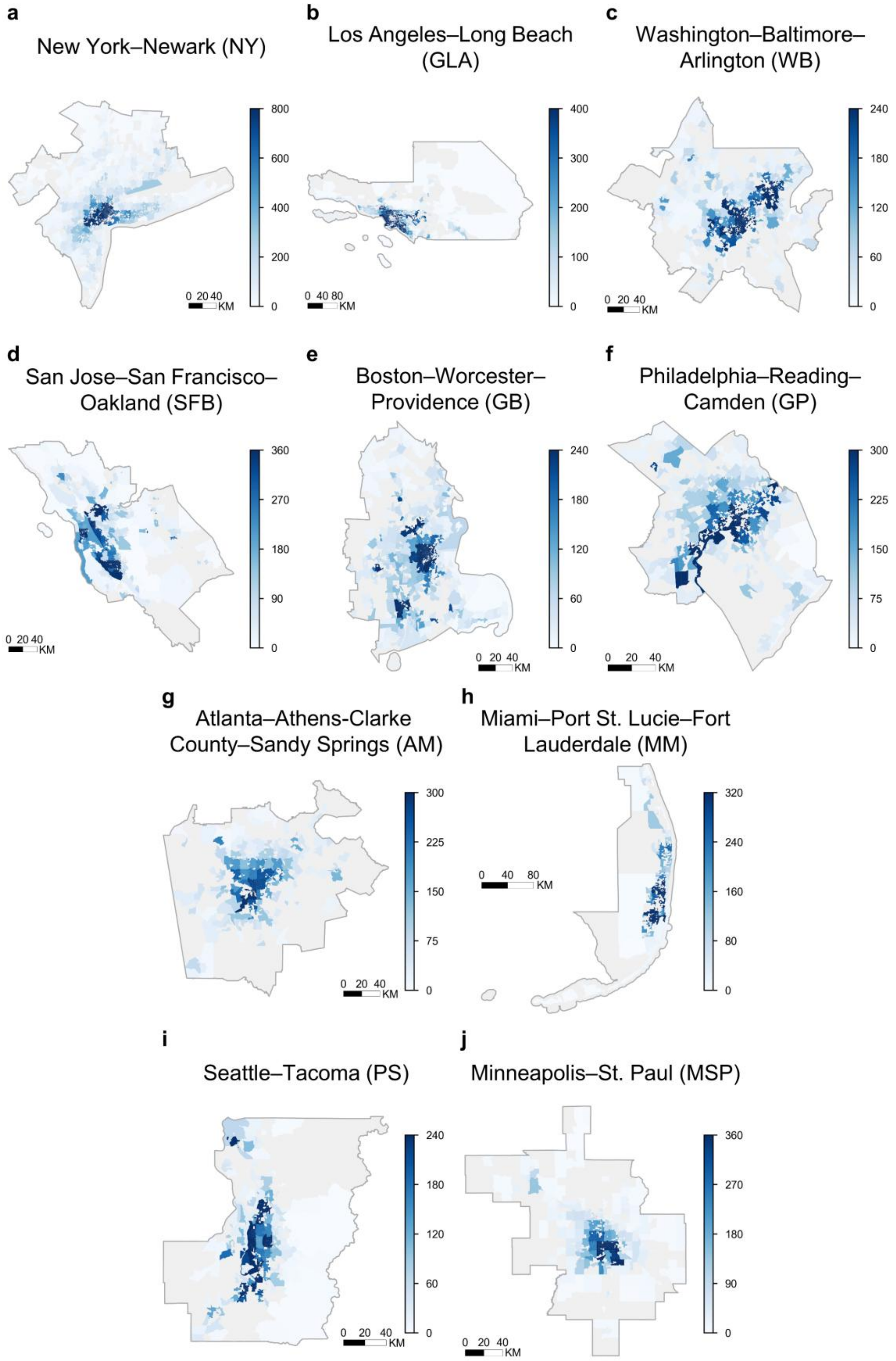

Figure S5. Distribution of total annual PM2.5 emissions for all locations across all large cities. The color of each census tract indicates its corresponding PM2.5 emissions in tonnes.

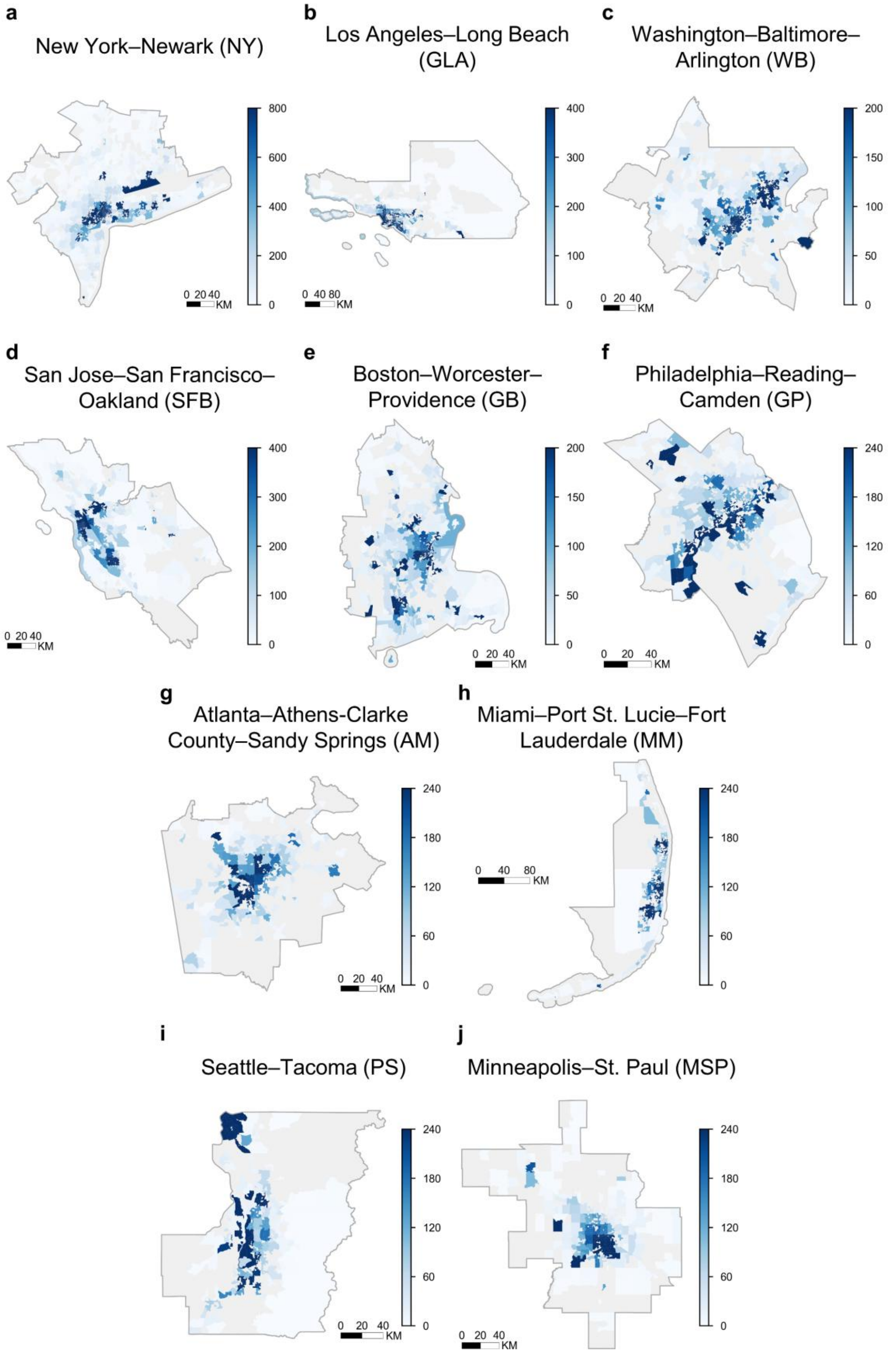

Figure S6. Distribution of total annual SO_2 emissions for all locations across all large cities. The color of each census tract indicates its corresponding SO_2 emissions in tonnes.

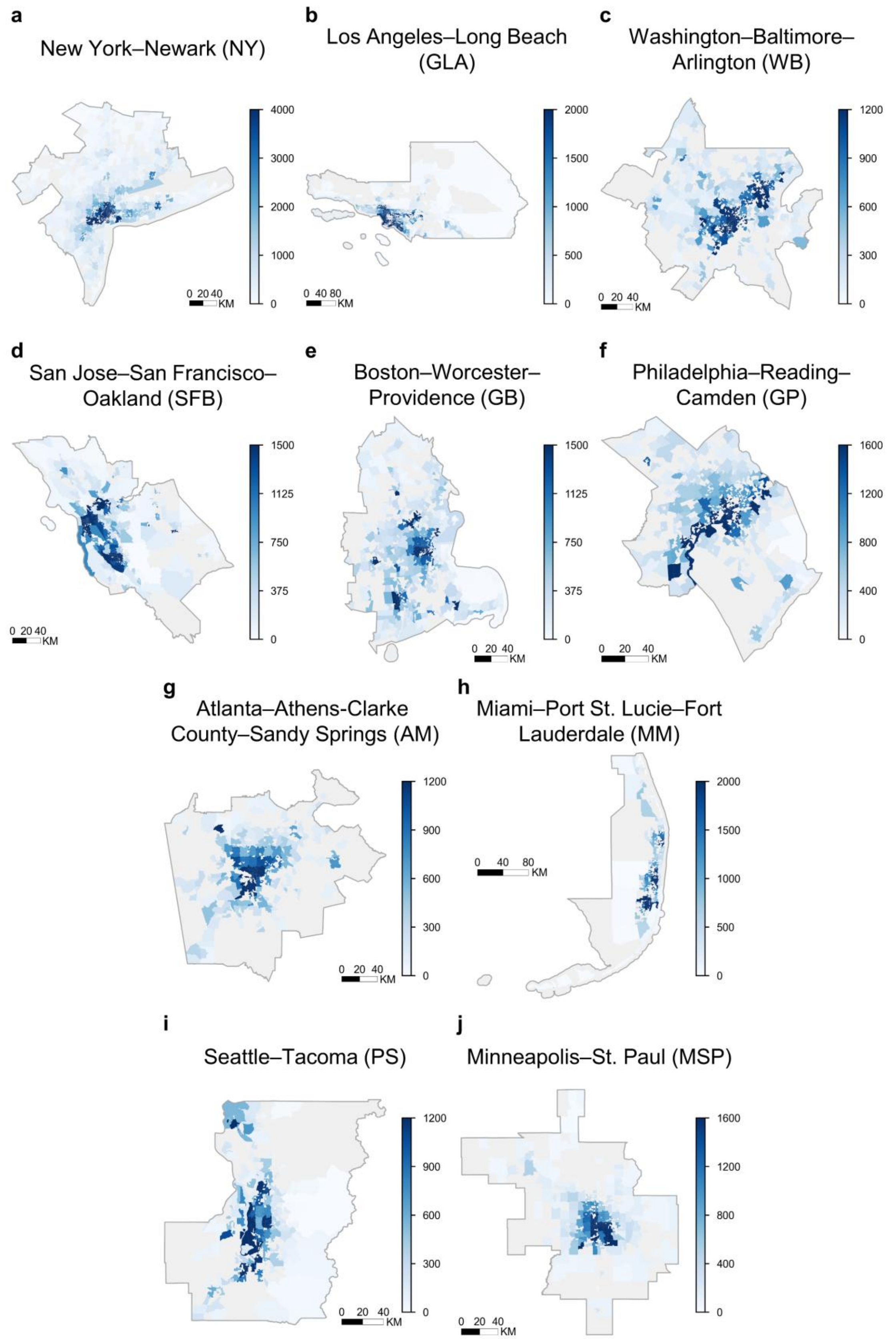

Figure S7. Distribution of total annual NO_x emissions for all locations across all large cities. The color of each census tract indicates its corresponding NO_x emissions in tonnes.

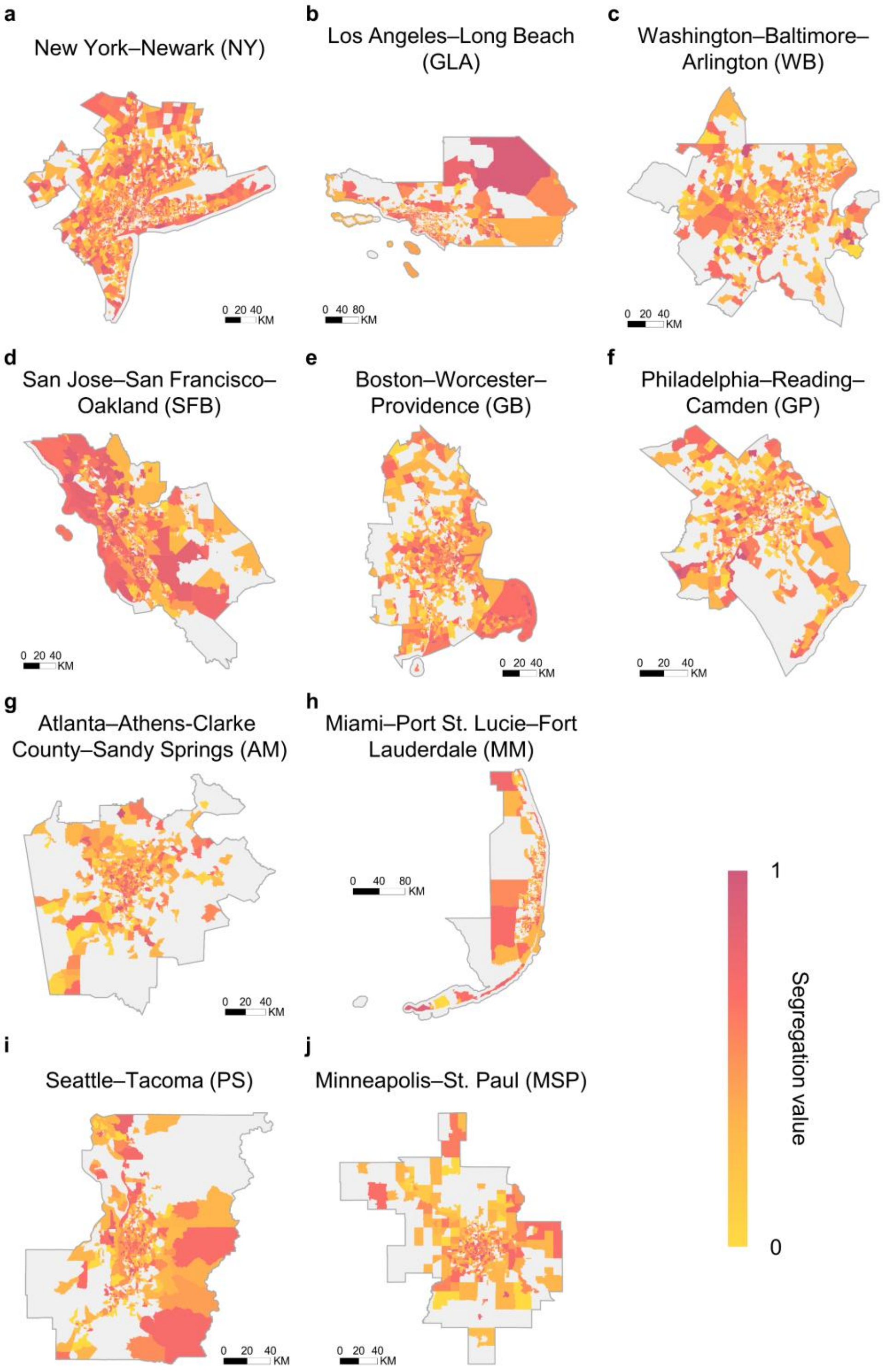

Figure S8. Distribution of income segregation values experienced in all locations across all large cities. The color of each census tract indicates its corresponding segregation value.

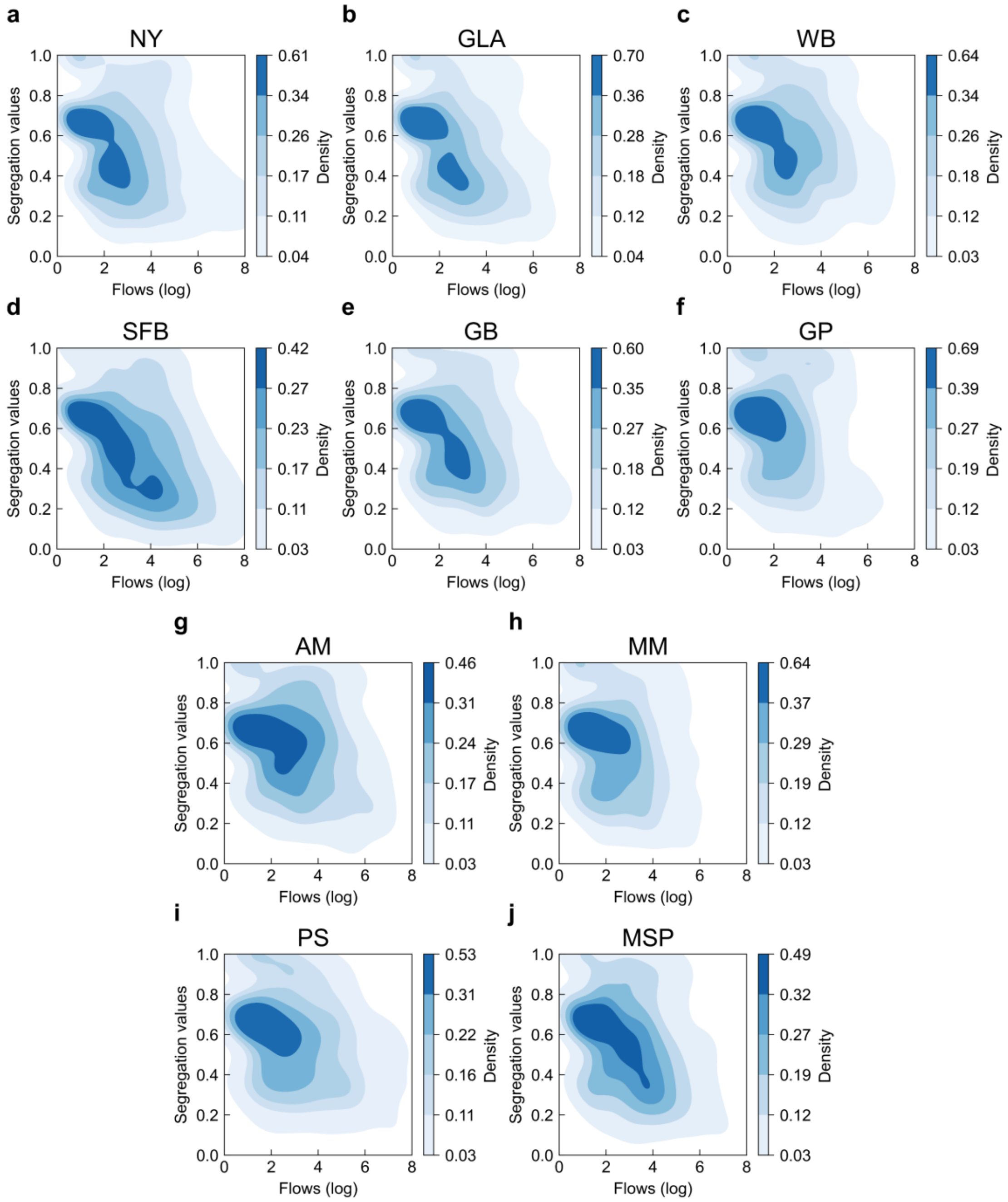

Figure S9. Correlation of mobility flows and segregation values for all census tracts within each city. The colors represent the density of the scattered data points.

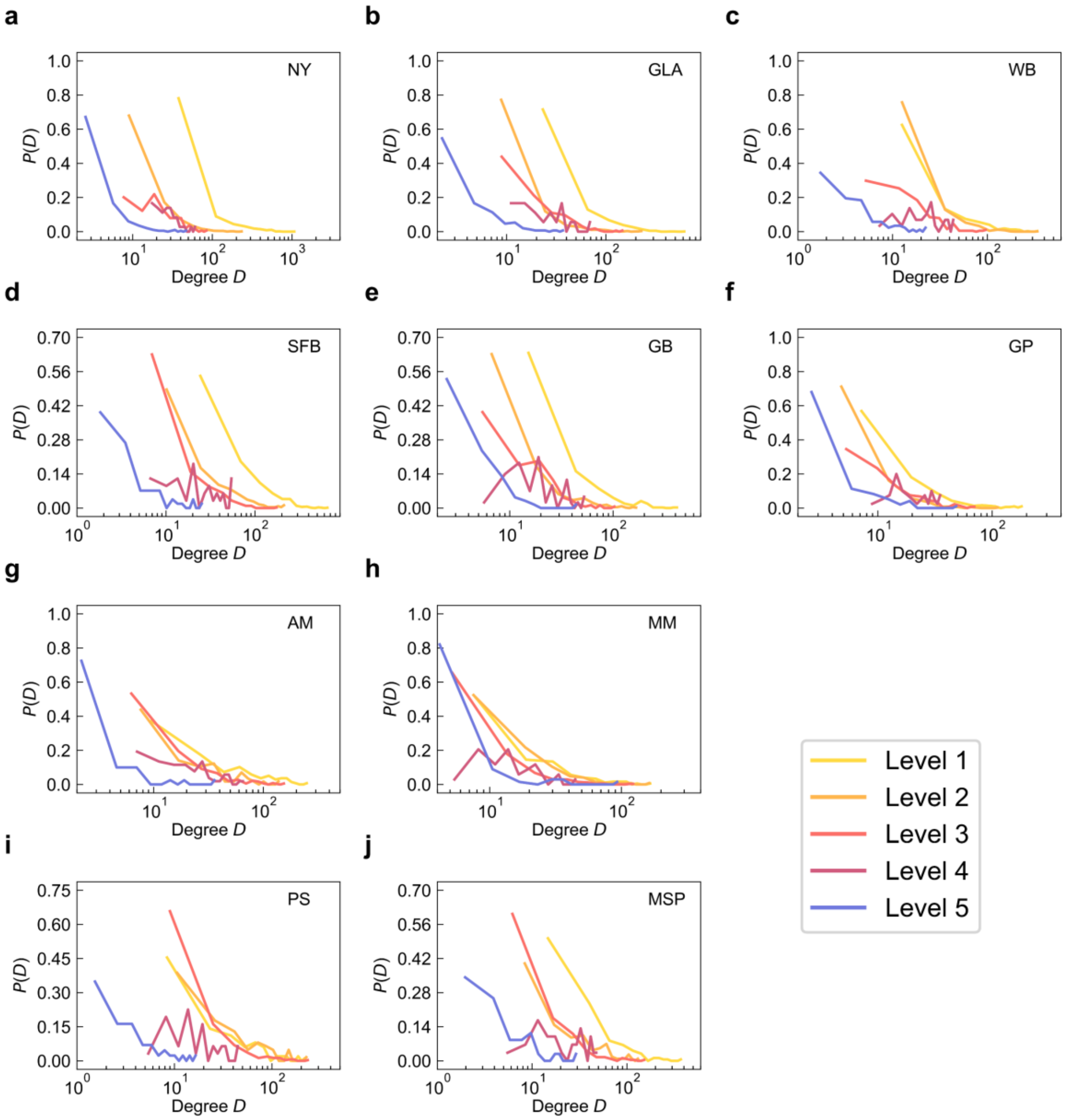

Figure S10. Travel degree distribution of locations across different segregation levels for all large cities.

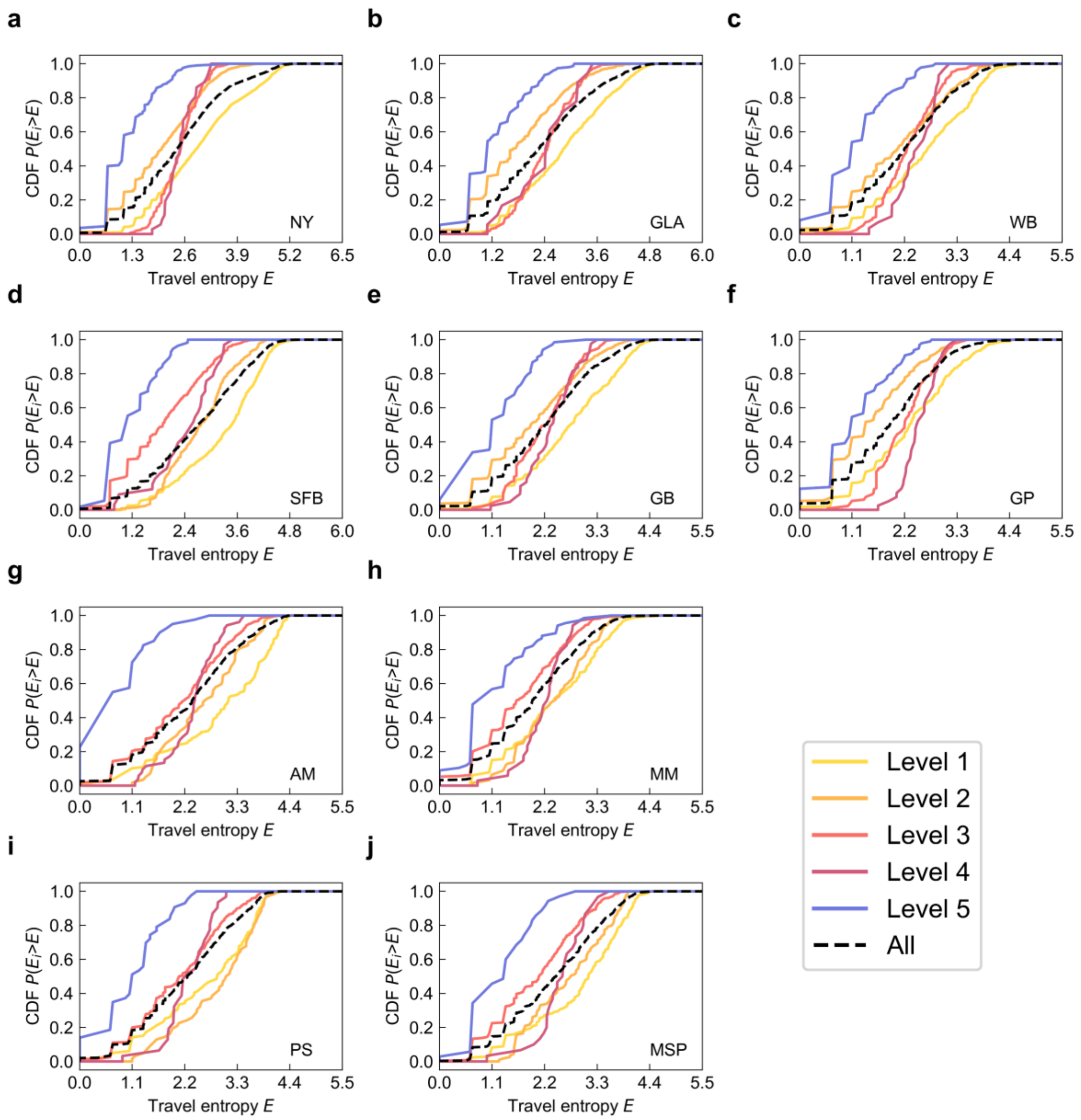

Figure S11. Travel entropy distribution of locations across different segregation levels for all large cities. The black dashed line represents the travel entropy distribution for all locations.

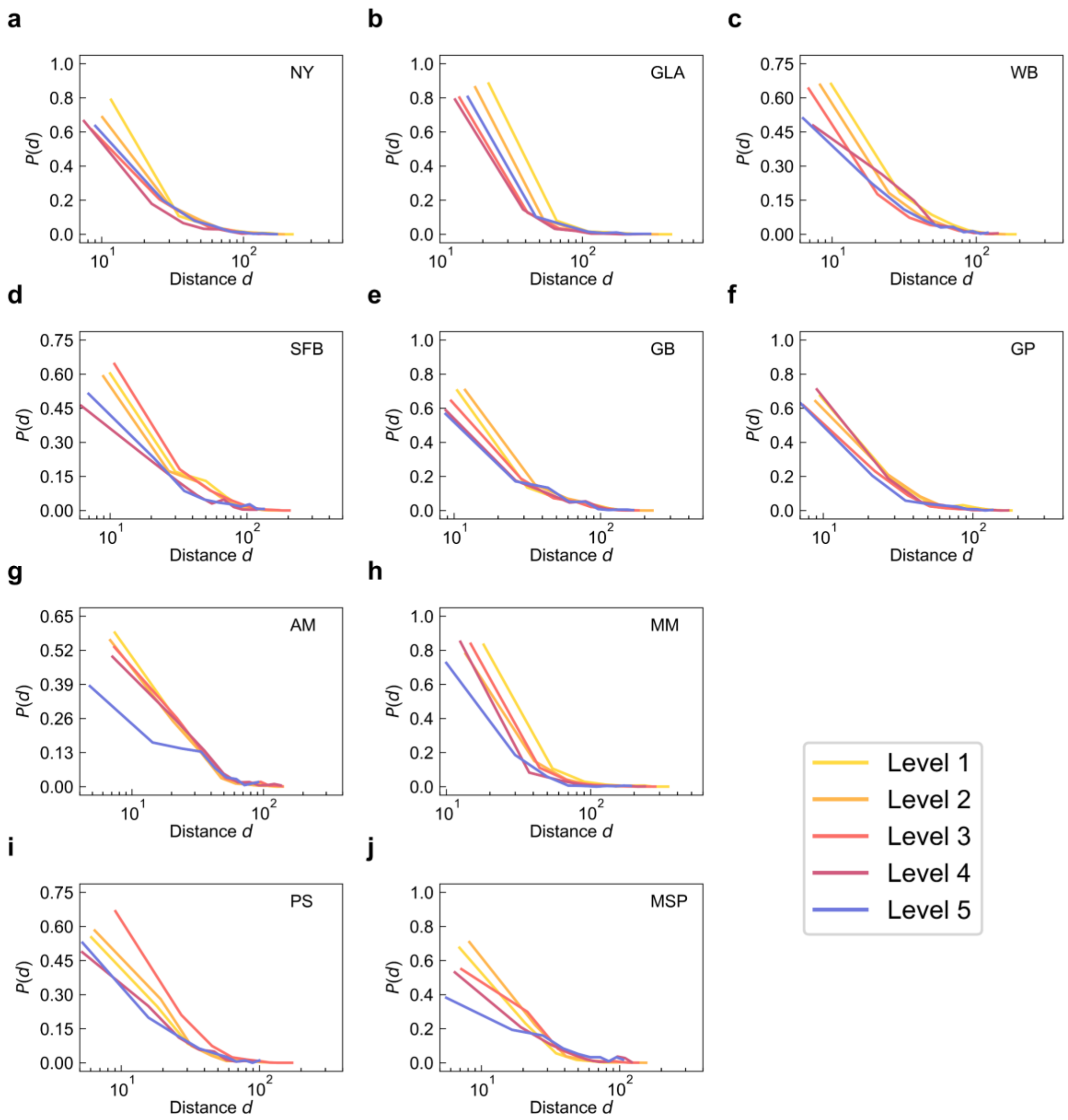

Figure S12. Travel distance distribution of locations across different segregation levels for all large cities.

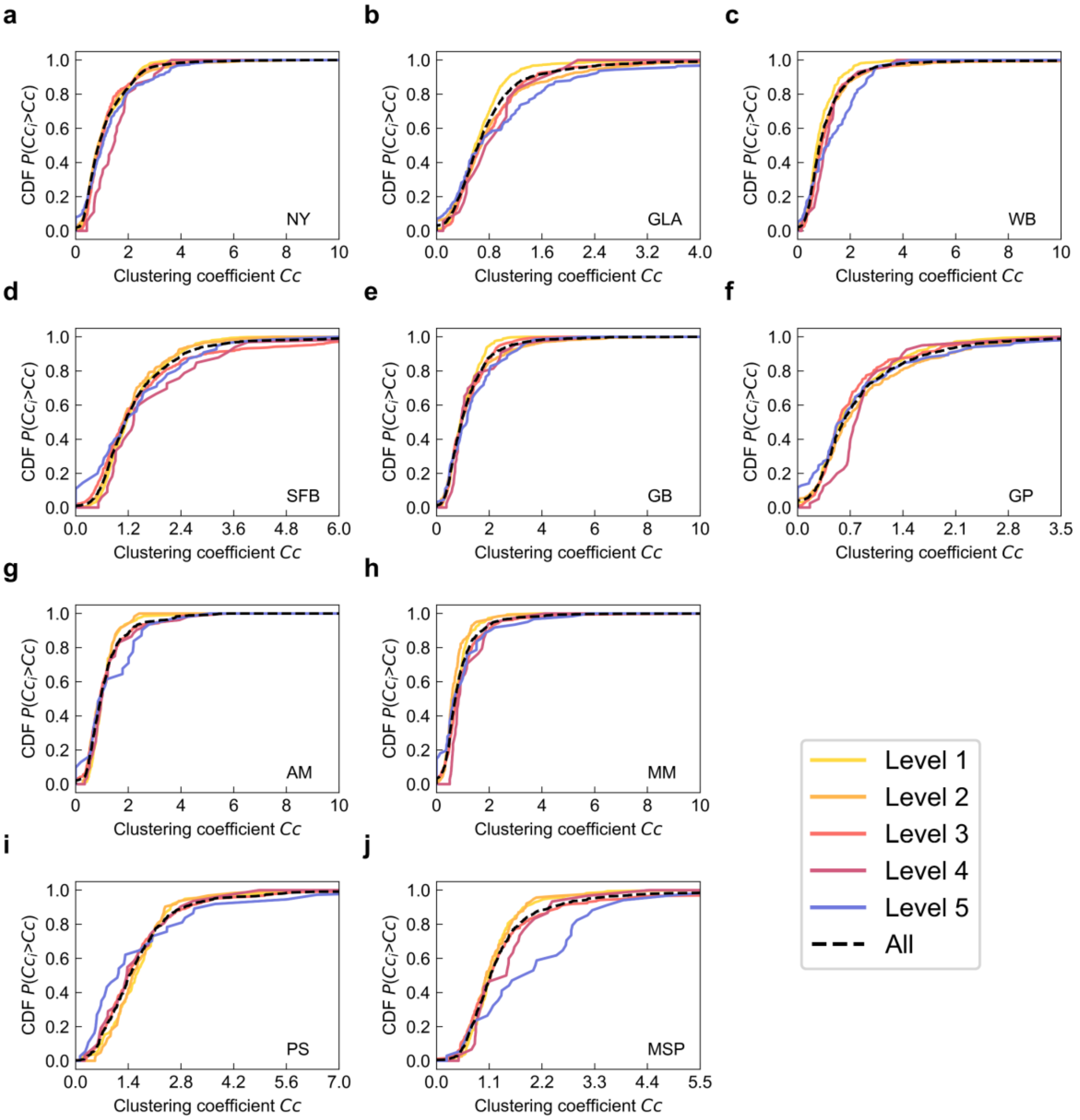

Figure S13. Clustering coefficient distribution of locations across different segregation levels for all large cities. The black dashed line represents the clustering coefficient distribution for all locations.

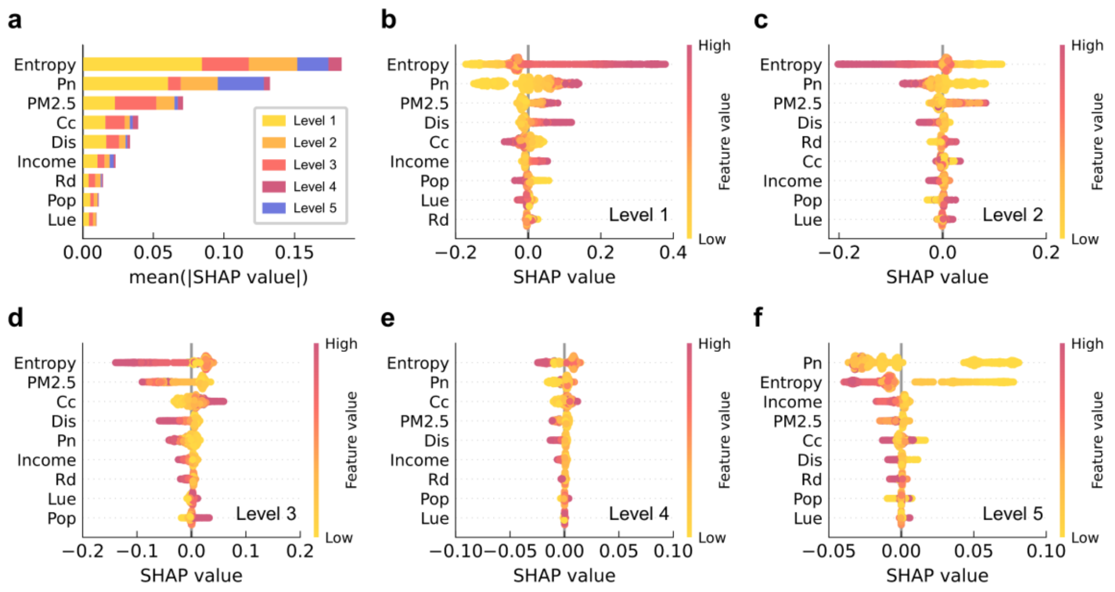

Figure S14. Impact of variables on segregation level classification. **a** Comparison assessment of the relative importance of features across segregation levels, with longer bars indicating greater significance. 'Pn', 'Pop', 'Rd', 'Dis', 'Cc', 'Lue', and 'PM2.5' denote the number of points of interest categories, population size, road density, travel distance, clustering coefficient, land use entropy, and local annual PM2.5 emissions, respectively. **b-f** Distribution of SHAP values of all features within each segregation level. Each scatter point represents a census tract, with color indicating feature value. Points positioned to the right of the x-axis signify a positive impact on model output, and vice versa.

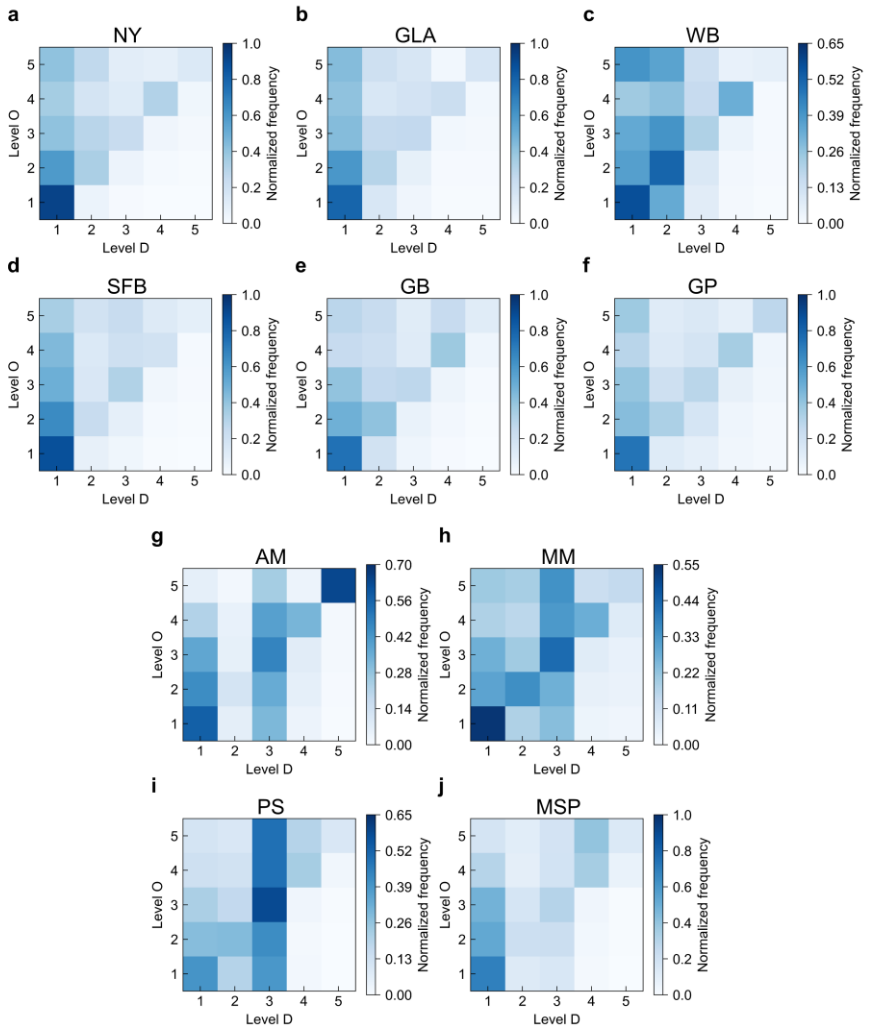

Figure S15. Segregation-constrained visitation matrix for 10 large cities. Color represents the visitation frequency between each segregation level, which is normalized based on the total number of trips from the origin level.

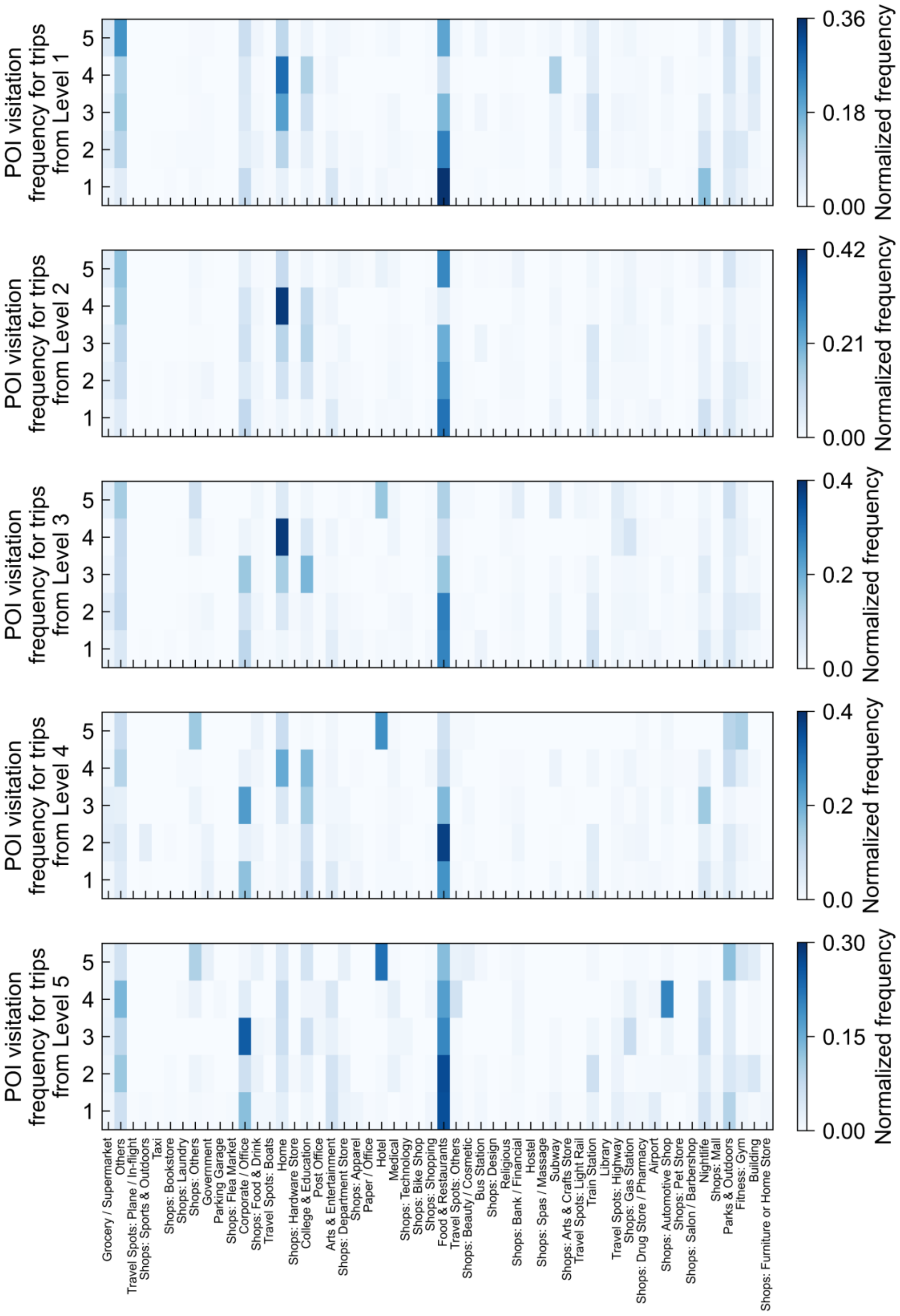

Figure S16. Distribution of POI visitation frequencies for trips from locations at a given segregation level to other locations with different segregation levels in the New York–Newark CSA. Color indicates the visitation frequencies between each segregation level, normalized based on the total number of trips to the destination level.

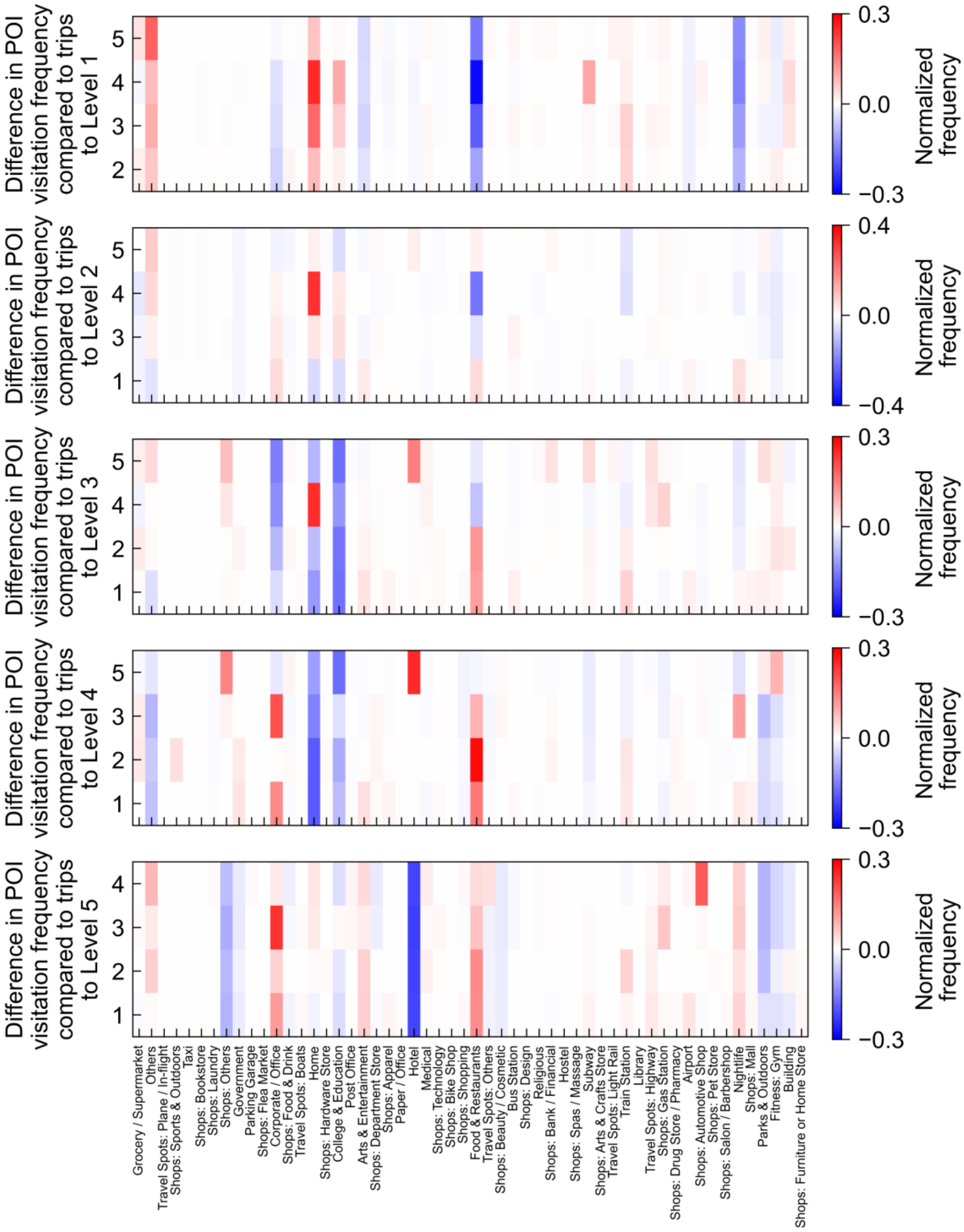

Figure S17. Differences in POI visitation frequencies for trips to locations with other segregation levels and to locations with the same segregation level as the departure location in the New York–Newark CSA.

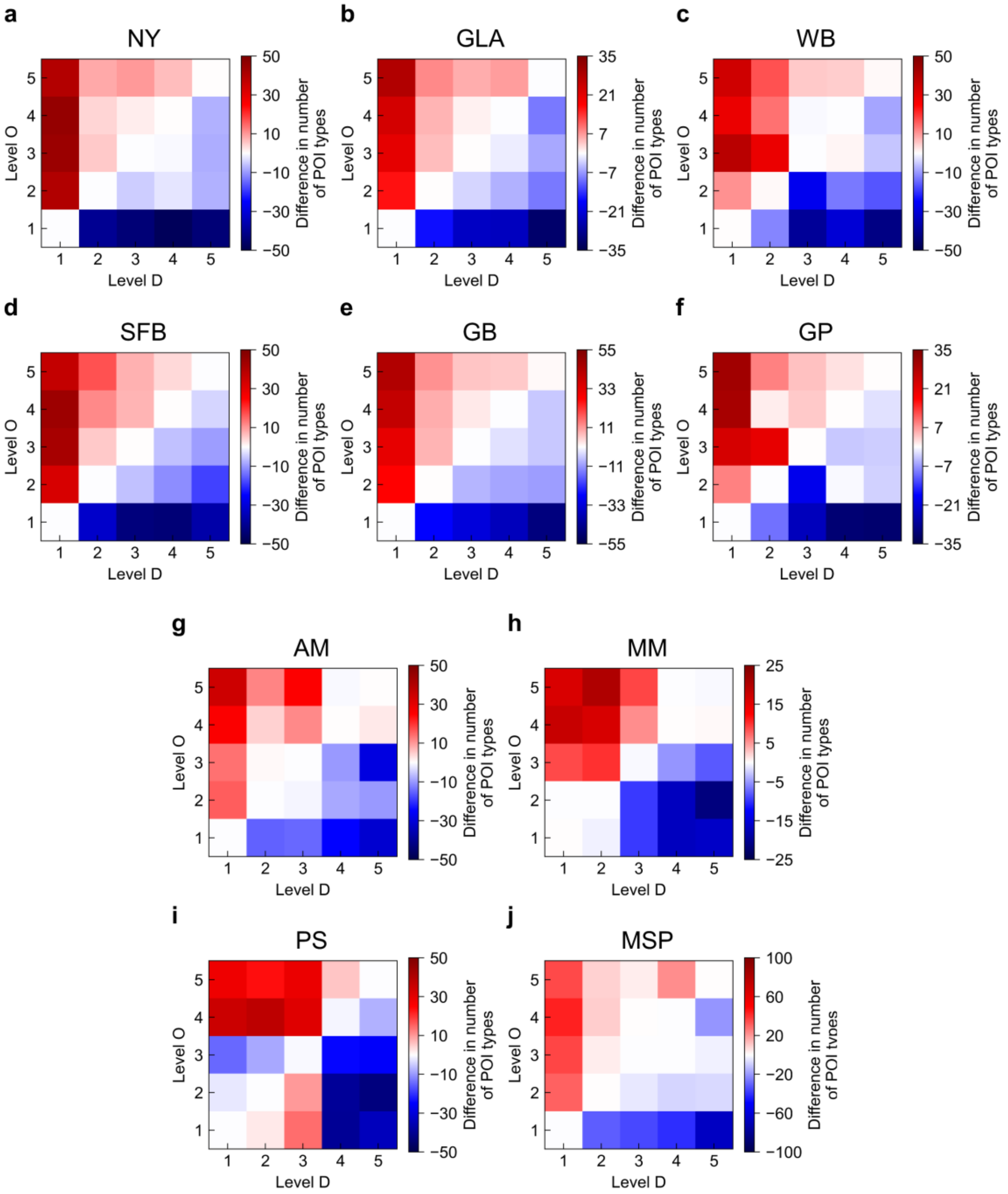

Figure S18. Differences in the number of POI types when traveling from origins with different segregation levels to destinations with different segregation levels. The color represents the average difference in the number of POI types at the destination compared to the origin.

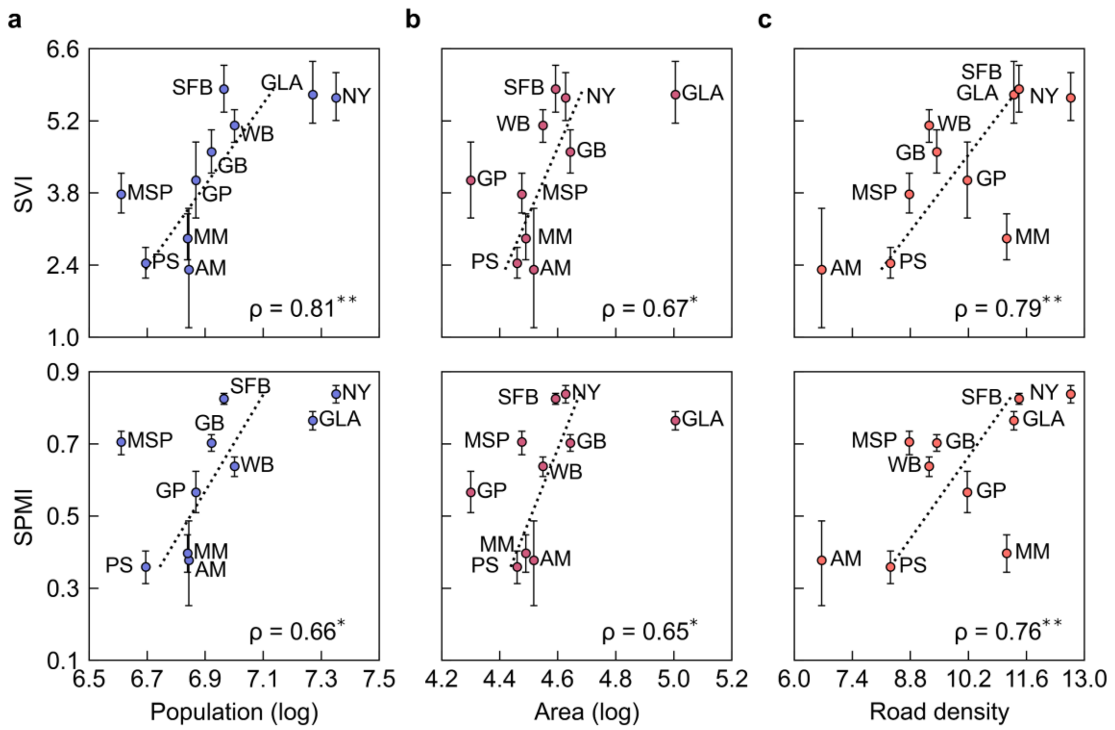

Figure S19. Correlation between two segregation visitation indices and three urban indicators. Panels show the correlation with: **a** Population, **b** Area, **c** Road density. ρ denotes the Spearman correlation coefficient. ** and * indicate that p-value is less than 0.01 and 0.05, respectively.

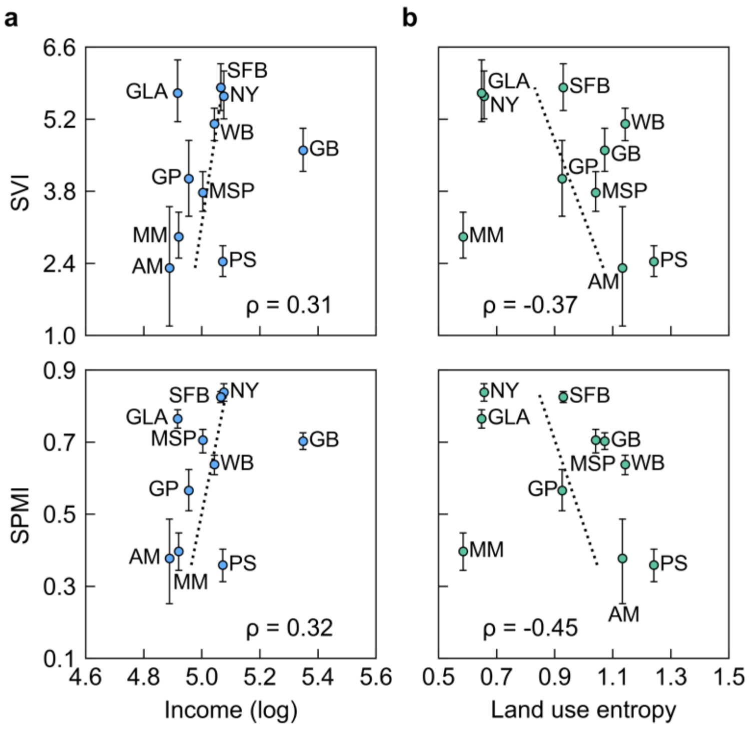

Figure S20. Correlation between two segregation visitation indices and two urban indicators. Panels show the correlation with: **a** Income, **b** Land use entropy. ρ denotes the Spearman correlation coefficient.

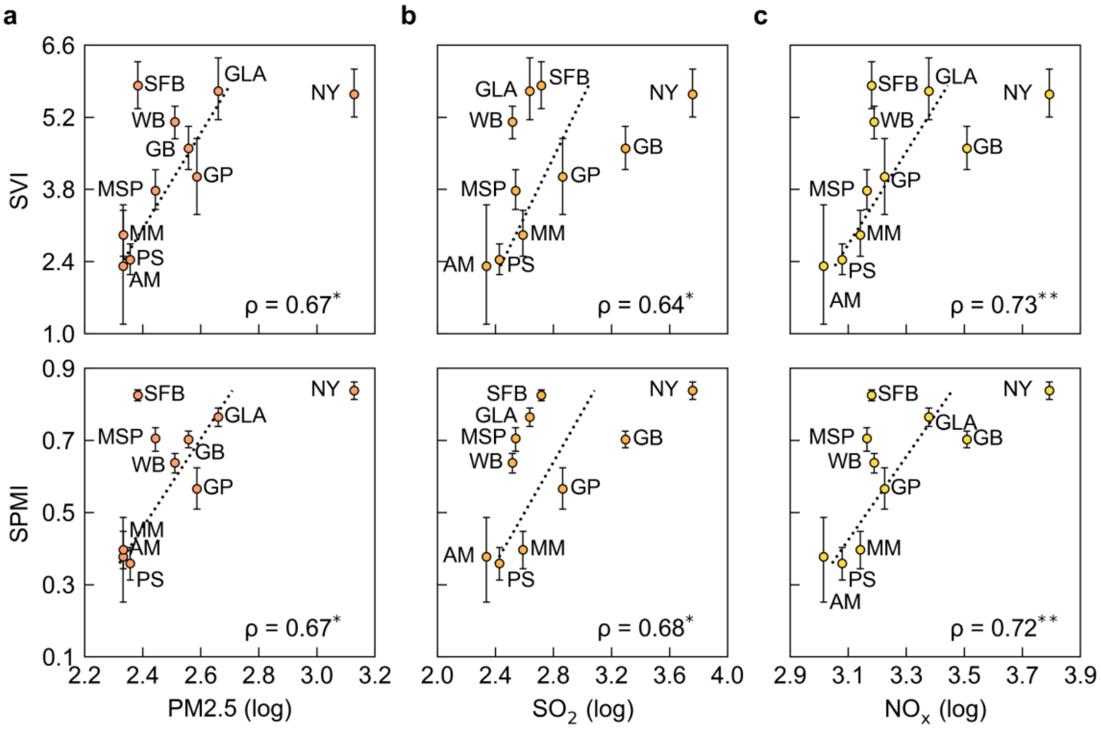

Figure S21. Correlation between two segregation visitation indices and two urban indicators. Panels show the correlation with: **a** PM2.5 emissions, **b** SO_2 emissions, **c** NO_x emissions. Pollutant emissions are the total annual emissions in tons. ρ denotes the Spearman correlation coefficient. ** and * indicate that p-value is less than 0.01 and 0.05, respectively.

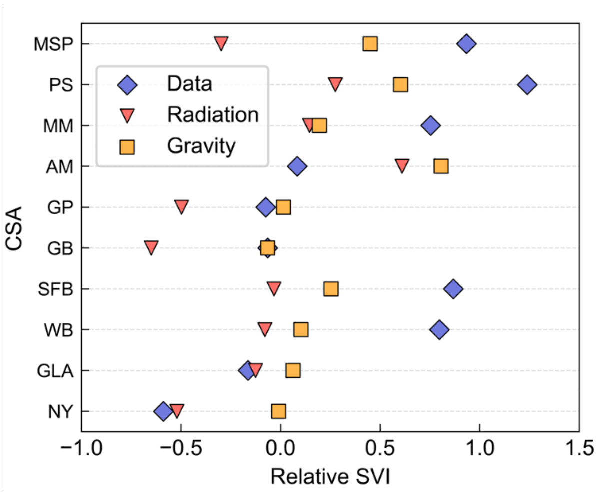

Figure S22. The relative difference between the SVI values of empirically observed (i.e., Data), radiation-based, gravity-based models (see Note S5 for details), and the SVI values of the null-based model.

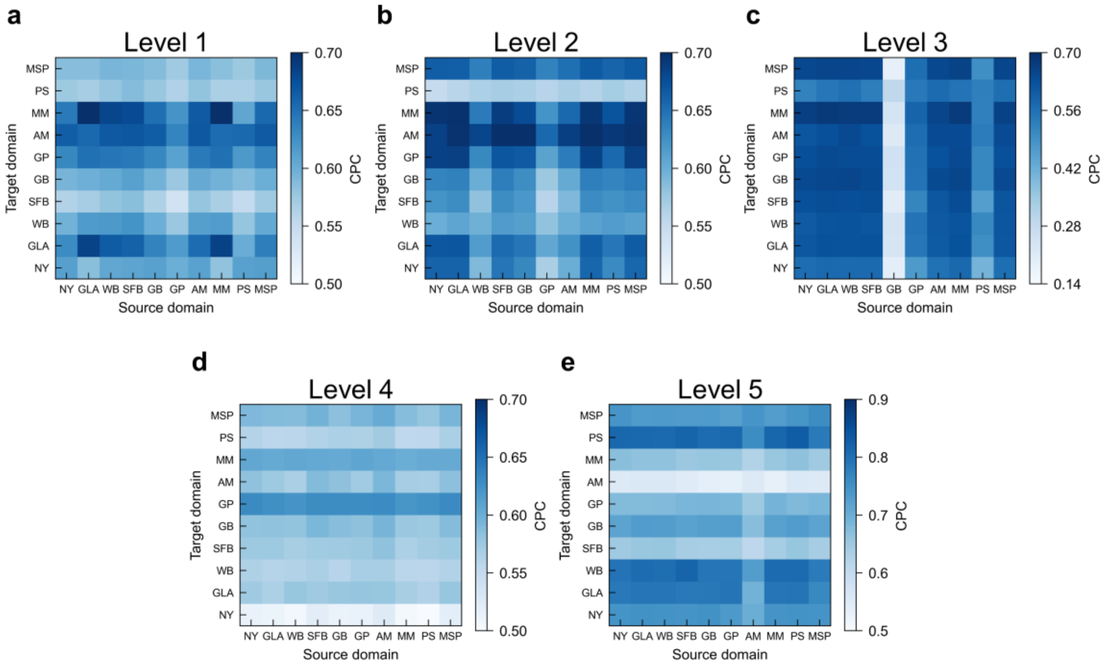

Figure S23. Transfer prediction performance for collective human flows at locations with the same segregation level between different large cities.

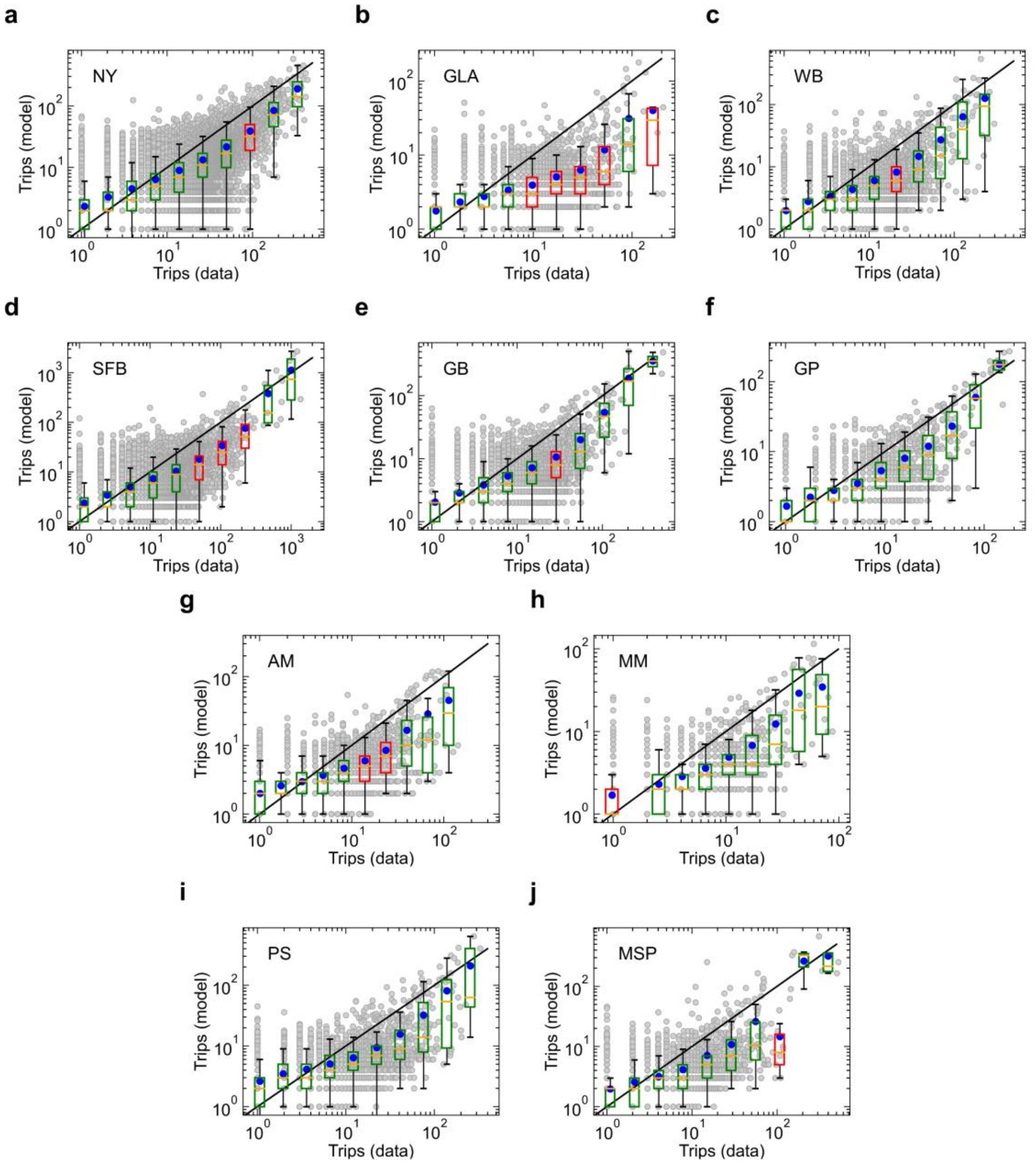

Figure S24. Paired comparisons of predicted and real trips for 10 large cities. Gray points indicate observed and predicted location pairs. The boxplot illustrates how the predicted trips are distributed across various ranges of observed trip counts. A green-shaded box indicates that the diagonal line $y = x$ falls within the 5th and 95th percentiles, and red otherwise. Blue points represent the average predicted trip counts across different bins.

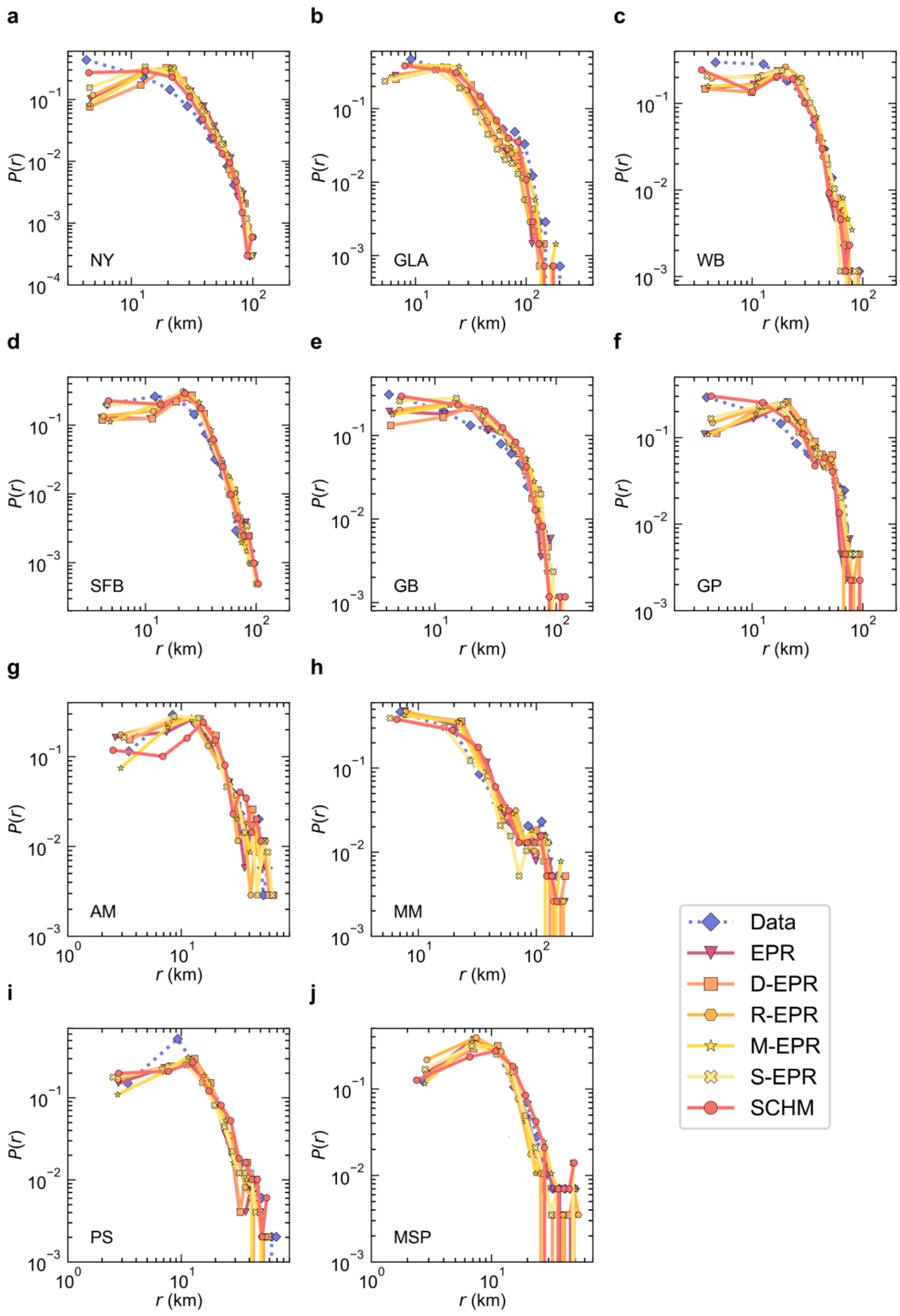

Figure S25. Comparison of radius of gyration distributions for 10 large cities.

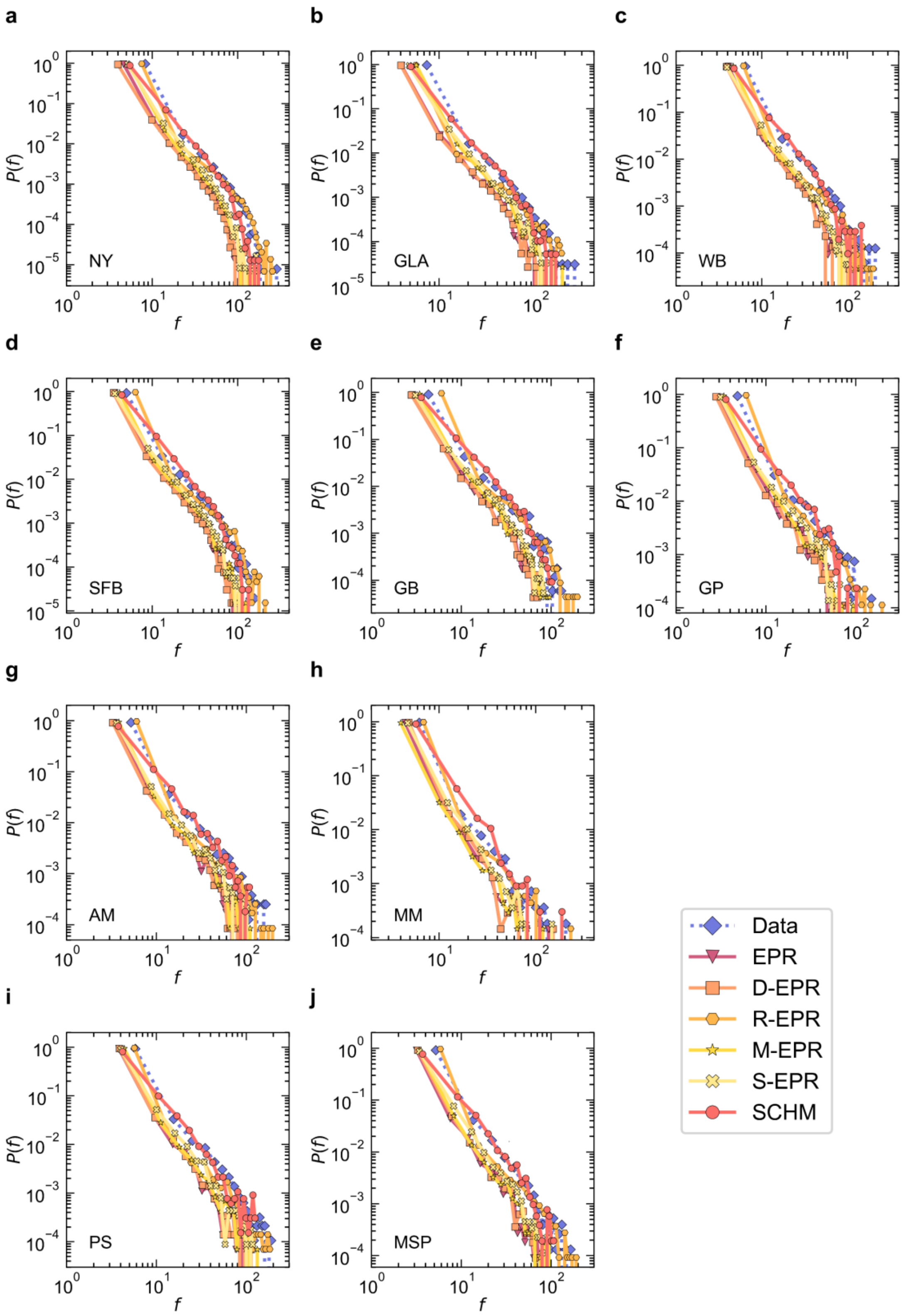

Figure S26. Comparison of distributions of individuals' location visitation frequency for 10 large cities.

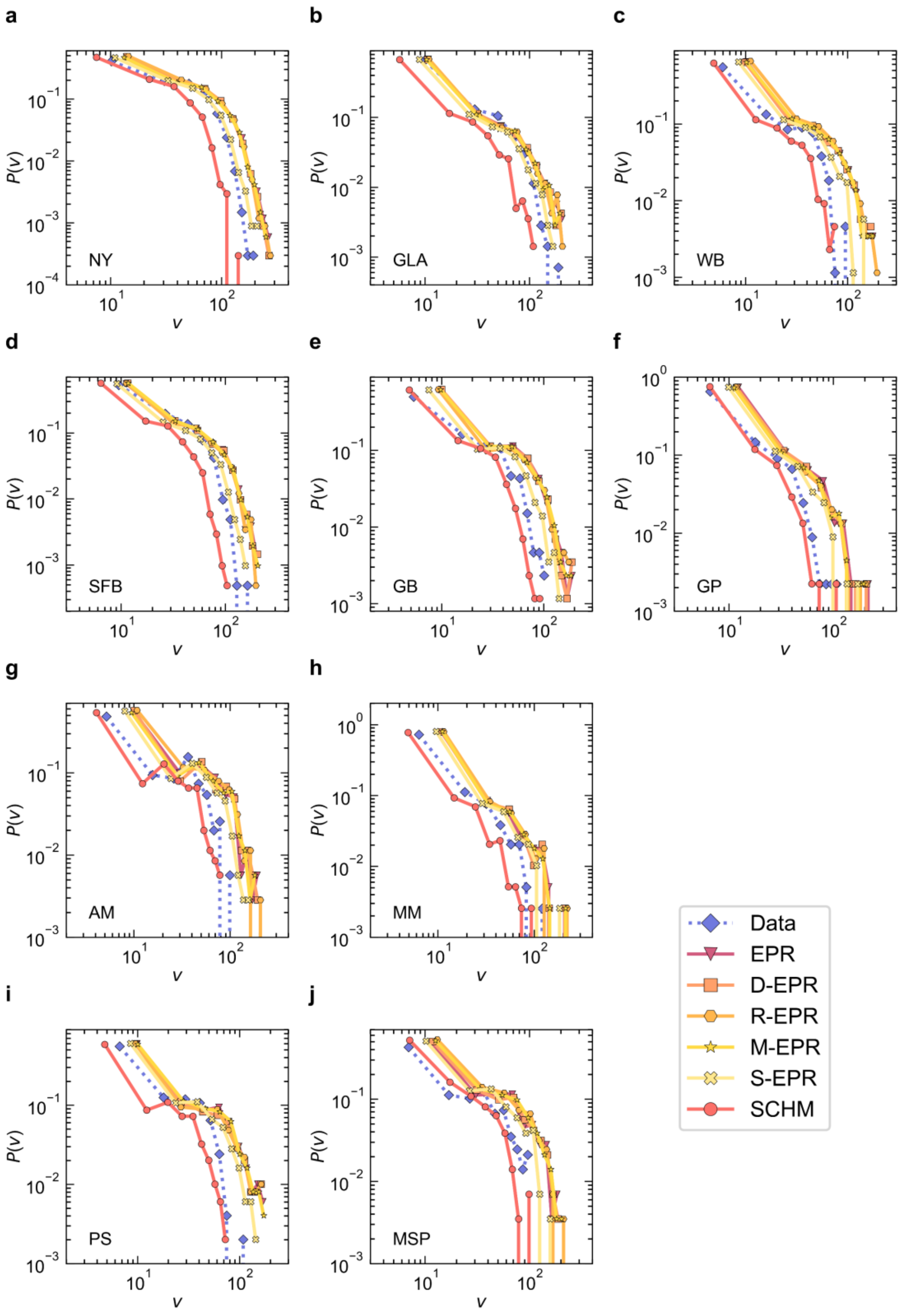

Figure S27. Comparison of distributions of the number of locations visited by individuals for 10 large cities.

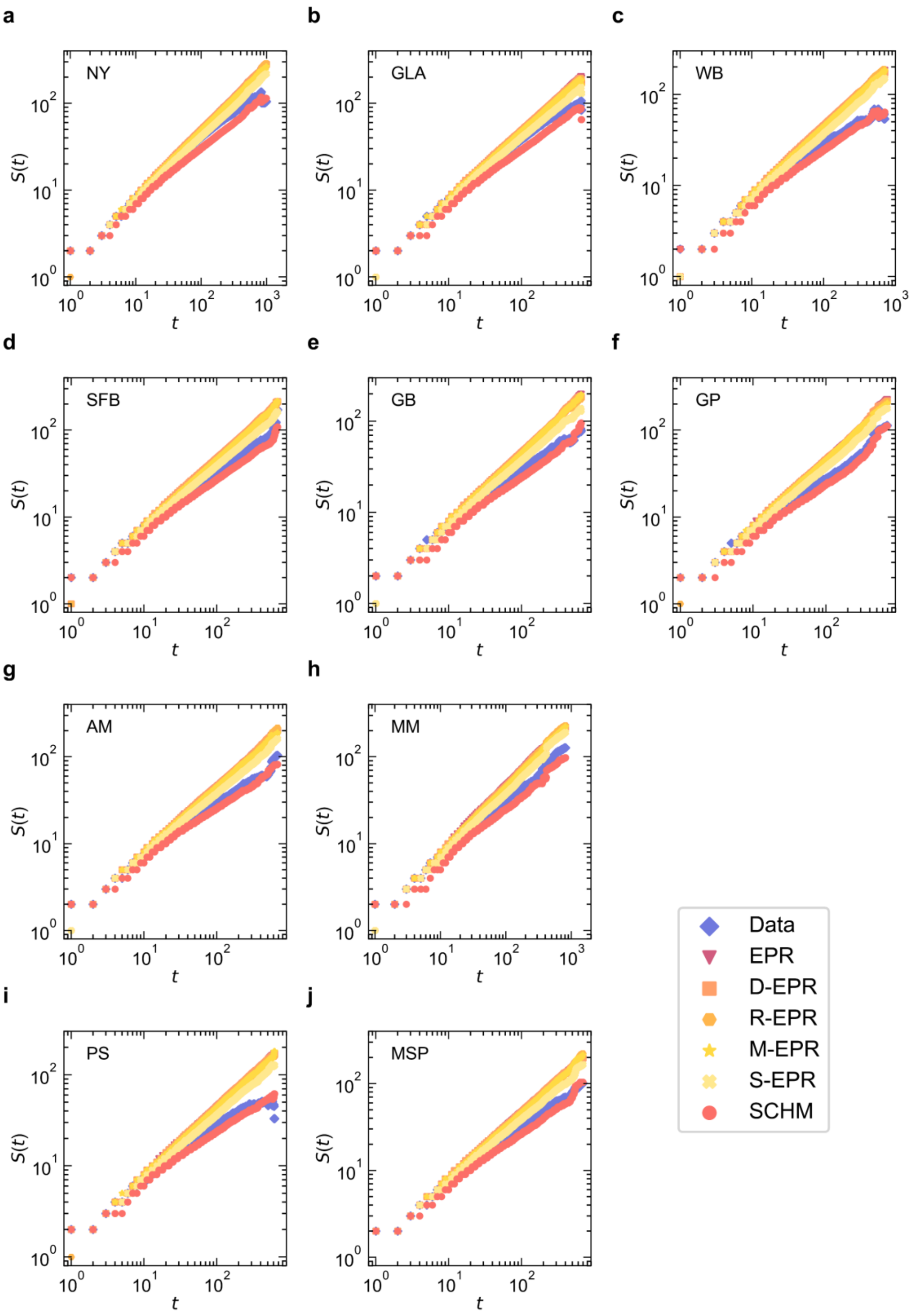

Figure S28. Comparison of distributions of the total number of locations visited within t trips for 10 large city datasets.

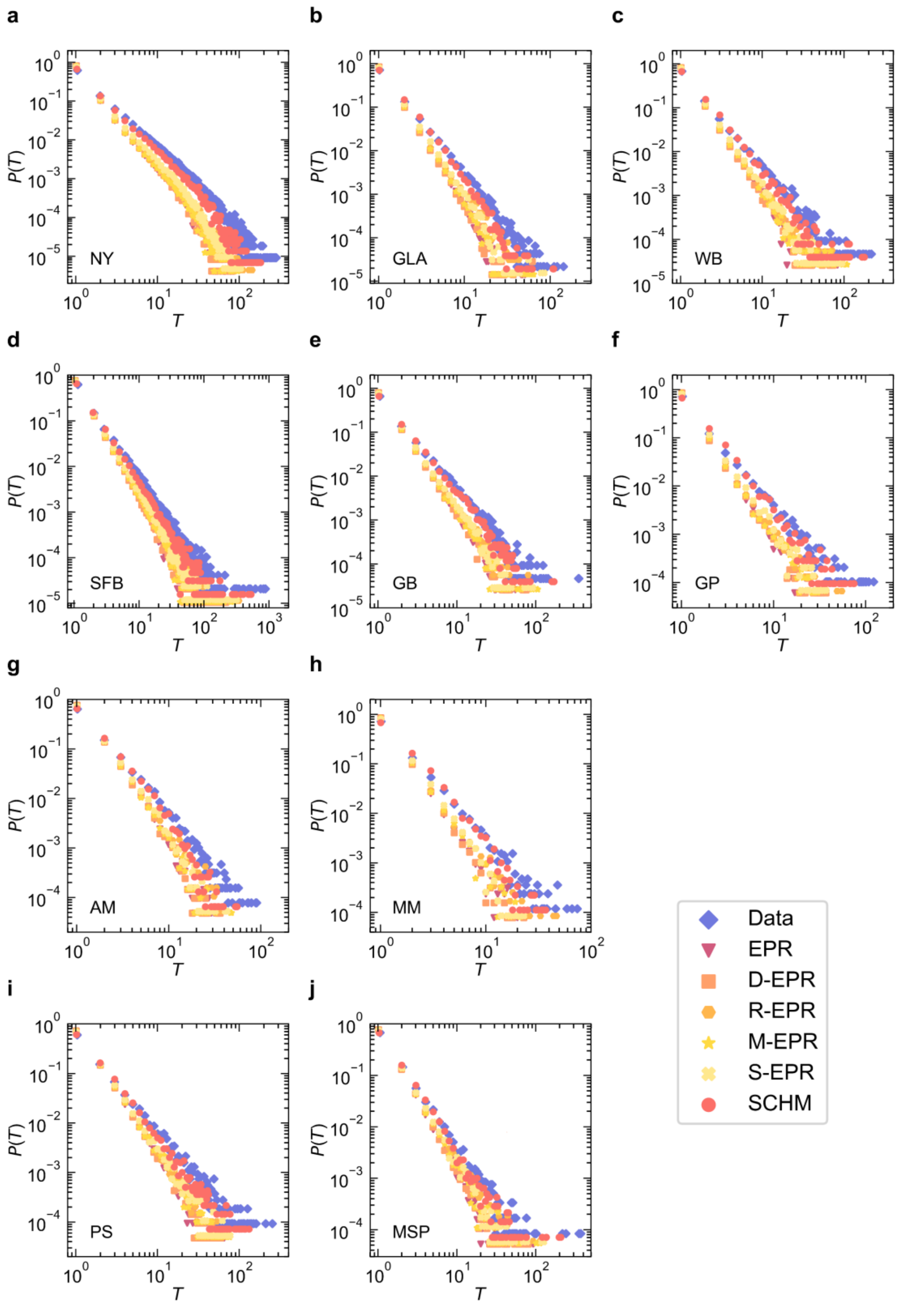

Figure S29. Comparison between predicted and actual distributions of the number of trips between two locations for 10 large cities.

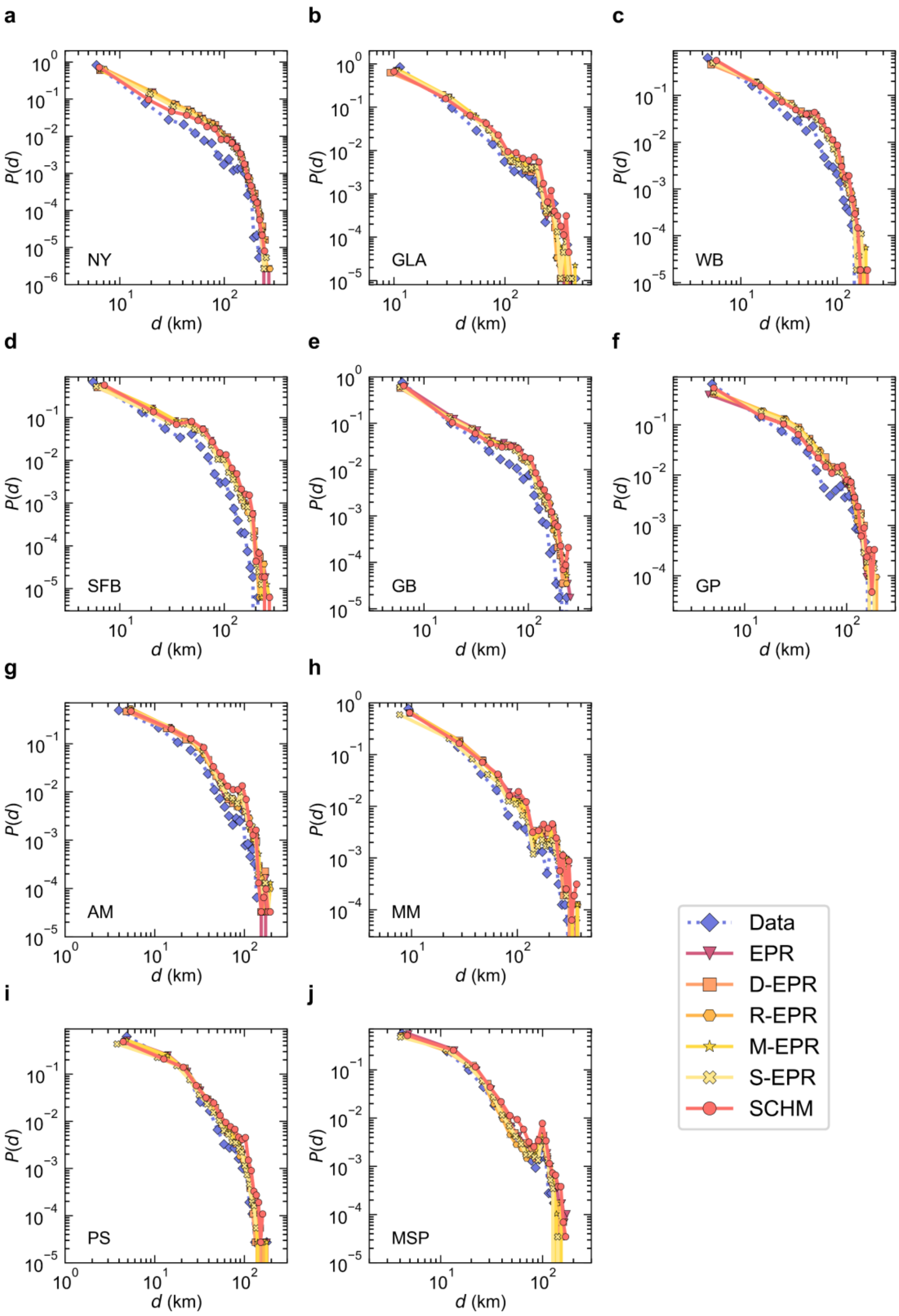

Figure S30. Comparison of travel distance distributions for 10 large cities.

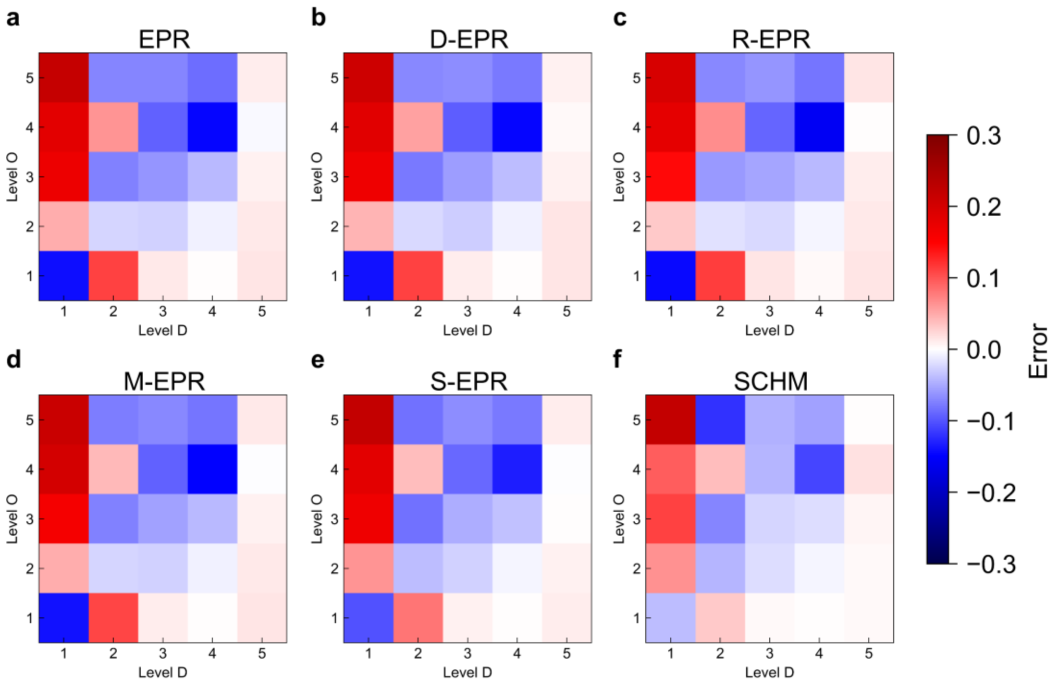

Figure S31. Visitation frequency error between locations with different segregation levels in the New York-Newark CSA. The color of the matrix is red (blue), indicating that the predicted value is greater (less) than the true value.

Supplemental tables

Table S1. Data statistics of the combined statistical area

Combined statistical area	Abb.	County number	Tract number	Population
New York–Newark	NY	31	5,310	22,431,833
Los Angeles–Long Beach	GLA	6	4,015	18,644,680
Washington–Baltimore–Arlington	WB	39	2,168	10,028,331
San Jose–San Francisco–Oakland	SFB	14	1,934	9,225,160
Boston–Worcester–Providence	GB	19	1,768	8,349,768
Philadelphia–Reading–Camden	GP	15	1,705	7,379,700
Atlanta–Athens-Clarke County–Sandy Springs	AM	41	1,105	6,976,171
Miami–Port St. Lucie–Fort Lauderdale	MM	7	1,362	6,908,296
Seattle–Tacoma	PS	8	892	4,953,421
Minneapolis–St. Paul	MSP	21	861	4,078,788

Note: "Abb." stands for abbreviation.

Table S2. Representativeness of segregation values calculated based on income quantiles of the sample and population

CSA name	NY	GLA	WB	SFB	GB	GP	AM	MM	PS	MSP
Coverage	65.0%	59.0%	79.0%	73.0%	63.7%	51.7%	62.2%	62.7%	65.8%	72.1%
PCC	0.58	0.44	0.76	0.75	0.55	0.52	0.48	0.52	0.60	0.70
p-value	0.00	0.00	0.00	0.00	0.00	0.00	0.00	0.00	0.00	0.00

Note: "PCC" denotes the Pearson correlation coefficient.

Table S3. Pearson correlation coefficients between segregation values calculated using quartiles and those obtained using other quantile interval settings (i.e., tertiles and quintiles)

CSA name	NY	GLA	WB	SFB	GB	GP	AM	MM	PS	MSP
Quartiles - tertiles	0.79	0.79	0.78	0.79	0.78	0.77	0.75	0.74	0.79	0.76
p-value	0.00	0.00	0.00	0.00	0.00	0.00	0.00	0.00	0.00	0.00
Quartiles-quintiles	0.85	0.86	0.85	0. 86	0.85	0.85	0.83	0.85	0.86	0.85
p-value	0.00	0.00	0.00	0.00	0.00	0.00	0.00	0.00	0.00	0.00

Table S4. Pearson correlation coefficients between segregation values calculated based on the segregation metric used in this study and those obtained from alternative segregation metrics

CSA name	NY	GLA	WB	SFB	GB	GP	AM	MM	PS	MSP
Euclidean distance	0.955	0.953	0.953	0.952	0.951	0.954	0.952	0.953	0.951	0.953
p-value	0.00	0.00	0.00	0.00	0.00	0.00	0.00	0.00	0.00	0.00
Entropy	-0.979	-0.979	-0.979	-0.979	-0.979	-0.979	-0.979	-0.980	-0.979	-0.980
p-value	0.00	0.00	0.00	0.00	0.00	0.00	0.00	0.00	0.00	0.00

Table S5. Confidence intervals of the segregation visitation index estimated using segregation values derived from different segregation metrics

CSA name	Euclidean distance-based metric	Entropy-based metric	Our used metric
NY	[-6.14, -5.21]	[-7.33, -6.29]	[-7.22, -5.93]
GLA	[-6.35, -5.15]	[-7.94, -6.79]	[-5.35, -4.49]
WB	[-5.41, -4.78]	[-7.70, -6.78]	[-7.67, -6.56]
SFB	[-6.28, -5.37]	[-8.22, -7.12]	[-8.33, -7.22]
GB	[-5.02, -4.18]	[-6.75, -5.55]	[-6.79, -5.73]
GP	[-4.79, -3.31]	[-6.69, -5.20]	[-6.70, -5.02]
AM	[-3.50, -1.18]	[-7.38, -5.99]	[-7.27, -5.71]
MM	[-3.39, -2.50]	[-7.06, -6.05]	[-6.96, -5.98]
PS	[-2.74, -2.14]	[-7.71, -6.21]	[-7.52, -6.10]
MSP	[-4.18, -3.41]	[-6.18, -5.00]	[-6.37, -5.08]

Table S6. Confidence intervals of the segregation visitation index obtained from uncertainty estimation using different index functions

CSA name	Proposed index		SPMI	
	Confidence interval	Mean	Confidence interval	Mean
NY	[-6.14, -5.20]	-5.65	[-0.86, -0.81]	-0.84
GLA	[-6.35, -5.15]	-5.71	[-0.79, -0.74]	-0.77
WB	[-5.41, -4.78]	-5.11	[-0.66, -0.61]	-0.64
SFB	[-6.28, -5.37]	-5.82	[-0.84, -0.81]	-0.83
GB	[-5.02, -4.18]	-4.60	[-0.73, -0.68]	-0.70
GP	[-4.79, -3.31]	-4.04	[-0.62, -0.51]	-0.57
AM	[-3.50, -1.18]	-2.31	[-0.49, -0.25]	-0.38
MM	[-3.39, -2.50]	-2.92	[-0.45, -0.34]	-0.40
PS	[-2.74, -2.14]	-2.44	[-0.40, -0.31]	-0.36
MSP	[-4.18, -3.41]	-3.77	[-0.74, -0.67]	-0.71

Table S7. Spearman correlation of segregation values between travel flow and travel degree for 10 large cities

Mobility measure	Metric	NY	GLA	WB	SFB	GB	GP	AM	MM	PS	MSP
Flow	SPC	-0.31	-0.36	-0.26	-0.40	-0.31	-0.20	-0.27	-0.26	-0.31	-0.33
	p-value	0.00	0.00	0.00	0.00	0.00	0.00	0.00	0.00	0.00	0.00
Degree	SPC	-0.39	-0.43	-0.36	-0.49	-0.39	-0.26	-0.40	-0.31	-0.37	-0.36
	p-value	0.00	0.00	0.00	0.00	0.00	0.00	0.00	0.00	0.00	0.00
Income	PCC	0.35	0.20	0.20	0.16	0.16	0.10	0.31	0.08	0.18	0.10
	p-value	0.00	0.00	0.00	0.00	0.00	0.00	0.00	0.00	0.00	0.00

Note: "SPC" denotes the Spearman correlation coefficient. "PCC" denotes the Pearson correlation coefficient.

Table S8. Performance comparison of different models for 10 large cities

CSA	Metric	EPR	D-EPR	M-EPR	R-EPR	S-EPR	SCHM
NY	CPC	0.499	0.500	0.508	0.494	0.563	**0.636**
	RMSE	6.550	6.550	6.450	6.224	5.836	**4.688**
	MAE	2.376	2.379	2.351	2.431	2.217	**2.055**
GLA	CPC	0.378	0.373	0.391	0.383	0.422	**0.469**
	RMSE	3.068	3.051	2.916	3.016	2.824	**2.588**
	MAE	1.551	1.556	1.537	1.578	1.510	**1.494**
WB	CPC	0.437	0.435	0.457	0.436	0.486	**0.558**
	RMSE	4.967	4.962	4.689	4.745	4.512	**3.613**
	MAE	1.891	1.888	1.846	1.922	1.812	**1.720**
SFB	CPC	0.528	0.526	0.538	0.528	0.579	**0.640**
	RMSE	9.096	9.099	8.598	8.112	7.763	**5.546**
	MAE	2.296	2.298	2.265	2.338	2.152	**1.980**
GB	CPC	0.461	0.457	0.477	0.469	0.509	**0.567**
	RMSE	5.237	5.258	4.969	4.977	4.811	**3.935**
	MAE	1.957	1.966	1.920	1.973	1.870	**1.816**
GP	CPC	0.377	0.383	0.396	0.380	0.438	**0.513**
	RMSE	4.010	3.905	3.947	3.936	3.689	**2.928**
	MAE	1.738	1.723	1.711	1.771	1.660	**1.598**
AM	CPC	0.472	0.469	0.476	0.469	0.504	**0.528**
	RMSE	3.587	3.628	3.590	3.564	3.445	**3.270**
	MAE	1.759	1.774	1.759	1.808	1.726	**1.739**
MM	CPC	0.352	0.350	0.386	0.373	0.405	**0.636**
	RMSE	2.869	2.876	2.771	2.633	2.719	**4.688**
	MAE	1.552	1.550	1.506	1.538	1.492	**2.055**
PS	CPC	0.535	0.536	0.554	0.546	0.578	**0.633**
	RMSE	6.384	6.450	6.198	5.971	5.729	**4.645**
	MAE	2.314	2.314	2.267	2.306	2.197	**2.058**
MSP	CPC	0.470	0.469	0.488	0.484	0.517	**0.564**
	RMSE	5.581	5.739	5.325	5.072	4.964	**3.753**
	MAE	1.797	1.800	1.770	1.820	1.712	**1.645**

Note: Bold font represents the optimal value of each metric.

Table S9. Prediction error of cross-segregation level visitation probability

CSA	Metric	EPR	D-EPR	M-EPR	R-EPR	S-EPR	SCHM
NY	RMSE	0.091	0.089	0.091	0.087	0.071	**0.069**
	MAE	0.068	0.066	0.067	0.064	0.054	**0.048**
GLA	RMSE	0.051	0.057	0.050	0.049	0.051	**0.037**
	MAE	0.038	0.043	0.038	0.037	0.035	**0.025**
WB	RMSE	0.043	0.051	0.046	0.058	0.044	**0.027**
	MAE	0.031	0.037	0.034	0.043	0.033	**0.016**
SFB	RMSE	0.070	0.066	0.069	**0.063**	0.063	0.064
	MAE	0.046	0.045	0.046	0.042	0.044	**0.037**
GB	RMSE	0.084	0.081	0.078	0.073	0.064	**0.065**
	MAE	0.059	0.056	0.054	0.049	0.044	**0.042**
GP	RMSE	0.058	0.060	0.053	0.047	0.046	**0.025**
	MAE	0.043	0.045	0.039	0.033	0.037	**0.019**
AM	RMSE	0.052	0.047	0.048	0.048	0.057	**0.036**
	MAE	0.039	0.035	0.035	0.036	0.037	**0.022**
MM	RMSE	0.045	0.045	0.036	0.044	0.041	**0.030**
	MAE	0.034	0.033	0.027	0.032	0.031	**0.022**
PS	RMSE	0.047	0.048	0.046	0.051	0.058	**0.045**
	MAE	0.031	0.034	0.029	0.037	0.041	**0.028**
MSP	RMSE	0.066	0.077	0.071	0.071	0.059	**0.037**
	MAE	0.047	0.052	0.046	0.050	0.037	**0.025**

Supplemental notes

Note S1. Calculation of income segregation

The most commonly used method for calculating income segregation based on human mobility data is to assess whether a target location is uniformly visited by various income groups. Following previous empirical studies[1,2], the income segregation experienced by various locations within large cities can be calculated by

$$S_i = \frac{Q}{2Q-2}\sum_{q=1}^{Q} |\tau_{qi} - \frac{1}{Q}| \tag{1.1}$$

where S_i denotes the income segregation value experienced by location i. Q denotes the number of income quantiles, which is set to four in this study. τ_{qi} denotes the proportion of people with income quantile q who visit location i. If the visit ratio of different groups is $1/Q$, the segregation value of location i is zero, that is, it does not suffer from income segregation.

Note S2. Income representativeness used for segregation calculation

In this study, we determine the income segregation value of a location by evaluating the proportion of each income group that visits it. Meanwhile, we use the average income distribution of individuals active within the target city to establish quantiles for categorizing income levels. To ensure the robustness of our segregation values against the quantile definition, we conducted a correlation analysis comparing segregation values derived from the income quantile of the sample versus those of the overall population. Specifically, the sample refers to the average income distribution across all census tracts covered by active individuals in the target city. The population refers to the average income distribution across all census tracts in the target city. If the quantiles calculated from both distributions are similar, it indicates that the income distribution of the sample areas closely resembles that of the population, suggesting representativeness. Table S2 shows that our data achieve at least 60% coverage, where income levels correspond to the same bin as those based on the population data. The correlation between segregation values based on samples and population is strong, with almost all p-values approaching zero. It is important to note that the segregation value calculated based on the population uses the quantiles derived from the population's income distribution to categorize individual's income level, which is then used to calculate the segregation value. Given that our data sample represents only a fraction of the population, we further evaluated the variability of the income distribution and its impacts on the model results. We follow the pipeline of previous work[3] and use the bootstrapping method for representative statistical analysis of the sample's income distribution. We performed 1,000 iterations for each city's sample, generating 1,000 resampled datasets. As illustrated in Figure S4, the mean user income of these resampled sets closely aligns with the target population, with a 95% confidence interval indicating that the income level error is within 0.2 units. This confirms that our sample does not significantly deviate from the overall population[3].

Note S3. Sensitivity analysis for income segregation calculation

To assess the robustness of the income segregation calculation, we conducted sensitivity analyses with respect to both quantile interval settings and the choice of segregation metrics. First, we altered the

number of income quantile intervals (three, four, and five) and examined the correlation of segregation values across all locations (see Table S3). The results show that our segregation metric is largely insensitive to the number of quantile intervals: across cities, the segregation distributions remain highly correlated ($\mathrm{PCC} > 0.75,\ p \ll 0.05$). Second, with the quantile interval fixed at four, we compared segregation values derived from our metric with those calculated using two alternative measures—Euclidean distance–based and entropy-based segregation metrics, as shown in Table S4. We can also see that the segregation values obtained under different metrics exhibit strong correlations. The correlation between the segregation values calculated using our own metric, and the segregation values calculated using the Euclidean distance metric ($S_i^{euclidean} = \sum_{q=1}^{Q}\left(\tau_{qi} - \frac{1}{4}\right)^2$) and the segregation values calculated using entropy metric[4] ($S_i^{entropy} = \frac{1}{\log 4}\sum_{q=1}^{Q}\tau_{qi}\log(\tau_{qi})$) are greater than 0.95 ($p \ll 0.05$). Nevertheless, we still used our metric because it offers greater variability over the range of [0, 1], enabling finer discrimination of segregation levels across locations.

Note S4. Robustness analysis for segregation visitation index calculation

We further examined the robustness of the proposed segregation visitation index with respect to alternative segregation metrics. Specifically, we computed segregation values at each location using the three metrics described above, randomly sampled trips from each location, and constructed segregation-constrained visitation matrices to derive corresponding visitation indices. The confidence intervals of the resulting indices (see Table S5) show that our metric yields a more stable distribution, reflected by narrower confidence intervals than those from the Euclidean-based, and entropy-based metrics. Further, with the same number of quantile intervals and segregation metric settings, we introduced the socioeconomic preferential mobility index[5] (SPMI) as an alternative segregation index to test the reliability of the proposed segregation visitation index. Following the same sampling procedure, we calculate SPMI as follows:

$$SPMI = \frac{S_{lower} - S_{upper}}{S_{lower} + S_{upper}} \tag{1.2}$$

where S_{lower} (S_{upper}) denotes the total probability of trips from higher- (lower-) segregation origins to lower- (higher-) segregation destinations. An SPMI of 0 indicates exclusive within-level visits, while 1 (−1) reflects a complete preference for lower (higher) segregation destinations. As can be observed from Table S6, SPMI yields narrower confidence intervals because we considered the degree of across segregation levels in the proposed index, thus resulting in an index with a wider numerical range. We further performed correlation analyses between segregation indices (from both methods) and a range of urban and environmental variables (see Figures S19-S21). Both indices show comparable correlation strengths across variables, supporting the robustness of our findings. Notably, land-use entropy displays only weak correlation with the segregation visitation index, and average regional income levels are not strongly correlated with total segregation visitation intensity. These results suggest that income segregation patterns are shaped by more complex, multidimensional factors that extend beyond income alone.

Note S5. Metrics for mobility pattern analysis

We explore the variations in mobility patterns of groups at locations with different segregation levels, considering travel degree D, average travel distance d, travel entropy E, and travel clustering coefficient Cc (see Figures S10-S13). Specifically, travel degree indicates the number of different locations visited from a given location, while average travel distance represents the range of movement from that location. Travel entropy[6] represents the diffusion degree of travel flow starting from a location and is defined as follows:

$$E_i = -\sum_{j=1}^{N} p_{ij} \log_2 p_{ij} \tag{1.3}$$

where p_{ij} denotes the travel probability from location i to location j. N denotes the total number of locations visited from location i. A higher travel entropy indicates that people travel to a more diverse set of locations. Similarly, the travel clustering coefficient[7] describes the cliqueness or transitivity of people's travel network by measuring the presence of triangles in the travel network starting from location i. It is defined as follows:

$$Cc_i = \frac{1}{D_i(D_i - 1)} \sum_{j=1}^{N} \sum_{k=1}^{M} (p_{ij} p_{jk} p_{ki})^{1/3} \tag{1.4}$$

where D_i denotes the degree of node i. N and M denote the total number of locations visited from location i and location j, respectively. p_{ij}, p_{jk}, and p_{ki} represent the travel probabilities between the corresponding locations.

Note S6. SHapley Additive exPlanations (SHAP) analysis

We construct a segregation level classification model utilizing the XGBoost algorithm to investigate the correlations between different input variables and segregation levels. To thoroughly analyze each variable's impact on the segregation value across different segregation levels, and its contribution at each level, we employ the SHAP method[8] for visual analysis.

The SHAP method originates from the Shapley value concept grounded in cooperative game theory. SHAP assigns a "Shapley value" to each feature value by calculating its contribution to the predicted output across all possible permutations of the feature value. This value signifies the contribution of each feature to the final prediction, offering an intuitive and comprehensive way of interpreting the model output. Illustrated in Figure S14, we scrutinize the relationship between nine variables and five segregation levels. The color of the scatter points denotes the magnitude of the variable. If the scatter points appear on the right side of the x-axis, it implies a positive impact on the model output, and vice versa. The variables on the y-axis are arranged in descending order of importance. As shown in Figure S14, the direction and intensity of influence of each variable differ under different levels. Higher segregation levels are linked with larger clustering coefficients and smaller travel entropy, while lower segregation levels are associated with greater travel entropy and a more diverse range of POIs. These results affirm the heterogeneity of human mobility patterns under varying segregation levels and underscore the complexity of factors influencing income segregation.

Note S7. Reference models

To validate that the segregation-constrained human flow patterns described result from the biased

visitation behaviors of groups with different segregation levels, we introduce three reference models for comparative analysis. We obtain the total number of trips at each location based on observation data, and then maintain the total constant to allocate travel flows based on different rules, including null-based, gravity-based, and radiation-based. The null-based reference model indicates that different locations have an identical visitation probability. Gravity-based and radiation-based reference models utilize gravity and radiation models to define the visitation probability between locations, respectively. The gravity model and radiation model are defined as follows:

$$p_{ij}^{GM} = \frac{Pop_j^{\gamma_1}}{d_{ij}^{\gamma_2}} \tag{1.5}$$

$$p_{ij}^{RM} = \frac{Pop_i Pop_j}{(Pop_i + s_{ij})(Pop_i + s_{ij} + Pop_j)} \tag{1.6}$$

where $d_{ij}^{\gamma_2}$ represents the distance between location i and location j. Parameters γ_1 and γ_2 are to be estimated. Pop_i and Pop_j denote the population of location i and location j, respectively, while s_{ij} denotes the number of intervention opportunities between location i and location j. Consequently, we derive three travel networks based on the three reference models. Further, we obtain the corresponding segregation-constrained visitation matrix, respectively, and calculate the corresponding SVI value (see Figure S19).

Note S8. Predictability analysis of human mobility at different segregation levels

Recognizing the variations in collective mobility patterns in locations across different segregation levels, we construct gravity models based on different training strategies to further explore the predictability of mobility patterns at these levels. Initially, we utilize travel data from all levels within a large city to construct an origin-constrained gravity model[9], simultaneously estimating human flows between all locations, denoted as Global GM. On the other hand, we construct dedicated gravity models based on the travel data of each segregation level to estimate human flows at the single segregation level, denoted as Local GM. Meanwhile, for each model, we divided the travel data in a ratio of 8:2 for model training and testing. In the main text (**Figure 6c**), we compare the predictive performance of these two models. Due to the heterogeneous mobility patterns among various segregation levels, local GM exhibits superior estimation performance in comparison.

Note S9. Baseline models

To assess the trajectory generation performance of the segregation-constrained human mobility model (SCHM), we compare SCHM's performance with the exploration and preference return[10] (EPR) model and its four variant models, namely, gravity-based EPR[11] (D-EPR), recency-based EPR[12] (R-EPR), memory-based EPR[13] (M-EPR), and social EPR[1] (S-EPR).

In each step of selecting a location for travel, the EPR model opts to return to the previously visited location with a probability of $1 - \rho S^{-\gamma}$, and explore a new location with a probability of $\rho S^{-\gamma}$. In this process, the selection of the returned location is contingent on the visitation frequency. Parameters ρ and γ drawn from empirical research[10,14]. Concerning the variant models, the D-EPR model extends the EPR's exploration process, and enhances individual exploration accuracy by incorporating a gravity model. The R-EPR model extends the return stage of the EPR model, and improves individual return

selection accuracy based on frequency ranking $Rank_f(loc_i)^{-1-\gamma}$ and recency ranking $Rank_r(loc_i)^{-\eta}$ of location loc_i. Wherein parameters α, γ, and η are determined to be 0.6, 0.21, and 0.8 based on empirical research[12]. Similarly, the M-ERP model also enhances the return stage of the EPR model by constraining only the memory impact of the individual's recent M days. Moreover, the S-EPR model introduces individual income segregation visitation constraints by optimizing the exploration phase of the EPR model. That is, individuals decide with probability σ_s whether to visit a location where the income level of the vast majority of people is not comparable to their own, or with probability $1-\sigma_s$ to visit a location where the income level of the vast majority of people is comparable to their own. Meanwhile, σ_s denotes the proportion of places visited by the user where their income group is the minority.

Note S10. Performance evaluation

Three widely used metrics are used to measure the model performance, including the common part of commuters[9] (CPC), mean absolute error (MAE), and root mean squared error (RMSE):

$$\mathrm{CPC} = \sum_{i}^{N}\sum_{j\neq i}^{N}\frac{2\times\min\{T_{ij},T'_{ij}\}}{\sum_{i,j}T_{ij}+\sum_{i,j}T'_{ij}} \tag{2.1}$$

$$\mathrm{MAE} = \frac{1}{N(N-1)}\sum_{i}^{N}\sum_{j\neq i}^{N}|T_{ij}-T'_{ij}| \tag{2.2}$$

$$\mathrm{RMSE} = \sqrt{\frac{1}{N(N-1)}\sum_{i}^{N}\sum_{j\neq i}^{N}\left(T_{ij}-T'_{ij}\right)^2} \tag{2.3}$$

where T_{ij} and T'_{ij} denote the actual and predicted trips from location i to location j, respectively. N denotes the total number of locations.

Supplemental references

1. Moro, E., Calacci, D., Dong, X., and Pentland, A. (2021). Mobility patterns are associated with experienced income segregation in large US cities. Nat. Commun. *12*, 4633. https://doi.org/10.1038/s41467-021-24899-8.
2. Sun, C., Shibuya, Y., and Sekimoto, Y. (2024). Social segregation levels vary depending on activity space types: Comparison of segregation in residential, workplace, routine and non-routine activities in Tokyo metropolitan area. Cities *146*, 104745. https://doi.org/10.1016/j.cities.2023.104745.
3. Hilman, R. M., Iñiguez, G., and Karsai, M. (2022). Socioeconomic biases in urban mixing patterns of US metropolitan areas. EPJ Data Sci. *11*, 32. https://doi.org/10.1140/epjds/s13688-022-00341-x.
4. Massey, D. S., and Denton, N. A. (1988). The dimensions of residential segregation. Soc. forces *67*, 281–315. https://doi.org/10.1093/sf/67.2.281.
5. Liao, Y., Gil, J., Yeh, S., Pereira, R. H., and Alessandretti, L. (2025). Socio-spatial segregation and human mobility: A review of empirical evidence. Comput. Environ. Urban Syst. *117*, 102250. https://doi.org/10.1016/j.compenvurbsys.2025.102250.
6. Mowshowitz, A. (1968). Entropy and the complexity of graphs: I. An index of the relative complexity of a graph. Bull. Math Biophys. *30*, 175–204. https://doi.org/10.1007/BF02476948.
7. Onnela, J. P., Saramäki, J., Kertész, J., and Kaski, K. (2005). Intensity and coherence of motifs in weighted complex networks. Phys. Rev. E *71*, 065103. https://doi.org/10.1103/PhysRevE.71.065103.
8. Lundberg, S. M., and Lee, S. I. (2017) A unified approach to interpreting model predictions. In Proc. 31st Conf. on Neural Inf. Process. Syst. pp. 4768–4777.
9. Barbosa, H., Barthelemy, M., Ghoshal, G., James C., Lenormand, M., Louail, T., Menezes, R., Ramasco, J., Simini, F., and Tomasini, M. (2018). Human mobility: Models and applications. Phys. Rep. *734*, 1–74. https://doi.org/10.1016/j.physrep.2018.01.001.
10. Song, C., Koren, T., Wang, P., and Barabási, A.L. (2010). Modelling the scaling properties of human mobility. Nat. Phys. *6*, 818–823. https://doi.org/10.1038/nphys1760.
11. Pappalardo, L., Simini, F., Rinzivillo, S., Pedreschi, D., Giannotti, F., and Barabási, A.L. (2015). Returners and explorers dichotomy in human mobility. Nat. Commun. *6*, 8166. https://doi.org/10.1038/ncomms9166.
12. Barbosa, H., de Lima-Neto, F.B., Evsukoff, A., and Menezes, R. (2015). The effect of recency to human mobility. EPJ Data Sci. *4*, 21. 10.1140/epjds/s13688-015-0059-8.
13. Alessandretti, L., Sapiezynski, P., Sekara, V., Lehmann, S., and Baronchelli, A. (2018). Evidence for a conserved quantity in human mobility. Nat. Hum. Behav. *2*, 485–491. https://doi.org/10.1038/s41562-018-0364-x.
14. Song, C., Koren, T., Wang, P., and Barabási, A.L. (2010). Modelling the scaling properties of human mobility. Nat. Phys. *6*, 818–823. https://doi.org/10.1038/nphys1760.
