# Supplementary material for: Promoting sustainable human mobility for income segregation mitigation
Source: Patterns (N Y). 2026 Mar 2;7(3):101477. doi: 10.1016/j.patter.2025.101477 (PMC13100684; doi:10.1016/j.patter.2025.101477)
Supplement: Document S2. Article plus supplemental information [file mmc2.pdf]

Article

Patterns

Promoting sustainable human mobility for income segregation mitigation

Highlights

- Reveal biased travel patterns from highly segregated to less segregated areas
- Propose a segregation visitation index to quantify biased human mobility
- Develop a segregation-constrained mobility model capturing structural flow dynamics
- Reveal interplays among urban income segregation, mobility, and pollution exposure

Authors

Yong Chen, Chenlei Liao, Zeen Cai, ..., Xiqun (Michael) Chen, Jianjun Wu, Ziyou Gao

Correspondence

chenxiqun@zju.edu.cn (X.C.),
zygao@bjtu.edu.cn (Z.G.)

In brief

Despite extensive studies that have addressed the quantification of income segregation, its impact on human mobility remains unclear. This study introduces a segregation visitation index and a mobility prediction model to reveal biased travel patterns, showing how segregation and mobility reinforce inequalities and guide more inclusive, sustainable urban planning.

Chen et al., 2026, Patterns 7, 101477
March 13, 2026 © 2025 The Authors. Published by Elsevier Inc.
https://doi.org/10.1016/j.patter.2025.101477

CellPress

Patterns

CellPress
OPEN ACCESS

Article

Promoting sustainable human mobility for income segregation mitigation

Yong Chen,[1] Chenlei Liao,[2] Zeen Cai,[2] Wanru Wang,[3] Yingji Xia,[2] Xiqun (Michael) Chen,[2,6,*] Jianjun Wu,[4] and Ziyou Gao[5,*]
[1]School of Information Technology and Artificial Intelligence, Zhejiang University of Finance and Economics, Hangzhou 310018, China
[2]Institute of Intelligent Transportation Systems, College of Civil Engineering and Architecture, Zhejiang University, Hangzhou 310058, China
[3]School of Management, Zhejiang University of Finance and Economics, Hangzhou 310018, China
[4]School of Economics and Management, Dalian University of Technology, Dalian 116024, China
[5]School of Systems Science, Beijing Jiaotong University, Beijing 100044, China
[6]Lead contact
*Correspondence: chenxiqun@zju.edu.cn (X.C.), zygao@bjtu.edu.cn (Z.G.)
https://doi.org/10.1016/j.patter.2025.101477

THE BIGGER PICTURE Urban income segregation arises from uneven access to amenities and opportunities, shaping not only where people live but also how they move through the city. Yet, most research stops at measuring segregation rather than examining how it influences daily mobility, which is central to social interaction, service access, and environmental exposure. Here, we combined large-scale mobility, socioeconomic, and environmental data from major US cities and developed a framework that moves beyond static spatial metrics. We categorized neighborhoods by segregation level, quantified their mobility tendencies through a simple segregation visitation index, and constructed a segregation-constrained mobility model that embeds structural limitations into travel prediction. Together, these tools allow us to isolate behavioral differences that distance, population, and urban scale alone cannot explain.

Our analyses reveal a consistent and previously overlooked pattern: residents from highly segregated neighborhoods systematically travel toward less segregated areas, reflecting gaps in access to essential services and becoming more pronounced in larger, more urbanized cities. These mobility patterns affect daily experiences—for example, by increasing air pollution exposure—demonstrating that segregation shapes inequality not only through where people live but also through how they move. By integrating segregation, mobility, and environmental conditions, our framework provides actionable insights for urban planning, including targeted improvements to public transit, facility distribution, and neighborhood amenities. Understanding these mobility-driven disparities can help cities reduce inequality, enhance community well-being, and foster inclusive, resilient, and sustainable urban development over time.

SUMMARY

Unraveling urban income segregation fosters social cohesion, urban sustainability, and equitable access to public resources and opportunities for all socioeconomic groups. Here, we show that locations with different segregation levels exhibit biased collective mobility patterns, tending to visit locations with lower segregation levels, which escalate with city size and infrastructure accessibility, and cannot be explained solely by distance and population. Using 1.4 million data points on human mobility, socioeconomic factors, and environmental pollution from 16,093 census tracts in 10 large US cities, we introduce the segregation visitation index to quantify this tendency and develop a human mobility model incorporating segregation constraints and a transfer ensemble optimization component, providing a structural interpretation for the discovered biased mobility. Our results reveal the intricate interplays among urban income segregation, mobility, and environmental exposure, emphasizing the importance of accounting for location-specific mobility differences in developing sustainable income segregation mitigation strategies.

INTRODUCTION

Urban inequality is a complex and widespread social phenomenon, with income,[1] health,[2] and employment[3] inequalities being its primary manifestations. Due to the uneven distribution of social services and opportunities within large cities, various income groups experience unequal access to social resources. Wealthier groups typically have better access to healthcare

© 2025 The Authors. Published by Elsevier Inc.
This is an open access article under the CC BY license (http://creativecommons.org/licenses/by/4.0/).

services and educational resources and have more transportation options.[4] These disparities lead to severe income segregation, visible in the concentration and separation of activity spaces for distinct social groups within the city.[5] Income segregation, in turn, exacerbates urban inequality even further. Therefore, as the central force undermining urban social cohesion, it is essential to understand the income segregation induced by factors such as income, land use, air quality, and the built environment.

Over the past few decades, substantial efforts have been made to quantify income segregation. Due to the challenge of accurately characterizing travelers' physical exposure, researchers generally quantify segregation by approximating the social interactions of various social groups within an area (e.g., neighborhoods and census tracts).[6,7] For example, residential income segregation is measured by the degree to which different social groups are segregated within a place of residence.[8,9] Nevertheless, most social interactions occur outside residential areas,[10–13] where people engage in recreation or work. As mobility data, such as mobile phone signaling data and location-based service data, have become increasingly available, research on quantifying segregation has evolved from focusing on residential income segregation[6–8] to encompassing more dynamic segregation[10–15] experienced in human travels. This type of segregation is no longer solely induced by a city's geographical boundaries; it influences where people go, what services they access, and who they encounter. Therefore, analyzing human travel behavior is a crucial means of understanding the dynamics of income segregation in large cities.

Understanding human travel behaviors has long been a hotspot in transportation research, urban studies, geography, and economics.[16] Previous research has unveiled universal patterns and laws of human travel using diverse transportation data.[17–20] Both individual-level (e.g., the exploration and preferential return [EPR] model[21] and container model[22]) and population-level (e.g., the gravity model [GM][23] and radiation model[24]) models have been developed to reproduce and depict these patterns. Empirical studies have shown correlations between human travel behavior and socioeconomic dynamics,[25] indicating that individuals often engage in activities in places aligned with their socioeconomic categories and interact with individuals of similar socioeconomic backgrounds. Further, some studies have measured the degree of segregation experienced in various locations based on travel data,[6,12,26] comparing the travel patterns of different income groups to illustrate how mobility shapes income segregation.[15,26] Conversely, income segregation constrains local collective mobility patterns, which in turn encode internal drivers of segregation. For example, people in locations with different segregation levels exhibit different complementary needs for services due to unequal access to urban facilities. However, prior research has primarily focused on describing the organizational structure of urban mobility[27,28] or quantifying the degree of spatial segregation across locations,[12,26,29] without delving into how people living in differently segregated areas actually move and behave within the urban system. In particular, it remains unclear whether and how travel scales or collective mobility structures differ across segregation levels and what consequences such disparities entail. These variations are not merely statistical—they may translate into tangible sustainability challenges, such as longer travel distances and heightened mobility burdens for disadvantaged groups, and increased exposure to air pollution. Therefore, gaining a more thorough understanding of segregation-constrained collective mobility patterns can aid in analyzing the intrinsic factors that shape income segregation and facilitate tailored sustainable measures to reduce regional disparities in resource allocation.

To bridge these gaps, we propose a general framework to elucidate how different degrees of income segregation constrain human travel behaviors between locations within large cities. First, we couple mobility data and socioeconomic data from 10 large US cities to calculate income segregation values for all locations and employ a parameter-free method to categorize segregation levels. Second, we analyze the scaling laws of human mobility across locations with different segregation levels and introduce the segregation visitation index (SVI) to explore and quantify the tendency of collective human flows in these locations under segregation constraints. The SVI allows us to establish connections with urban indicators, including city size, infrastructure accessibility, and pollutant emissions. Third, by examining the predictability and transferability of human mobility in locations with different segregation levels, we develop a segregation-constrained human mobility (SCHM) model, which embeds a transfer ensemble algorithm to estimate mobility flows between locations. The proposed model can capture the scaling laws of human mobility at individual and population levels while depicting segregation-constrained visitation patterns. Our model is general and outperforms baseline models across 10 large cities. Our study offers a perspective on exploring the interplay among income segregation, travel behaviors, and other complex variables, providing important implications for sustainable urban management, policy guidance, and mitigating urban inequality.

RESULTS

Income segregation level

To uncover collective mobility patterns among locations with different levels of income segregation, we use 7.1 million anonymized user check-in mobility data (see methods for details) to quantify income segregation across locations (i.e., census tracts) within 10 large cities (i.e., combined statistical areas [CSAs]) in the US (Figure 1A). To ensure both data completeness and representativeness, 10 CSAs with extensive mobility data coverage and long-term socioeconomic statistics were selected. These 10 CSAs have different populations and city sizes and encompass a wide range of historical and spatial contexts—ranging from early industrial and post-industrial centers with rich historical legacies (e.g., Philadelphia and Boston) to global innovation and financial hubs (e.g., New York and San Francisco) and rapidly expanding metropolitan regions undergoing economic diversification and demographic transformation (e.g., Atlanta and Minneapolis). This selection captures the heterogeneity of urban evolution, economic structures, and spatial organization across the US. It is worth noting that our study is applicable to cities with larger or smaller sizes. These 10 CSAs include New York-Newark (NY), Los Angeles-Long Beach (GLA), Washington-Baltimore-Arlington (WB), San Jose-San Francisco-Oakland (SFB), Boston-Worcester-Providence (GB), Philadelphia-Reading-Camden

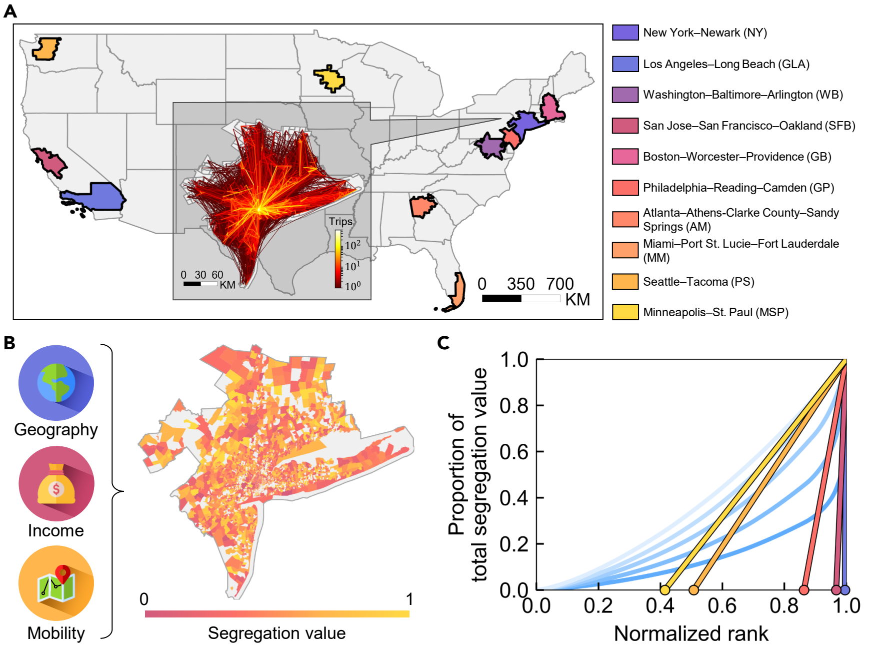

Figure 1. Categorization of income segregation levels

(A) Geographical distribution of 10 large cities (combined statistical area [CSA]). The inset illustration represents the human flow network of all individuals within the New York-Newark CSA observed in the dataset. Line color indicates the number of trips between locations. Brighter colors indicate greater mobility flow, and vice versa.

(B) Distribution of income segregation values experienced in all locations within the New York-Newark CSA. The color of each census tract indicates its corresponding segregation value. Income data are used to classify socioeconomic levels, and mobility data are used to determine the composition of income groups visiting each location.

(C) Division of income segregation levels based on the Lorenz curve. The horizontal axis represents the normalized ranking of each location in ascending order of segregation value. The vertical axis represents the proportion of the cumulative segregation value to the total segregation value. A parameter-free method[27,31] (see methods for details) is adopted to determine categorization thresholds for segregation levels adaptively.

(GP), Atlanta-Athens-Clarke County-Sandy Springs (AM), Miami-Port St. Lucie-Fort Lauderdale (MM), Seattle-Tacoma (PS), and Minneapolis-St. Paul (MSP). Following established approaches,[30] we infer each individual's home location by examining the types of locations they checked in and identifying the most frequently visited locations between 9:00 p.m. and 6:00 a.m. We employ the per-capita income at that location as a proxy for the individual's socioeconomic status (see methods for details). Meanwhile, all individuals within each CSA are divided into four quantiles. Based on the mobility data of all individuals, we quantify the degree of income segregation in each location by measuring the income diversity of its visitors[12] (see Note S1 for details). The segregation value ranges from 0 (no segregation) to 1 (complete segregation), indicating whether a place is frequented by individuals from a single or diverse set of income groups. As an illustration, Figure 1B shows the segregation distribution within the NY CSA. Meanwhile, as shown in Figure S8, the selected 10 cities exhibit distinct degrees of income segregation: while regions within the NY and SFB CSAs show relatively high segregation values, most areas in the GLA, WB, GP, and AM CSAs display lower segregation values. This variability enables the capture of collective mobility patterns across contrasting segregation contexts, ensuring that our findings and the proposed model can be applied to both highly segregated and relatively integrated urban settings. Unlike residential segregation, the activity-based income segregation shows more continuous spatial variation and less pronounced block clustering.[8] Our results show that the calculated segregation value distribution is robust to different quantile definitions, income group selections, and segregation metrics (see Notes S2 and S3 and Tables S2–S4).

Further, in the NY CSA, Spearman correlations between the segregation value of each location and both mobility flow and travel degree are -0.31 ($p \ll 0.05$) and -0.39 ($p \ll 0.05$), respectively (see Figure S9). This indicates that highly segregated locations tend to attract fewer visitors and exhibit lower travel diversity—that is, individuals visit a smaller variety of destinations. Similar robust statistical results are observed across other CSAs (see Table S7 for details). Interestingly, the segregation value of a location is only weakly correlated with its average local income (e.g., Pearson correlation coefficient of 0.35, $p \ll 0.05$, in the NY CSA), suggesting that segregation arises from more intricate behavioral and spatial dynamics rather than income alone.

To further categorize locations by their segregation level, we adapt a parameter-free method based on the Lorenz curve[27,31] (see Figure 1C). Locations are first ranked in ascending order based on their segregation values to construct a cumulative distribution curve of income segregation (i.e., Lorenz curve) within each city. The steepness of various points on the curve depicts the concentration of segregation values. Consequently, the derivative at the point (1, 1) of the Lorenz curve is determined and extrapolated to the intersection with the x axis to establish the threshold for segregation-level categorization. Then, all locations with segregation values greater than this threshold are removed, and the Lorenz curve is regenerated. This process is repeated to categorize all locations into respective segregation levels (see methods for details).

Differences in travel scales of collective human flows

To examine how collective mobility patterns vary across groups in locations with different segregation levels, we construct sub-networks of the citywide human flow network based on the segregation level of departure locations. For example, sub-network 1 includes all trips originating from locations with segregation level 1. For each sub-network, we measure the distributions of travel degree D and travel entropy E, which quantify the number and diversity of destinations reached from a given location, respectively (as shown in Figures 2A and 2B; see Note S3 and Figures S10 and S11 for details). Our analysis

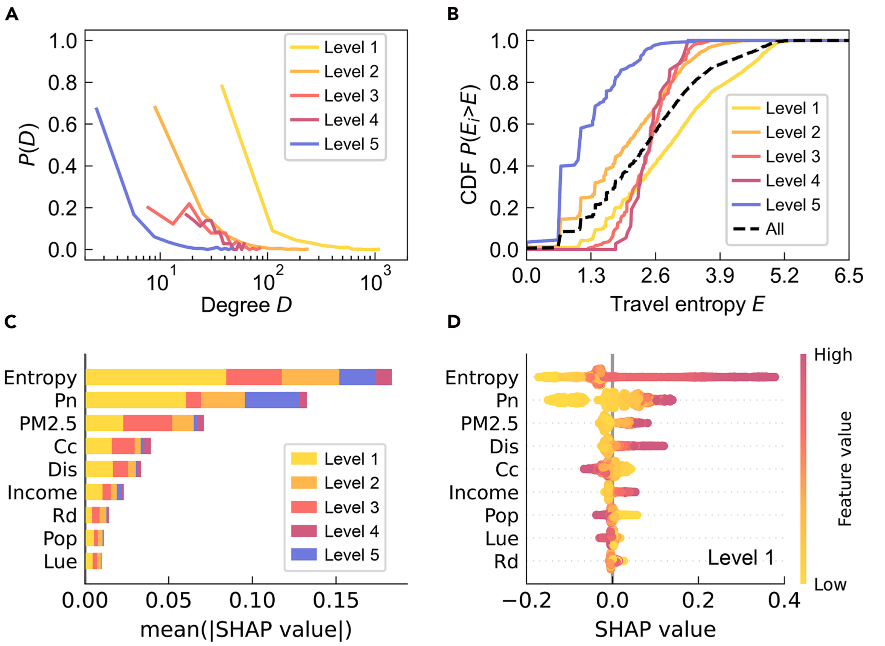

Figure 2. Heterogeneous collective mobility patterns across different segregation levels

(A) Travel degree distribution of locations across different segregation levels.

(B) Travel entropy distribution of locations across different segregation levels. The black dashed line represents the travel entropy distribution for all locations.

(C) Comparison of the relative importance of features across segregation levels, with longer bars indicating greater significance. Pn, Pop, Rd, Dis, Cc, Lue, and PM2.5 denote the number of POI categories, population size, road density, travel distance, clustering coefficient, land-use entropy, and local annual PM2.5 emissions, respectively.

(D) Distribution of SHAP values of all features within a single segregation level. Each scatter point represents a location, with color indicating the feature value. Points positioned to the right of the x axis signify a positive impact on model output, and vice versa.

reveals clear differences in travel scales across segregation levels. Locations with lower segregation (e.g., level 1) exhibit broader and more diversified travel behaviors, reflected in higher values of travel degree and entropy. In contrast, highly segregated locations are associated with narrower and more localized mobility patterns. This trend is consistent across other mobility indicators, such as average travel distance and travel clustering coefficient (Cc) (see Note S5 and Figures S12 and S13 for details). Average travel distance reflects the spatial extent of movement, while Cc captures the transitivity of origin-based flows, indicating how tightly clustered individuals' travel patterns are. Interestingly, locations with moderate segregation levels (e.g., levels 2 and 3) exhibit mixed mobility characteristics, acting as bridges that connect high- and low-income groups. This finding aligns with previous empirical studies[32] and suggests that promoting inclusive land use and mixed-purpose development in such transitional areas may enhance social integration and reduce long-distance commuting.

Furthermore, we use the above mobility measures along with additional urban indicators as input to construct a segregation level classification model based on extreme gradient boosting[33] (XGBoost) and evaluate the impact of each variable using Shapley additive explanations[34] (SHAP) (see Note S6 for details). XGBoost identifies the most informative predictors of segregation level, while SHAP quantifies each feature's marginal contribution to classification outcomes. Together, these analyses reveal which urban mobility and spatial characteristics most strongly influence segregation patterns, offering data-driven insights for designing policies that foster mobility equity and sustainable urban development. Figure 2C shows the relative importance of different variables across segregation levels, while Figure 2D illustrates their SHAP values at level 1 (see Figure S14 for further details). Among them, the number of points of interest (POI) categories (Pn) and land-use entropy (Lue) signify the service diversity of a location, road density (Rd) reflects the transportation convenience around a location, and the total annual emissions of particulate matter (PM2.5) measures the air quality of a location. We find that the relative impact of different variables varies among segregation levels. Specifically, at level 5, travel is predominantly influenced by Pn and has a lesser dependence on Rd. Locations (e.g., industrial parks) with higher segregation levels exhibit reduced service diversity and are typically frequented by minority groups addressing specific needs, potentially showing less concern for accessibility. Conversely, at level 1, a more convenient transportation infrastructure has a greater impact, encouraging a broader range of travel activities. Here, higher Rd supports greater travel variety, emphasizing the importance of investing in multimodal and sustainable transport networks to maintain inclusive accessibility. Notably, these low-segregation zones are also associated with higher PM2.5 emissions, indicating that greater mobility diversity may come at the cost of increased environmental exposure—a trade-off relevant for urban sustainability planning.

SVI

To examine how income segregation shapes collective mobility tendencies, we construct a segregation-constrained visitation matrix SV that quantifies trip flows between locations of different segregation levels (see Figure 3A). Each matrix element represents the normalized probability of traveling from an origin level to a destination level. Our findings reveal that, aside from visiting locations with similar segregation levels, groups in locations at each segregation level consistently exhibit a downward visitation tendency (see Figure S15 for details), i.e., they prefer to visit locations with a lower segregation level. As shown in Figure 3C, we visualize the frequency distribution of POI visits from locations with segregation level 5 to locations with other segregation levels. Locations with low segregation levels better cater to needs such as dining and office spaces (e.g., "food & restaurant"

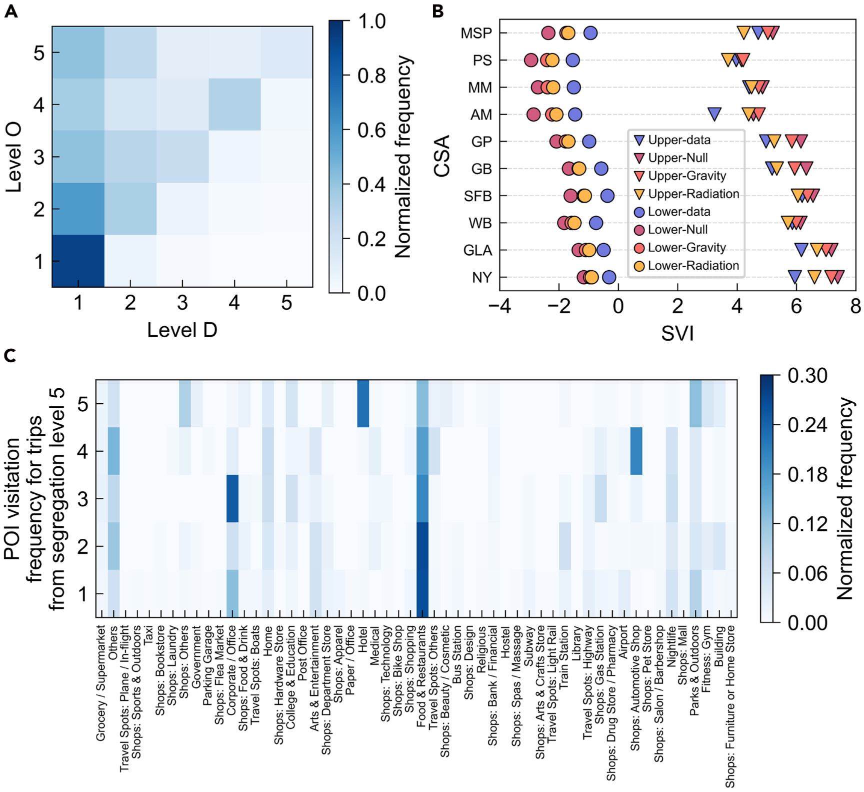

Figure 3. Segregation-constrained collective human flow patterns

(A) Segregation-constrained visitation matrix for the New York-Newark CSA. Color indicates the visitation frequency between each segregation level, normalized based on the total number of trips from the origin level.

(B) Comparison of observed SVI with three reference models across 10 large cities. "Upper" and "lower" represent downward and upward visitation tendencies, respectively. "Null," "gravity," and "radiation" represent the mobility flows generated based on a null model, a gravity model, and a radiation model, respectively (see Note S7 for details). The vertical axis displays the abbreviations of the 10 CSAs (see Table S1 for more information).

(C) Distribution of POI visitation frequencies for trips from locations with segregation level 5 to other locations in the New York-Newark CSA. Color indicates the visit frequencies between each segregation level, normalized based on the total number of trips to the destination level.

and "corporate/office" POIs) and offer a wider range of leisure and entertainment services (e.g., "nightlife" and "arts & entertainment" POIs). Comparisons of POI diversity between origin and destination locations (see Figure S18) confirm that destinations generally offer a broader range of facilities and services. This suggests a potential association between higher segregation levels and reduced diversity or adequacy of local facilities. Groups in high-segregation locations tend to travel to destinations with more diverse services, potentially reflecting compensatory behavior in response to limited local service availability. These results highlight the value of expanding service diversity and improving local amenities in high-segregation locations, which could reduce long-distance travel and promote more equitable access to urban opportunities. It should be noted that these downward visits do not necessarily indicate higher consumption intensity; rather, they often involve routine activities such as commuting to workplaces, schools, or major public facilities. In contrast, due to the relative completeness of infrastructure in low-segregation locations, groups there show a lower tendency to travel to high-segregation locations, except when visiting essential places such as workplaces, schools, and transportation stations (see Figures S16 and S17 for details). Meanwhile, individuals from medium-segregation locations display more diversified mobility patterns, consistent with their mixed land-use context. Such locations function as bridging spaces, simultaneously receiving inflows from higher-segregation locations and sending flows toward more integrated locations. Leveraging this bridging role—through targeted planning to enhance transport accessibility and service complementarity—could strengthen spatial and social integration across the urban landscape. It is essential to emphasize that we do not consider income segregation a direct indicator of facility availability. Instead, we examine the distribution of visits to different types of POIs across segregation levels to explore the potential relationship between income segregation and access to urban services.

We further propose the SVI (see methods for details) to quantify the directional tendency of collective movements across different segregation levels. Let the discrete segregation levels be indexed by 1, …, L, where larger indices correspond to higher segregation levels. Based on the segregation-constrained visitation matrix SV, the SVI is defined as

$$\mathrm{SVI} = \sum_{l_i, l_j = 1}^{L} si_{l_i, l_j} \cdot (l_i - l_j) \qquad \text{(Equation 1)}$$

where si_{l_i,l_j} denotes the normalized visitation frequency with which a group starting from segregation level l_i visits segregation level l_j. $(l_i - l_j)$ represents the span between two segregation levels. The value of SVI reflects the overall directionality of visitation tendencies within the city. If the diagonal elements of matrix SV are one, the SVI value equals zero (i.e., individuals of all levels exclusively visit locations of their level). A positive SVI indicates a downward visitation tendency (i.e., a preference for visiting locations with a lower segregation level than one's own), while a negative value indicates an upward visitation tendency. When only one direction is considered (i.e., $l_i < l_j$ or $l_i > l_j$), we obtain the lower SVI and the upper SVI, respectively. To assess whether the observed tendencies arise from biased visits among groups in locations with different segregation levels and whether they can be replicated by common spatial interaction patterns, we construct three reference models (i.e., null-based, gravity-based, and radiation-based models) with distinct movement constraints for comparative

analysis. The null-based model assumes random travel with equal probabilities, the gravity-based model incorporates distance decay, and the radiation-based model integrates intervening opportunities[24] (e.g., population size) (see Note S7 for details). Figure 3B presents a comparative analysis between empirical SVI values and those generated by the reference models, with the SVI decomposed into upper and lower components whose sum represents the total SVI. We further compare observed SVI values against those from the null-based model to reveal deviations between empirical human flows, physically modeled flows, and random mobility patterns (see Figure S19). The results show that none of the reference models successfully replicates the empirical SVI patterns. All models tend to overestimate upward visitation tendencies and either over- or underestimate downward tendencies to varying degrees. Although the gravity and radiation models consider distance and population effects, they inherently assume symmetric movement potentials between locations and therefore fail to capture the socio-spatial asymmetry embedded in real mobility. In contrast, empirical mobility exhibits persistent directional biases across segregation levels—biases cannot be explained by spatial frictions alone. These results underscore that collective mobility patterns are not merely a function of distance or opportunity structures but are systematically shaped by income-based socio-spatial inequalities. Furthermore, by incorporating alternative segregation metrics and performing uncertainty estimation analyses (see Note S4, Tables S5 and S6, and Figures S20–S22), we validate the robustness of the proposed SVI formulation.

Furthermore, human mobility is shaped by the spatial distributions of urban infrastructure and population. To explore potential associations between segregation visitation patterns and broader urban characteristics, we examine correlations between the SVI and several urban indicators, including population size, urban area, average Rd, and pollutant emissions (i.e., PM2.5, SO_2, and NO_x). These indicators are closely linked to decision-making for sustainable urban governance and public health management.[35,36] As shown in Figures 4A–4F, the SVI exhibits significant positive correlations with these indicators ($p < 0.05$). Please refer to Figures S20–S22 for more results on uncertainty estimation analysis. For example, larger cities with more extensive transportation infrastructures (e.g., New York and GLA) tend to facilitate greater downward visitation tendencies—individuals from highly segregated locations more frequently visit less segregated destinations. In such cities, well-developed transit systems expand the accessible spatial range, thereby increasing the likelihood of cross-segregation travel toward lower segregation levels. In contrast, smaller cities (e.g., PS and AM) typically feature more balanced land and housing accessibility and lower economic concentration, leading to weaker downward visitation tendencies. This implies that, in large cities, improving connectivity and transportation accessibility within highly segregated areas can promote equitable mobility, while in smaller cities, enhancing local service diversity and targeted amenities may be more effective than expanding transit infrastructure. Figures 4D–4F further illustrate the relationship between the SVI and the average annual total air pollutant emissions (tons per year) at travel destinations within each CSA. We find that higher SVI values are associated with increased exposure to air pollution during travel, as destinations of downward visits tend to exhibit higher pollutant emissions. Industrialized and densely urbanized cities (e.g., NY and GB) show particularly elevated exposure risks, reflecting the compounding effects of long-distance mobility constrained by segregation. To further assess emission disparities across segregation levels, we calculate the relative differences in pollutant emissions for trips to destinations with the same (same), higher (upward), and lower (downward) segregation levels as the origin. As demonstrated in Figures 4G–4I, downward trips consistently exhibit greater pollution burdens at destinations, whereas upward trips experience notably lower emission levels; same-level trips show minimal differences. These findings indicate that reducing cross-segregation travel—for instance, by increasing local amenities in high-segregation neighborhoods or promoting low-emission mobility modes—can yield dual benefits: enhancing mobility equity and advancing environmental sustainability.

SCHM prediction

To accurately reproduce and characterize mobility patterns under income segregation constraints, we develop an SCHM model (see Figure 5). The model comprises location exploration and return by jointly considering location attractiveness, individual memory, and segregation constraints. For each individual u, he/she decides with probability α whether to engage in travel activities subject to memory constraints. When the individual opts to return to a previously visited location, the probability of moving from location i to location j is $p_{ij} \propto te_j \cdot m_j^u \cdot sv_{l_i,l_j}$. Location attractiveness te_j reflects the popularity or pull of a destination—the higher its attractiveness, the greater the likelihood of visitation. Historical memory m_j^u encodes individual preferences for previously visited locations. In this study, m_j^u is defined by the historical visitation frequency of individual u to each location. te_j is characterized by the sum of the travel probabilities from other locations to the target location. In this study, we introduce a transfer ensemble model to enhance the estimation accuracy of the travel distribution between locations. In addition, an individual's travel choice is also constrained by the visitation probability between different segregation levels (i.e., sv_{l_i,l_j}), ensuring that travel decisions follow empirically observed directional biases between segregation levels (e.g., downward or upward visits). The probability sv_{l_i,l_j} is derived from the segregation-constrained visitation matrix SV. In the absence of historical memory constraints, travel choice simplifies to $p_{ij} \propto te_j \cdot sv_{l_i,l_j}$, indicating that mobility is jointly determined by destination attractiveness and segregation-level visitation tendencies.

For our proposed transfer ensemble model, we first observe variations in the predictability of collective human flows across segregation levels using origin-constrained GMs[16] (Figure 6A). The root-mean-square error (RMSE) decreases as the segregation level increases, indicating that mobility patterns in highly segregated locations (e.g., level 5) are more regular and predictable, whereas those in low-segregation areas are more diverse and dynamic. This heterogeneity poses challenges for constructing a single, citywide GM (global GM) capable of accurately capturing mobility across all segregation levels. In contrast, separate models trained for each level (local GM) better fit level-specific dynamics but fail to generalize across the urban system (see Note S8 for model details). To examine whether

CellPress
OPEN ACCESS

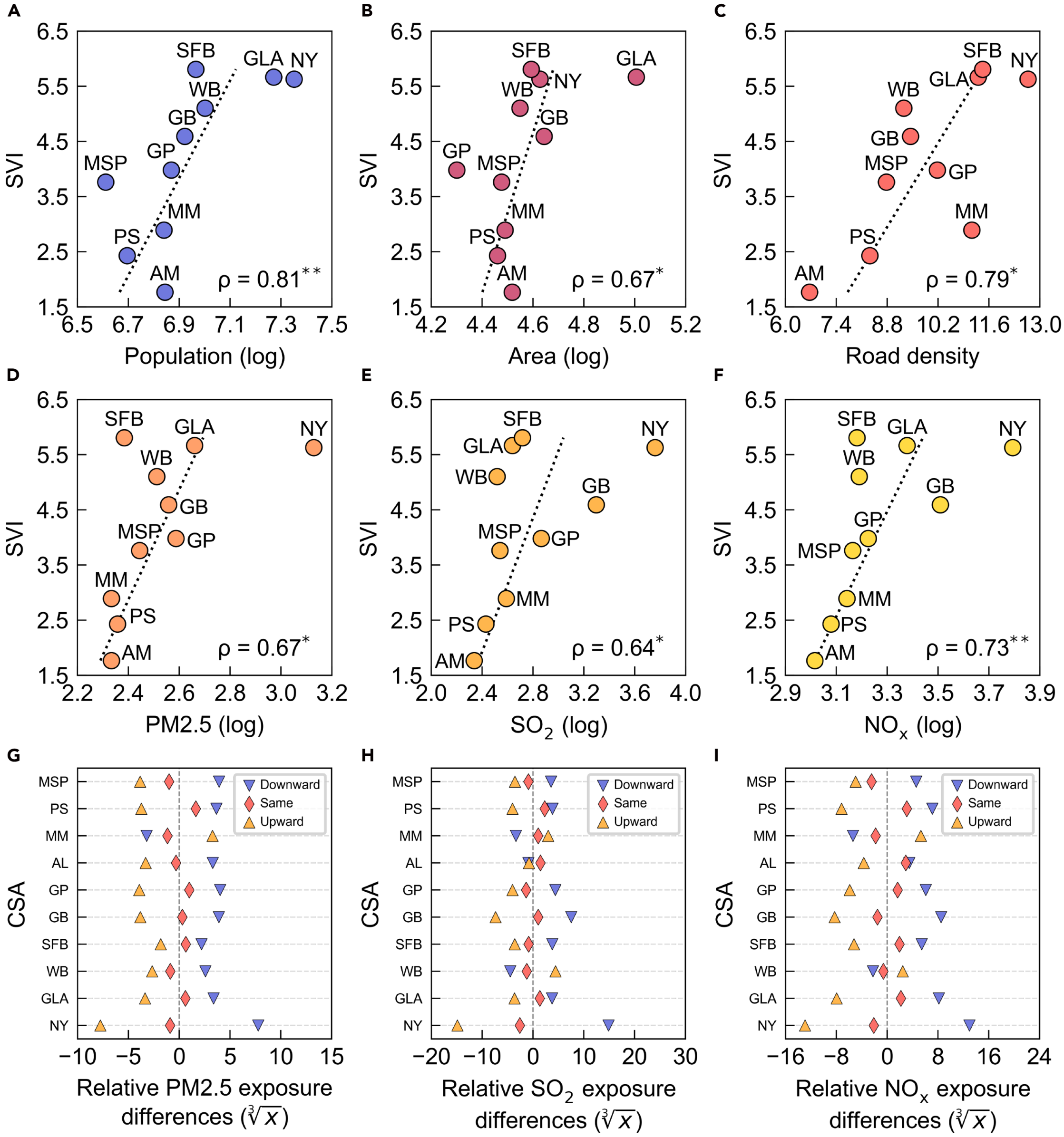

Figure 4. Correlation between SVI and urban indicators

Correlation between SVI and urban indicators: (A) population, (B) area, (C) road density, (D) PM2.5 emissions, (E) SO_2 emissions, and (F) NO_x emissions. Pollutant emissions are the total annual emissions in tons. ρ denotes the Spearman correlation coefficient. $^{**}p < 0.01$ and $^{**}p < 0.05$, respectively.

(G–I) Average relative differences in pollutant emissions between travel origins and destinations across 10 large cities. "Upward," "downward," and "same" represent trips to locations with higher, lower, and the same segregation levels compared to the origin, respectively.

mobility patterns are transferable between cities, we estimate a GM using collective human flow data from locations within a large city at a certain segregation level (i.e., source domain). We then transfer this model to predict human flows for locations in other large cities with the same segregation levels (i.e., target domain) (see methods for details). As shown in Figure 6B, transfer predictions for level-5 flows achieve performance comparable to models trained directly on target-city data, suggesting that human mobility behaviors exhibit cross-city regularities within the same segregation strata (see Figure S23 for details). "CPC" denotes the common part of commuters,[16] measuring the similarity between predicted and actual flows. Building on this observation, we introduce a transfer ensemble model (i.e., ensemble GM) to enhance estimation accuracy beyond traditional gravity-based approaches. Specifically, we train multiple origin-constrained GMs using data from several cities sharing the same segregation level. These models generate a set of transfer-based predictions for the target city, which are aggregated into a feature matrix and fed into an XGBoost meta-learner to produce the final human flow estimation (see methods for details). As shown in Figures 6C and 6D, the proposed ensemble GM consistently outperforms both the local GM and the global

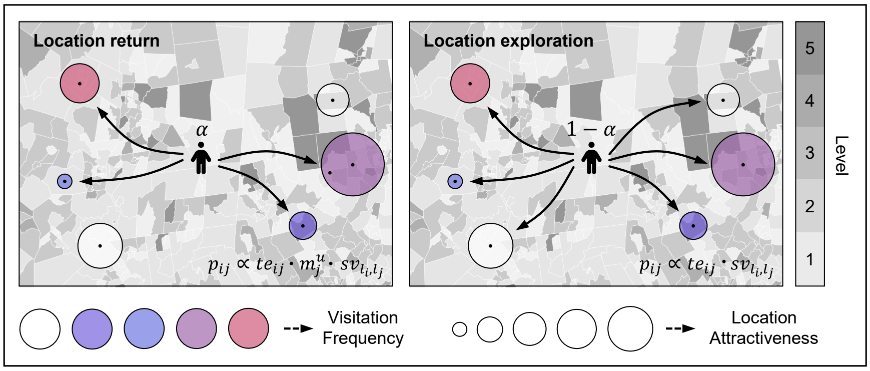

Figure 5. Illustration of a segregation-constrained human mobility model

The individual selects returning to a location with probability α or engaging in location exploration with probability $1 - \alpha$. When individual u is located at location i, he/she will consider memory constraints (i.e., historical visit frequency m_j^u; depicted as the circle's color) and location attractiveness constraints (te_j; depicted as the circle's size) to select a location to visit. Meanwhile, the location selection of the individual will be influenced by the income segregation of the current location, characterized by the visitation frequency (sv_{l_i,l_j}; depicted as color shades on the map) between locations with different segregation levels. The location exploration performs similar steps but does not consider memory constraints.

GM across major cities. In the NY CSA, for example, the estimated trips closely match observed flows, with a Pearson correlation of 0.75 ($p \ll 0.001$) (see Figure S24 for more examples). The transferability of mobility patterns suggests that lessons learned from one city can be applied to others with similar segregation profiles, potentially guiding scalable strategies for improving equitable access to services and mitigating associated environmental impacts, including pollution exposure.

Finally, we utilize a subset of historical mobility data from 10 large cities to fit the SCHM model and generate synthetic travel trajectories for each (see methods for details). The travel sample encompasses 12,937 census tracts across 201 counties in the US. To evaluate the model's accuracy, we compare the generated trajectories against empirical travel data across six key metrics (see Figure 7): the distribution of radius of gyration, the distribution of individuals' location visitation frequency, the distribution of the number of locations visited by individuals, the total number of location visits within t trips, the number of trips T between locations, and the visitation frequency error between locations with different segregation levels. These indicators jointly assess model performance from individual, collective, and segregation perspectives. Please refer to Figures S25–S31 for more performance evaluations. For benchmarking, we introduce the EPR model[21] and its four variant models, i.e., gravity-based EPR (D-EPR),[19] recency-based EPR[37] (R-EPR), memory-based EPR[38] (M-EPR), and social EPR[12] (see Note S9 for details), for performance comparison. All models were run under identical initial conditions and simulation settings, following consistent trajectory-generation procedures and parameter calibrations drawn from prior empirical studies.[19,21,37,38]

As shown in Figure 7, SCHM markedly outperforms all EPR-based baselines (see Table S8). The synthetic trajectories produced by SCHM closely replicate the empirical distributions of key mobility indicators. Compared with EPR-based models, which primarily encode memory- or distance-based returns and

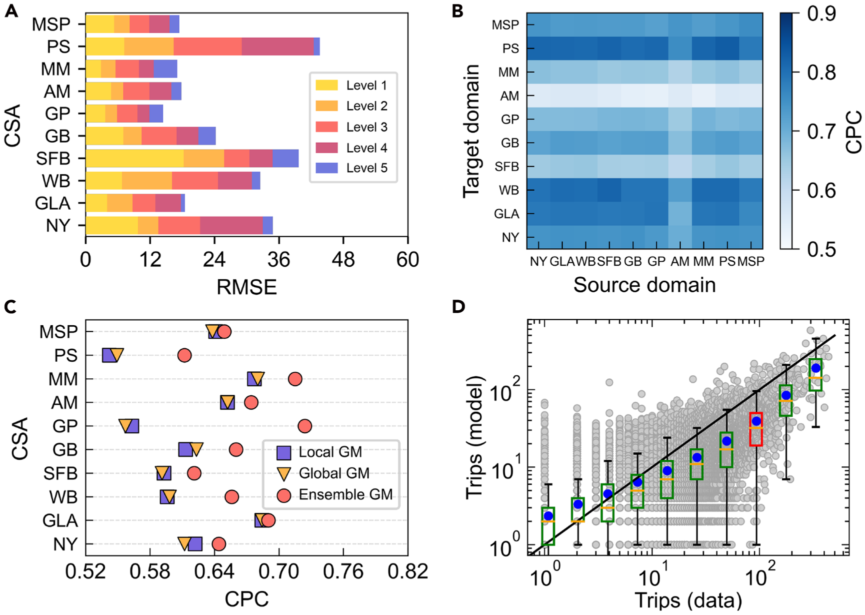

Figure 6. Predictability and transferability analysis of collective human flows in different cities

(A) Comparison of the predictability of collective human flows across segregation levels, with longer bars indicating greater RMSE.

(B) Transfer prediction performance for collective human flows at locations with the same segregation level (level 5) between different large cities.

(C) Performance comparison of the proposed transfer ensemble model and baseline models.

(D) Paired comparisons of predicted and real trips within the New York-Newark CSA. Gray points indicate observed and predicted location pairs. The boxplot illustrates the distribution of predicted trips across various ranges of observed trip counts. The green-shaded box indicates that the diagonal line $y = x$ falls within the 5th and 95th percentiles, and red otherwise. Blue points represent the average predicted trip counts across different bins.

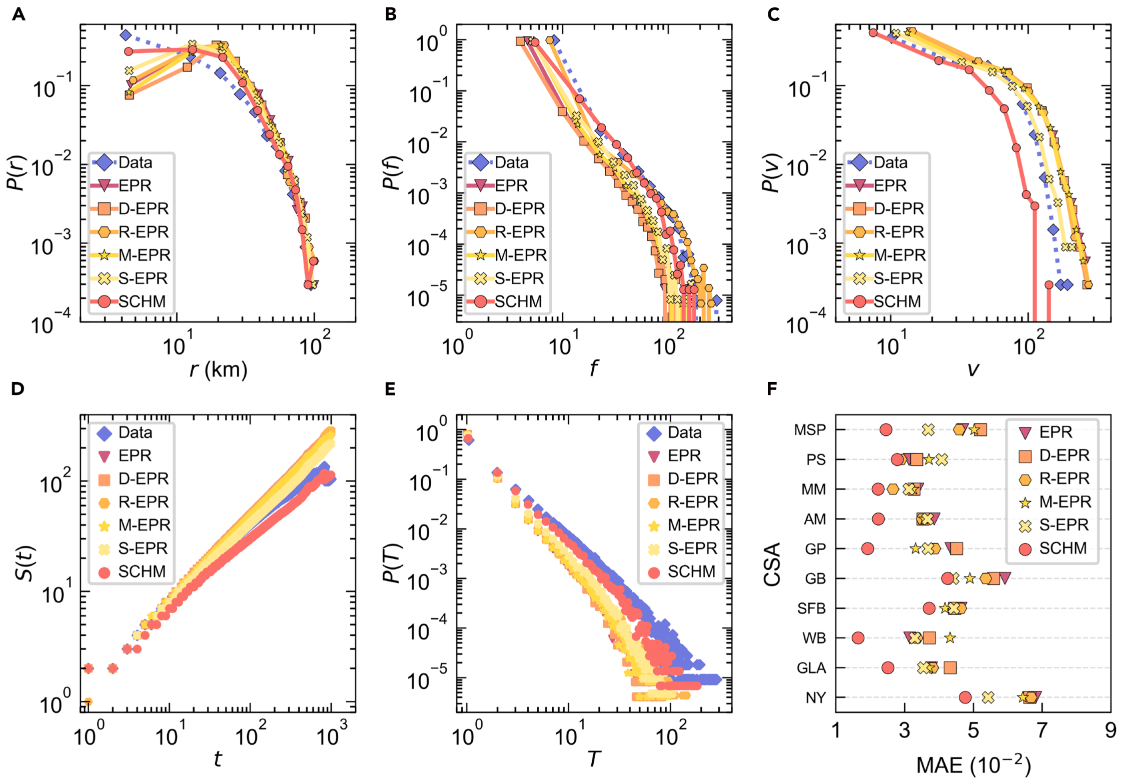

Figure 7. Human mobility prediction performance

(A) Distribution of radius of gyration in the New York-Newark CSA.

(B) Frequency distribution of individuals visiting a location in the New York-Newark CSA.

(C) Distribution of the number of locations visited by individuals in the New York-Newark CSA.

(D) Distribution of the total number of locations visited within t trips in the New York-Newark CSA.

(E) Comparison between predicted and actual distributions of the different number of trips T between two locations in the New York-Newark CSA.

(F) Distribution of prediction errors in visitation probability between locations with different segregation levels for 10 large cities.

exploration strategies, SCHM integrates a transfer ensemble method that leverages cross-city, segregation-level-consistent mobility patterns, substantially enhancing the generalizability and predictive accuracy of gravity-based flow estimation. These design choices enable SCHM to more accurately reproduce empirical scaling laws at individual and population levels while significantly reducing prediction errors in human flows between locations. For instance, at the individual level, the prediction results of SCHM are closer to the empirical distribution than those of baseline models in describing scaling laws, such as the radius of gyration, location visitation frequency, and the number of locations visited. Additionally, SCHM accurately captures the scaling laws of the algebraic increase in the total number of location visits. At the population level (Figure 7E), SCHM achieves superior accuracy in predicting aggregate trips between locations, closely matching the empirical algebraic decay and convergence patterns of inter-location flows. Moreover, SCHM yields the smallest visitation-frequency error between locations with different segregation levels for all large-city datasets (Figure 7F; see Table S9 and Figure S31 for details). Among the EPR-based models, S-EPR performs better by incorporating income-segregation constraints, yet it still falls short of SCHM's performance. These results underscore SCHM's ability to capture the structural heterogeneity of urban mobility by integrating segregation-level-aware visitation constraints—an essential aspect often overlooked by traditional mobility models (e.g., D-EPR and R-EPR). The approach yields more realistic trajectory generation and a more accurate depiction of cross-segregation-level flow dynamics. From a practical standpoint, SCHM offers a scalable and generalizable framework for quantifying how income segregation shapes the scale and direction of urban travel. By explicitly modeling segregation-specific behavioral regularities and their transferability across cities, SCHM enables urban planners and policymakers to diagnose mobility inequities and to simulate systemic impacts of interventions. For example, the model can evaluate how improving transit accessibility in high-segregation neighborhoods, redistributing essential services, or adjusting land-use configurations would alter cross-level mobility flows and overall network efficiency.

DISCUSSION

Urbanization is rapidly accelerating, making urban income segregation an increasingly prominent and pressing issue. This study provides a lens for disentangling the complex interplay between income segregation and human mobility patterns, moving

beyond traditional location-level metrics.[11,12] By categorizing urban locations into different segregation levels, we uncover pronounced heterogeneity in collective mobility patterns among locations experiencing various degrees of income segregation. In particular, locations with moderate segregation serve as crucial "bridging zones" that foster cross-group connectivity between high- and low-income groups. Moreover, we reveal that segregation levels are closely associated with disparities in local service provision, which, in turn, drive biased visitation patterns and reinforce unequal access to urban amenities. A consistent downward visitation trend—where groups from highly segregated areas travel to less segregated zones—is observed across major cities and becomes increasingly pronounced in larger, more infrastructure-intensive urban environments. Notably, this structural mobility bias also leads to disproportionate exposure to air pollution, highlighting a neglected dimension of environmental injustice.

To model these nuanced dynamics, we propose a SCHM model embedded with a transfer ensemble framework. This model not only replicates empirical scaling laws but also offers a robust structural account of how structural inequalities shape behavioral mobility decisions. The integration of segregation-aware constraints enables us to capture the cognitive trade-offs individuals make between accessibility, opportunity, and environmental exposure. From a behavioral perspective, residents in highly segregated locations tend to exhibit compensatory mobility strategies—traveling further to satisfy unmet service or social needs—whereas individuals in less segregated locations display higher spatial inertia due to localized service sufficiency. These behavioral adaptations provide a human-centered interpretation of how structural inequalities manifest in aggregate mobility flows.

Taken together, these findings highlight the importance of incorporating segregation effects into the modeling and planning of sustainable and equitable urban mobility systems. Beyond theoretical contributions, our work carries concrete societal and policy relevance. It directly informs the design of spatially targeted interventions that can alleviate inequality while promoting long-term sustainability. Empirically, we demonstrate that groups from highly segregated locations may compensate for deficiencies in essential services by visiting locations with lower segregation levels. This insight guides urban planners in prioritizing specific service facilities around locations with high segregation levels, such as large convenience supermarkets, outdoor recreational facilities, and public transportation stations. These actions not only reduce travel burdens and carbon emissions but also foster local economic vitality, social cohesion, and community well-being—key dimensions of sustainable and inclusive urban development.

Furthermore, our results reveal a trade-off between urban expansion and intra-city segregation intensity in large cities. In cities with higher urbanization, individuals exhibit a stronger preference for low-segregation destinations, suggesting an imbalance in urban facility distribution. This underscores the need for policymakers to align infrastructure investment and service allocation with equitable development objectives. At the same time, our findings underscore that compensatory long-distance travel may inadvertently increase environmental and health risks[39] through greater air pollution exposure. Addressing this dual challenge requires integrative policy design—combining service accessibility improvement with targeted pollution mitigation. For example, implementing stricter emission controls in high-traffic bridging zones or expanding low-emission public transit routes can jointly reduce exposure disparities and enhance environmental justice.

Crucially, these policy insights are operationalizable through our SCHM framework. Our SCHM framework provides a computational platform that allows policymakers to simulate and evaluate the real-world impacts of intervention strategies—such as facility redistribution, zoning reforms, and emission controls—on both mobility behavior and social equity outcomes. By incorporating segregation-level-aware visitation constraints and a transfer-based ensemble learning mechanism, SCHM facilitates fine-grained "what-if" analyses, empowering urban planners to design strategies that are evidence based, equitable, and sustainability oriented. In this sense, our model functions as a decision-support tool that translates behavioral insights into operational policy guidance.

In contrast to existing location-based or individual-based segregation measures, this study focuses on uncovering variations in collective mobility patterns among groups in locations across different levels of segregation and provides actionable insights into mitigating income segregation and promoting sustainable urban development. Nevertheless, several limitations should be acknowledged. First, our analysis uses income-based segregation as a proxy for broader urban segregation, potentially overlooking other dimensions such as race, education, or occupation. While our framework is extensible, future work could benefit from a multidimensional segregation characterization. Second, our mobility analysis relies on check-in data, which may not fully represent contemporary urban mobility behaviors or demographic conditions. Nevertheless, the validity of our conclusions primarily rests on the structural and behavioral patterns uncovered rather than the specific temporal context of the data. The fundamental relationships identified—such as the compensatory visitation behaviors among groups in highly segregated areas and the bridging role of moderately segregated zones—reflect enduring spatial and socioeconomic dynamics that are likely relevant across periods. However, the generalizability of the empirical results to present-day urban contexts should be interpreted with caution, as the magnitude of segregation-mobility interactions may shift with evolving spatial configurations and socioeconomic conditions. To further enhance validity and generalizability, future research should recalibrate and validate the SCHM framework using more recent and diversified mobility datasets—such as mobile phone signaling or transit smart-card records—that better represent population heterogeneity and trip purposes. Moreover, longitudinal analyses could help assess the temporal persistence of the observed patterns and disentangle structural regularities from context-dependent effects. Third, socioeconomic attributes are estimated at the location level rather than the individual level due to privacy constraints. Future studies could improve granularity by integrating additional data sources, such as anonymized income, housing, or consumption data. Finally, although SCHM supports policy simulation under hypothetical scenarios, it assumes static segregation during simulations. Future extensions could integrate feedback mechanisms between mobility behaviors, segregation dynamics, and

CellPress
OPEN ACCESS

policy responses to better reflect the evolving nature of urban systems.

METHODS

Ethics and inclusion statement

The research did not include local researchers. The roles and responsibilities were agreed upon among collaborators ahead of the research. This research did not include biological materials, animals, or human subjects, for whom ethical approval is required.

Dataset

We leverage mobility data derived from user check-ins gathered on the Weeplaces[40] website, which integrates location-based check-in information from various service platforms (e.g., Facebook and Gowalla). This dataset is a collated and anonymized dataset containing 7,164,507 travel records (i.e., check-ins) of 15,790 users spanning January 2010 to June 2011. Each record includes the anonymous user's identifier, travel time, travel location (including location's identifier, longitude, and latitude), and activity type. This study uses census tracts as the fundamental unit of travel analysis. The movement of an individual from one location to another is considered a trip (or a new location visit), regardless of whether the destination is their place of residence. The maximum interval between an activity being considered as a trip was 24 h (see Figure S1). For consecutive records in the same census tract, we retained only one trip; that is, intra-zonal trips were not considered. At the same time, we also removed individuals with fewer than 8 trips to reduce random bias. Based on the activity types of all users' historical travel, we can count the number of POI categories in a census tract, which measures the service diversity of a location. Finally, we exclusively consider trips occurring within 10 large cities (i.e., CSAs), which cover 16,093 census tracts across 201 US counties (see Figure 1A; Table S1) and contain 1,453,936 trips for 10,524 individuals. The distribution of trips of all individuals is shown in Figure S2. The distribution of the number of visits to each census tract (including residents and non-residents) is shown in Figure S3. Since some places were visited by fewer people, we excluded census tracts that were visited fewer than 30 times to reduce the bias of the results. A CSA comprises multiple metropolitan areas where individuals often participate in economically, politically, and culturally relevant spatial interactions. We employ a standardized procedure[30] to infer each individual's home location by considering the location category of the individual's check-ins and the most frequently visited location between 9:00 p.m. and 6:00 a.m. Further, we use the per-capita income of the census tract, where the home is situated as a proxy,[10,41] for the income level of the target individual. We follow the established pipeline[26] to verify that the inferred individual income levels within each CSA accurately reflect the population distribution (see Note S2 and Figure S4). Meanwhile, the income of all individuals within a large city is divided into four equal-sized quantile intervals. Each individual is classified into groups of different economic levels: low income, low-middle income, higher-middle income, and high income. All administrative boundary data and socioeconomic data (i.e., income and population) are obtained from the official website of the US Census Bureau.[42] Moreover, urban indicator data related to the city, including urban area, road network density, and land-use data, are gathered from the OpenStreetMap[43] database. Air pollutant emission data are collected from the global atmospheric research emission database (EDGAR).[44] Meanwhile, Lue is calculated using the information entropy method.[45] EDGAR provides a gridded annual total of pollutant emissions, which we project to each census tract based on the grid center coordinates (see Figures S5–S7).

Segregation-level categorization

The Lorenz curve is widely utilized for calculating the Gini coefficient, which measures the equality of income distribution in countries and regions.[46] Meanwhile, the slopes of the curve also contain valuable information. This study adapts the LouBar method[27,31] to adaptively establish thresholds for categorizing different levels of income segregation.

We first rank the segregation values of all locations within a target city in ascending order and subsequently obtain the cumulative distribution curve (i.e., Lorenz curve) of the segregation values of all locations (Figure 1C). The curve's horizontal axis represents the ranking of each location, and the vertical axis represents the proportion of total segregation value. If the probability of occurrence for all segregation values is uniformly distributed, the Lorenz curve will form a straight line from 0, 0 to 1, 1. Since the slopes of different points on the curve indicate the degree of aggregation for segregation values, points with a larger slope have a lower degree of aggregation. Consequently, we take the derivative at the point (1, 1) of the Lorenz curve and extrapolate to the intersection of the x axis to obtain the segregation threshold T_L. We then remove the locations with segregation values greater than or equal to T_L from the curve, generate a new Lorenz curve (light blue curve in Figure 1C), and so forth. Ultimately, all locations are categorized into different segregation levels. To facilitate the comparison of various large cities, we have categorized them into up to five segregation levels.

Transfer ensemble model

Building on the predictability and transferability analysis of different segregation levels in collective human flows across various large cities, we propose a transfer ensemble model to enhance the accuracy of human flow estimation between various locations within large cities. To illustrate, considering the estimation of collective human flows of all locations with the segregation level equal to level 1 in the NY CSA, we utilize travel data (labeled as D_1–D_{10}) from locations with the same segregation level in 10 large cities to estimate 10 GMs, respectively. Meanwhile, we partition the dataset into a training set and a test set with a ratio of 8:2. Subsequently, we employ the estimated 10 GMs to perform transfer predictions on the training dataset of the NY CSA (D_1), resulting in 10 sets of transfer prediction results for D_1. For each data point, we stack 10 prediction results to create a new dataset, denoted as D_{new}. Finally, we use XGBoost[33] as the meta-estimator to establish a stacking-based ensemble prediction model and utilize D_{new} for training and estimating the travel distribution of the NY CSA. Leveraging transfer prediction and ensemble techniques at the same segregation level across large cities, the proposed model significantly enhances the accuracy of collective human flow estimation (see Figures 5C and 5D).

Travel trajectory generation

We model individuals' travel transitions by incorporating constraints across three dimensions: location attractiveness, individual memory, and visitation frequency between different segregation levels. The simulation involves generating individual travel trajectories through the following operations.

(1) Initialize the travel status information. Assume the travel length of individual u in the actual observation dataset is Tl_u. Utilize historical travel data consisting of $\frac{1}{3}Tl_u$ steps to initialize the historical memory vector m^u for the target individual u, representing the frequency of visits to each location. Similarly, calculate the segregation-constrained visitation matrix SV using one-third of the historical travel data of all individuals. Moreover, employ the proposed transfer ensemble model to estimate the travel distribution between various locations and obtain location attractiveness vectors te.
(2) Next, assuming that the individual is currently at location i, based on the proposed human mobility model, the individual chooses a location to explore or return to.
(3) Update the current location of individual u, memory information, and cross-segregation level visitation information.
(4) Repeat steps 2 and 3 to generate a travel trajectory with $\frac{2}{3}Tl_u$ travel steps.

RESOURCE AVAILABILITY

Lead contact

Further information and requests for resources and reagents should be directed to and will be fulfilled by the lead contact, Xiqun (Michael) Chen (chenxiqun@zju.edu.cn).

Materials availability

This study did not generate new unique materials.

Data and code availability

The travel data and other data related to this study are available from Zenodo[47] (https://doi.org/10.5281/zenodo.15314012). National administrative division and socioeconomic data of the US are publicly available at https://www.census.gov/data.html. Road network and regional land-use data are publicly available at https://www.openstreetmap.org. Air pollutant emission data are publicly available at https://edgar.jrc.ec.europa.eu/. The code used for data processing and analysis, segregation calculation, segregation-level categorization, travel pattern analysis, and mobility model building and training is available from Zenodo[48] (https://doi.org/10.5281/zenodo.13742568). The accession number for the code and pre-computed data reported in this paper is Zenodo: https://doi.org/10.5281/zenodo.13742568.

ACKNOWLEDGMENTS

This research is financially supported by the National Natural Science Foundation of China (72288101, 72525009, 72431009, 72171210, and 72350710798) and the Zhejiang Provincial Natural Science Foundation of China (LZ23E080002, LQN26E080004).

AUTHOR CONTRIBUTIONS

Y.C., Z.G., and X.(M.)C. proposed the question. Y.C., C.L., W.W., and Y.X. designed and conducted the experiments. Y.C., Z.C., and X.(M.)C. developed the algorithms. Y.C., J.W., Z.G., and X.(M.)C. wrote the paper.

DECLARATION OF INTERESTS

The authors declare no competing interests.

DECLARATION OF GENERATIVE AI AND AI-ASSISTED TECHNOLOGIES IN THE WRITING PROCESS

The authors declare that no generative AI tools were used in the writing of this manuscript or in the editing of any associated figures.

SUPPLEMENTAL INFORMATION

Supplemental information can be found online at https://doi.org/10.1016/j.patter.2025.101477.

Received: September 5, 2025
Revised: November 7, 2025
Accepted: December 18, 2025
Published: March 2, 2026

REFERENCES

1. Ravallion, M. (2014). Income inequality in the developing world. Science *344*, 851–855. https://doi.org/10.1126/science.1251875.
2. Bor, J., Cohen, G.H., and Galea, S. (2017). Population health in an era of rising income inequality: USA, 1980–2015. Lancet *389*, 1475–1490. https://doi.org/10.1016/S0140-6736(17)30571-8.
3. Calvo-Armengol, A., and Jackson, M.O. (2004). The effects of social networks on employment and inequality. Am. Econ. Rev. *94*, 426–454. https://doi.org/10.1257/0002828041464542.
4. Macedo, M., Lotero, L., Cardillo, A., Menezes, R., and Barbosa, H. (2022). Differences in the spatial landscape of urban mobility: Gender and socioeconomic perspectives. PLoS One *17*, e0260874. https://doi.org/10.1371/journal.pone.0260874.
5. Gambetta, D., Mauro, G., and Pappalardo, L. (2023). Mobility constraints in segregation models. Sci. Rep. *13*, 12087. https://doi.org/10.1038/s41598-023-38519-6.
6. Wang, Q., Phillips, N.E., Small, M.L., and Sampson, R.J. (2018). Urban mobility and neighborhood isolation in America's 50 largest cities. Proc. Natl. Acad. Sci. USA *115*, 7735–7740. https://doi.org/10.1073/pnas.1802537115.
7. Van Ham, M., Uesugi, M., Tammaru, T., Manley, D., and Janssen, H. (2020). Changing occupational structures and residential segregation in New York, London and Tokyo. Nat. Hum. Behav. *4*, 1124–1134. https://doi.org/10.1038/s41562-020-0927-5.
8. Tammaru, T., Marcińczak, S., Aunap, R., van Ham, M., and Janssen, H. (2020). Relationship between income inequality and residential segregation of socioeconomic groups. Reg. Stud. *54*, 450–461. https://doi.org/10.1080/00343404.2018.1540035.
9. Reardon, S.F., and Bischoff, K. (2011). Income inequality and income segregation. Am. J. Sociol. *116*, 1092–1153. https://doi.org/10.1086/657114.
10. Nilforoshan, H., Looi, W., Pierson, E., Villanueva, B., Fishman, N., Chen, Y., Sholar, J., Redbird, B., Grusky, D., and Leskovec, J. (2023). Human mobility networks reveal increased segregation in large cities. Nature *624*, 586–592. https://doi.org/10.1038/s41586-023-06757-3.
11. Athey, S., Ferguson, B., Gentzkow, M., and Schmidt, T. (2021). Estimating experienced racial segregation in US cities using large-scale GPS data. Proc. Natl. Acad. Sci. USA *118*, e2026160118. https://doi.org/10.1073/pnas.2026160118.
12. Moro, E., Calacci, D., Dong, X., and Pentland, A. (2021). Mobility patterns are associated with experienced income segregation in large US cities. Nat. Commun. *12*, 4633. https://doi.org/10.1038/s41467-021-24899-8.
13. Wang, S., Zheng, Y., Wang, G., Yabe, T., Moro, E., and Pentland, A.‘. (2024). Infrequent activities predict economic outcomes in major

American cities. Nat. Cities *1*, 305–314. https://doi.org/10.1038/s44284-024-00051-7.

14. Bonaccorsi, G., Pierri, F., Scotti, F., Flori, A., Manaresi, F., Ceri, S., and Pammolli, F. (2021). Socioeconomic differences and persistent segregation of Italian territories during COVID-19 pandemic. Sci. Rep. *11*, 21174. https://doi.org/10.1038/s41598-021-99548-7.
15. Li, X., Huang, X., Li, D., and Xu, Y. (2022). Aggravated social segregation during the COVID-19 pandemic: Evidence from crowdsourced mobility data in twelve most populated US metropolitan areas. Sust. Cities Soc. *81*, 103869. https://doi.org/10.1016/j.scs.2022.103869.
16. Barbosa, H., Barthelemy, M., Ghoshal, G., James, C.R., Lenormand, M., Louail, T., Menezes, R., Ramasco, J.J., Simini, F., and Tomasini, M. (2018). Human mobility: Models and applications. Phys. Rep. *734*, 1–74. https://doi.org/10.1016/j.physrep.2018.01.001.
17. Brockmann, D., Hufnagel, L., and Geisel, T. (2006). The scaling laws of human travel. Nature *439*, 462–465. https://doi.org/10.1038/nature04292.
18. Gonzalez, M.C., Hidalgo, C.A., and Barabasi, A.L. (2008). Understanding individual human mobility patterns. Nature *453*, 779–782. https://doi.org/10.1038/nature06958.
19. Pappalardo, L., Simini, F., Rinzivillo, S., Pedreschi, D., Giannotti, F., and Barabási, A.L. (2015). Returners and explorers dichotomy in human mobility. Nat. Commun. *6*, 8166. https://doi.org/10.1038/ncomms9166.
20. Schläpfer, M., Dong, L., O'Keeffe, K., Santi, P., Szell, M., Salat, H., Anklesaria, S., Vazifeh, M., Ratti, C., and West, G.B. (2021). The universal visitation law of human mobility. Nature *593*, 522–527. https://doi.org/10.1038/s41586-021-03480-9.
21. Song, C., Koren, T., Wang, P., and Barabási, A.L. (2010). Modelling the scaling properties of human mobility. Nat. Phys. *6*, 818–823. https://doi.org/10.1038/nphys1760.
22. Alessandretti, L., Aslak, U., and Lehmann, S. (2020). The scales of human mobility. Nature *587*, 402–407. https://doi.org/10.1038/s41586-020-2909-1.
23. Zipf, G.K. (1946). The P_1 P_2/D hypothesis: On the intercity movement of persons. Am. Sociol. Rev. *11*, 677–686. https://doi.org/10.2307/2087063.
24. Simini, F., González, M.C., Maritan, A., and Barabási, A.L. (2012). A universal model for mobility and migration patterns. Nature *484*, 96–100. https://doi.org/10.1038/nature10856.
25. Boterman, W.R., and Musterd, S. (2016). Cocooning urban life: Exposure to diversity in neighbourhoods, workplaces and transport. Cities *59*, 139–147. https://doi.org/10.1016/j.cities.2015.10.018.
26. Hilman, R.M., Iñiguez, G., and Karsai, M. (2022). Socioeconomic biases in urban mixing patterns of US metropolitan areas. EPJ Data Sci. *11*, 32. https://doi.org/10.1140/epjds/s13688-022-00341-x.
27. Bassolas, A., Barbosa-Filho, H., Dickinson, B., Dotiwalla, X., Eastham, P., Gallotti, R., Ghoshal, G., Gipson, B., Hazarie, S.A., Kautz, H., et al. (2019). Hierarchical organization of urban mobility and its connection with city livability. Nat. Commun. *10*, 4817. https://doi.org/10.1038/s41467-019-12809-y.
28. Xu, Y., Belyi, A., Santi, P., and Ratti, C. (2019). Quantifying segregation in an integrated urban physical-social space. J. R. Soc. Interface *16*, 20190536. https://doi.org/10.1098/rsif.2019.0536.
29. Xu, F., Wang, Q., Moro, E., Chen, L., Salazar Miranda, A., González, M.C., Tizzoni, M., Song, C., Ratti, C., Bettencourt, L., et al. (2025). Using human mobility data to quantify experienced urban inequalities. Nat. Hum. Behav. *9*, 654–664. https://doi.org/10.1038/s41562-024-02079-0.
30. McNeill, G., Bright, J., and Hale, S.A. (2017). Estimating local commuting patterns from geolocated Twitter data. EPJ Data Sci. *6*, 24. https://doi.org/10.1140/epjds/s13688-017-0120-x.
31. Louail, T., Lenormand, M., Cantu Ros, O.G., Picornell, M., Herranz, R., Frias-Martinez, E., Ramasco, J.J., and Barthelemy, M. (2014). From mobile phone data to the spatial structure of cities. Sci. Rep. *4*, 5276. https://doi.org/10.1038/srep05276.
32. Leo, Y., Fleury, E., Alvarez-Hamelin, J.I., Sarraute, C., and Karsai, M. (2016). Socioeconomic correlations and stratification in social-communication networks. J. R. Soc. Interface *13*, 20160598. https://doi.org/10.1098/rsif.2016.0598.
33. Chen, T., and Guestrin, C. (2016). Xgboost: A scalable tree boosting system. In Proceedings of the 22nd ACM SIGKDD International Conference on Knowledge Discovery and Data Mining, San Francisco (New York: Association for Computing Machinery), pp. 785–794.
34. Lundberg, S.M., and Lee, S.I. (2017). A unified approach to interpreting model predictions. In Proc. 31st Conf. on Neural Inf. Process. Syst., Ulrike von Luxburg and Isabelle Guyon, eds. (New York: Curran Associates Inc.), pp. 4768–4777.
35. Martilli, A. (2014). An idealized study of city structure, urban climate, energy consumption, and air quality. Urban Clim. *10*, 430–446. https://doi.org/10.1016/j.uclim.2014.03.003.
36. Stone, B., Jr. (2008). Urban sprawl and air quality in large US cities. J. Environ. Manage. *86*, 688–698. https://doi.org/10.1016/j.jenvman.2006.12.034.
37. Barbosa, H., de Lima-Neto, F.B., Evsukoff, A., and Menezes, R. (2015). The effect of recency to human mobility. EPJ Data Sci. *4*, 21. https://doi.org/10.1140/epjds/s13688-015-0059-8.
38. Alessandretti, L., Sapiezynski, P., Sekara, V., Lehmann, S., and Baronchelli, A. (2018). Evidence for a conserved quantity in human mobility. Nat. Hum. Behav. *2*, 485–491. https://doi.org/10.1038/s41562-018-0364-x.
39. Hayes, R.B., Lim, C., Zhang, Y., Cromar, K., Shao, Y., Reynolds, H.R., Silverman, D.T., Jones, R.R., Park, Y., Jerrett, M., et al. (2020). PM2.5 air pollution and cause-specific cardiovascular disease mortality. Int. J. Epidemiol. *49*, 25–35. https://doi.org/10.1093/ije/dyz114.
40. Liu, Y., Wei, W., Sun, A., and Miao, C. (2014). Exploiting geographical neighborhood characteristics for location recommendation. In Proc. 23rd ACM Int. Conf. on Inf. Knowl. Manage., pp. 739–748. https://doi.org/10.1145/2661829.2662002.
41. Abbiasov, T., Heine, C., Sabouri, S., Salazar-Miranda, A., Santi, P., Glaeser, E., and Ratti, C. (2024). The 15-minute city quantified using human mobility data. Nat. Hum. Behav. *8*, 445–455.
42. U.S. Census Bureau. American community survey. https://www.census.gov/data.html.
43. OpenStreetMap contributors. OpenStreetMap. https://www.openstreetmap.org.
44. Crippa, M., Guizzardi, D., Pagani, F., Schiavina, M., Melchiorri, M., Pisoni, E., Graziosi, F., Muntean, M., Maes, J., Dijkstra, L., et al. (2024). Insights into the spatial distribution of global, national, and subnational greenhouse gas emissions in the Emissions Database for Global Atmospheric Research (EDGAR v8.0). Earth Syst. Sci. Data *16*, 2811–2830. https://doi.org/10.5194/essd-16-2811-2024.
45. Lei, D., Chen, X., Cheng, L., Zhang, L., Ukkusuri, S.V., and Witlox, F. (2020). Inferring temporal motifs for travel pattern analysis using large scale smart card data. Transp. Res. Pt. C-Emerg. Technol. *120*, 102810. https://doi.org/10.1016/j.trc.2020.102810.
46. Blesch, K., Hauser, O.P., and Jachimowicz, J.M. (2022). Measuring inequality beyond the Gini coefficient may clarify conflicting findings. Nat. Hum. Behav. *6*, 1525–1536. https://doi.org/10.1038/s41562-022-01430-7.
47. Chen, Y. (2025). Data for Promoting sustainable human mobility for income segregation mitigation. Zenodo. https://doi.org/10.5281/zenodo.15314012.
48. Chen, Y. (2024). Segregation-constrained human mobility model. Zenodo. https://doi.org/10.5281/zenodo.13742568.

Patterns, Volume *7*

Supplemental information

Promoting sustainable human mobility for income segregation mitigation

Yong Chen, Chenlei Liao, Zeen Cai, Wanru Wang, Yingji Xia, Xiqun (Michael) Chen, Jianjun Wu, and Ziyou Gao

Supplemental Information

Supplemental figures

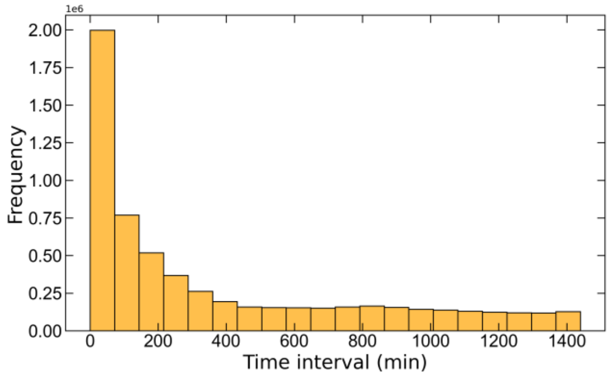

Figure S1. Time interval distribution between two consecutive trips for all individuals, with most individuals exhibiting time intervals within four hours between visits.

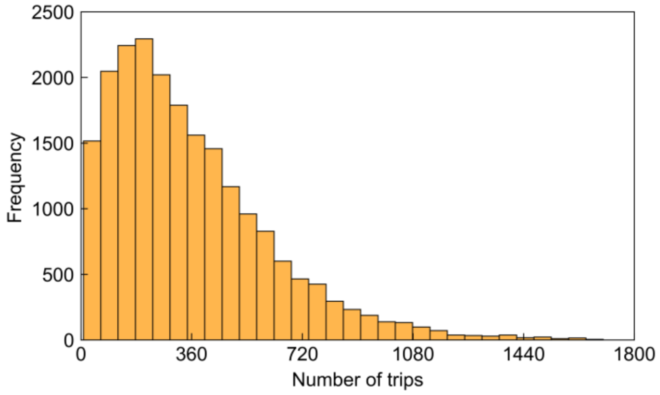

Figure S2. The distribution of trips of all individuals.

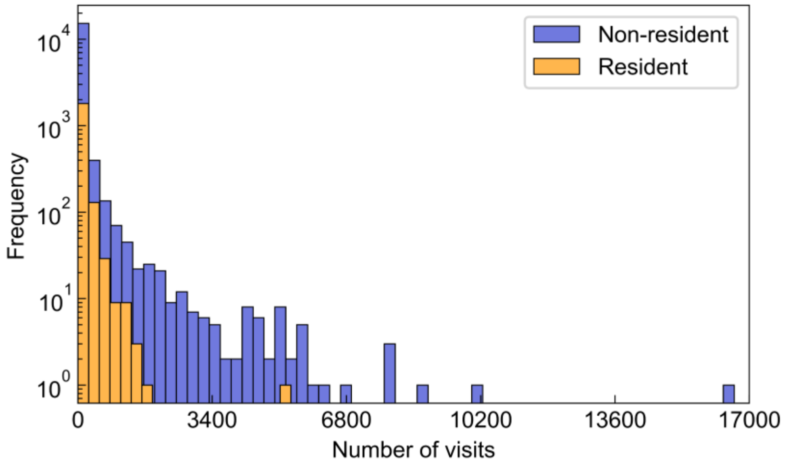

Figure S3. Distribution of visits to each census tract.

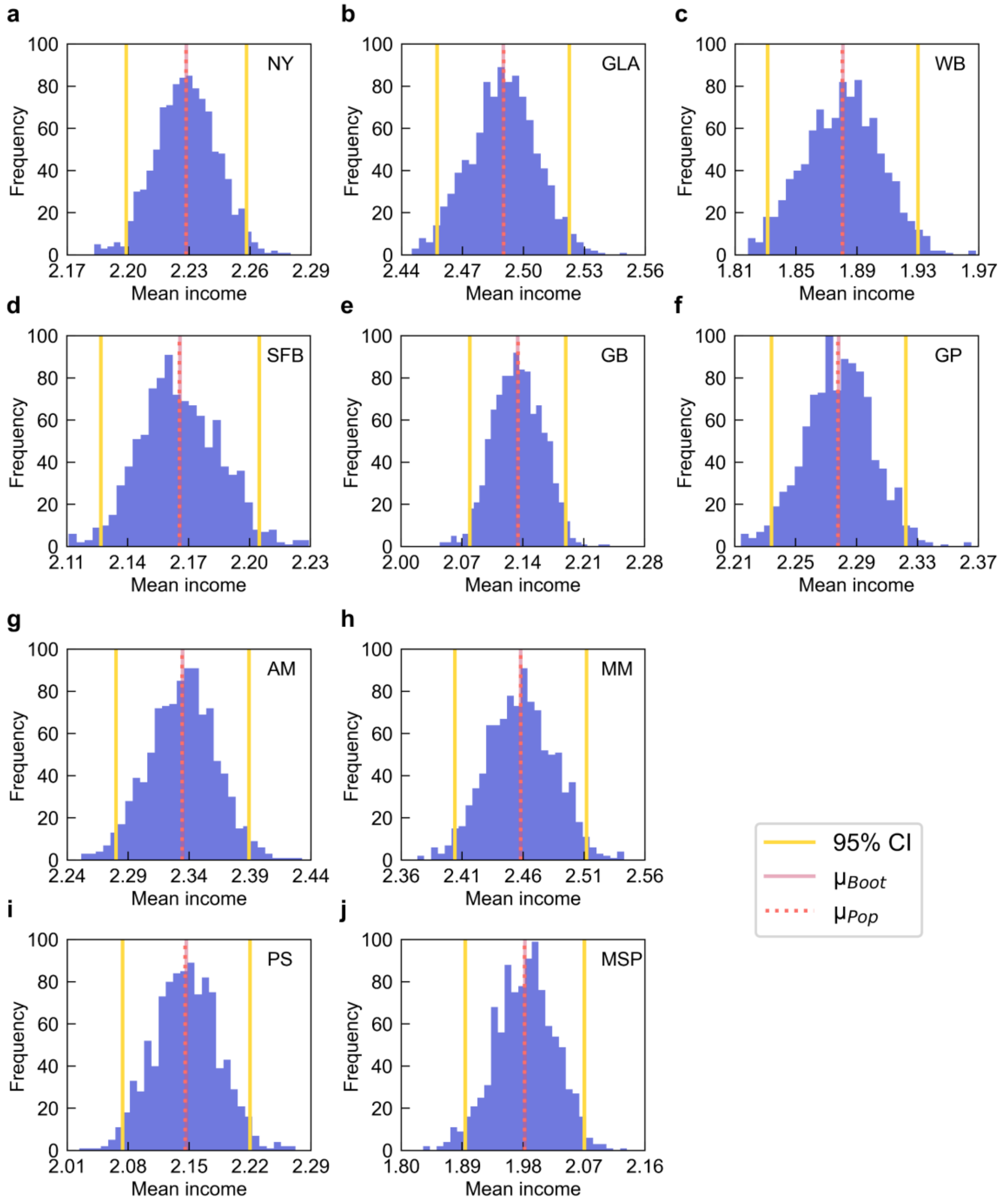

Figure S4. Bootstrapping results. The histogram represents the distribution of mean user income across the 1,000 resampled datasets obtained via bootstrapping. The yellow line denotes the 95% confidence interval for the mean income distribution. The pink line indicates the mean income across all sample sets. The red dashed line represents the mean income value of the actual user population.

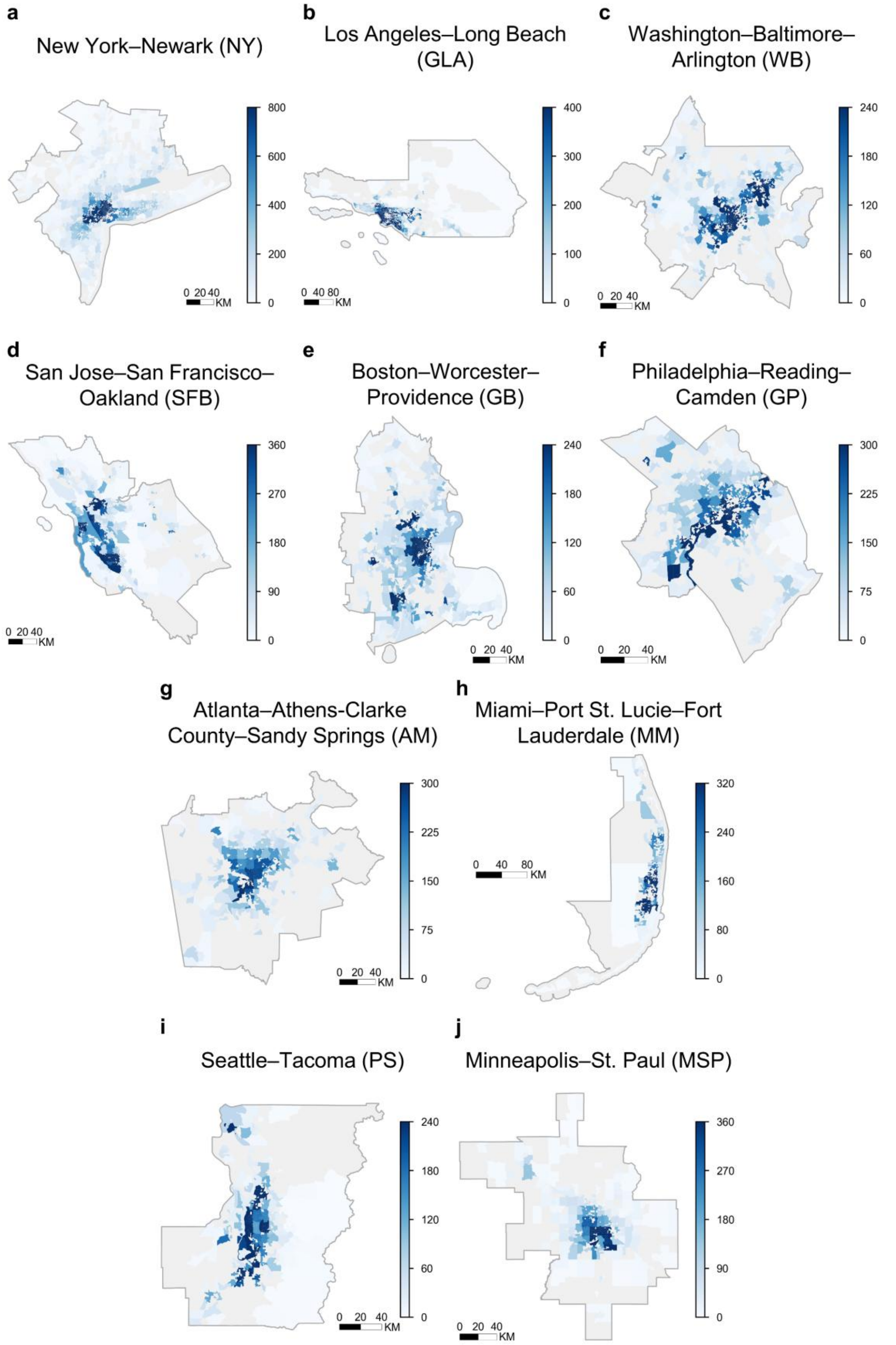

Figure S5. Distribution of total annual PM2.5 emissions for all locations across all large cities. The color of each census tract indicates its corresponding PM2.5 emissions in tonnes.

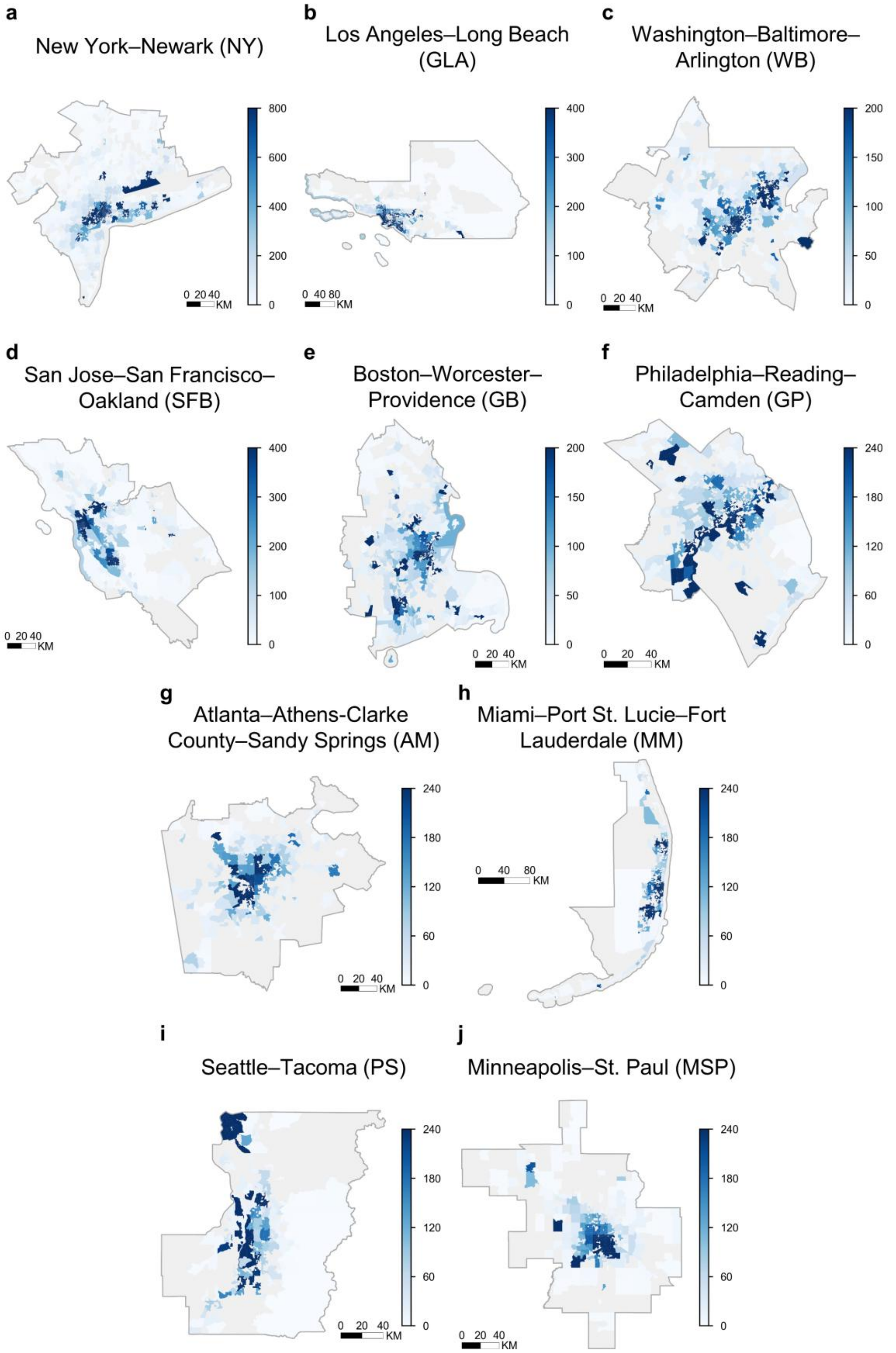

Figure S6. Distribution of total annual SO_2 emissions for all locations across all large cities. The color of each census tract indicates its corresponding SO_2 emissions in tonnes.

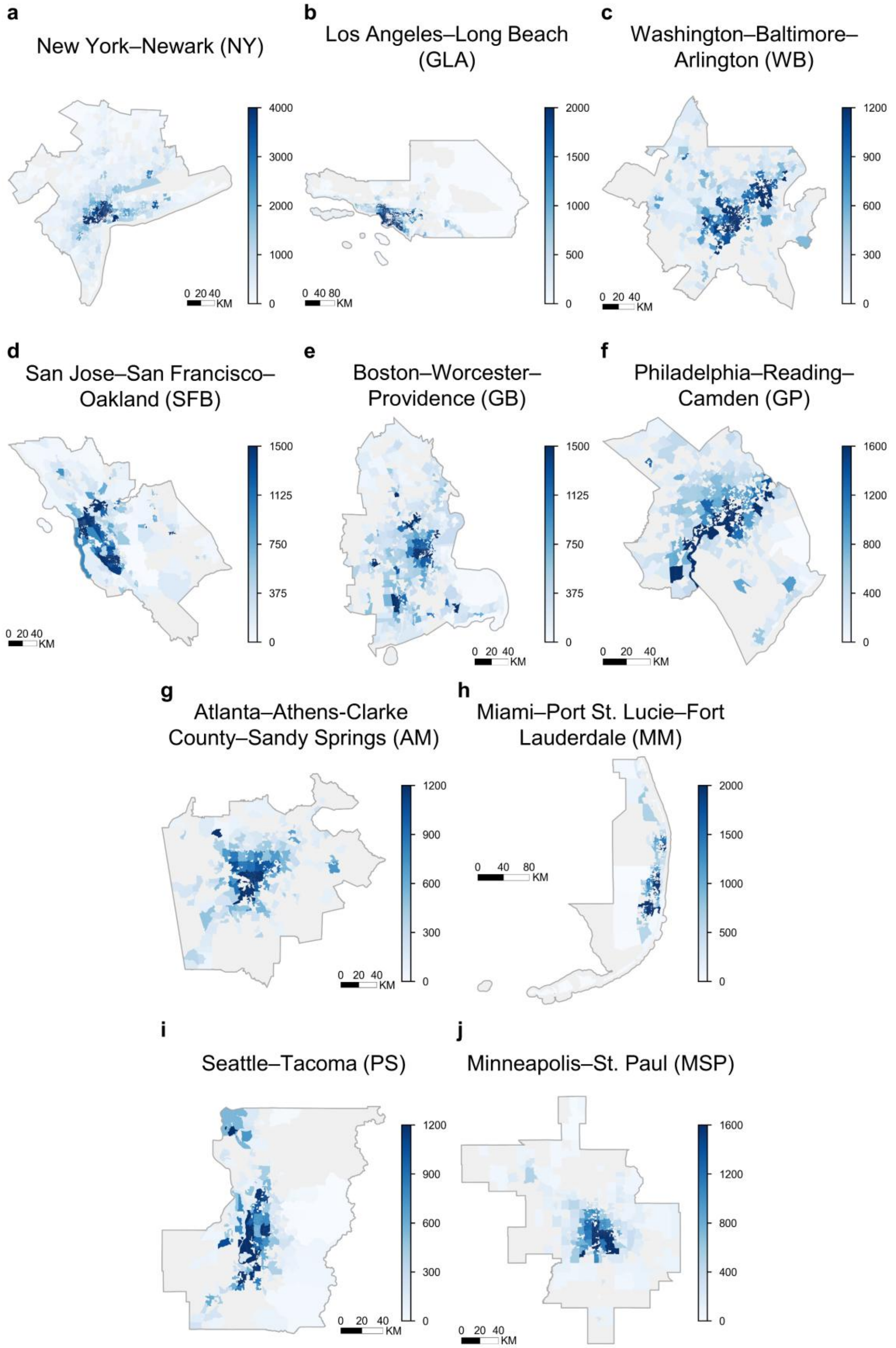

Figure S7. Distribution of total annual NO_x emissions for all locations across all large cities. The color of each census tract indicates its corresponding NO_x emissions in tonnes.

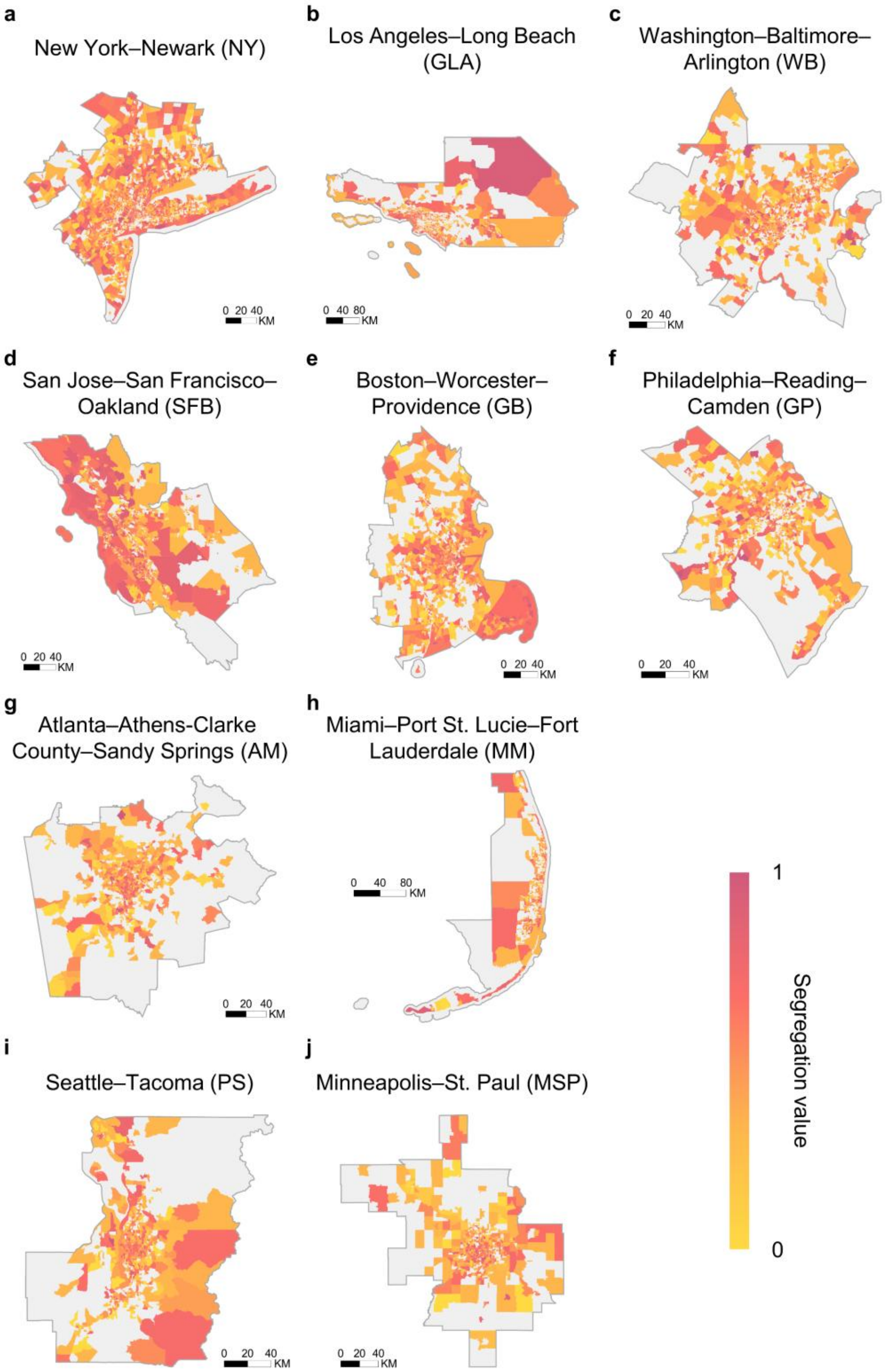

Figure S8. Distribution of income segregation values experienced in all locations across all large cities. The color of each census tract indicates its corresponding segregation value.

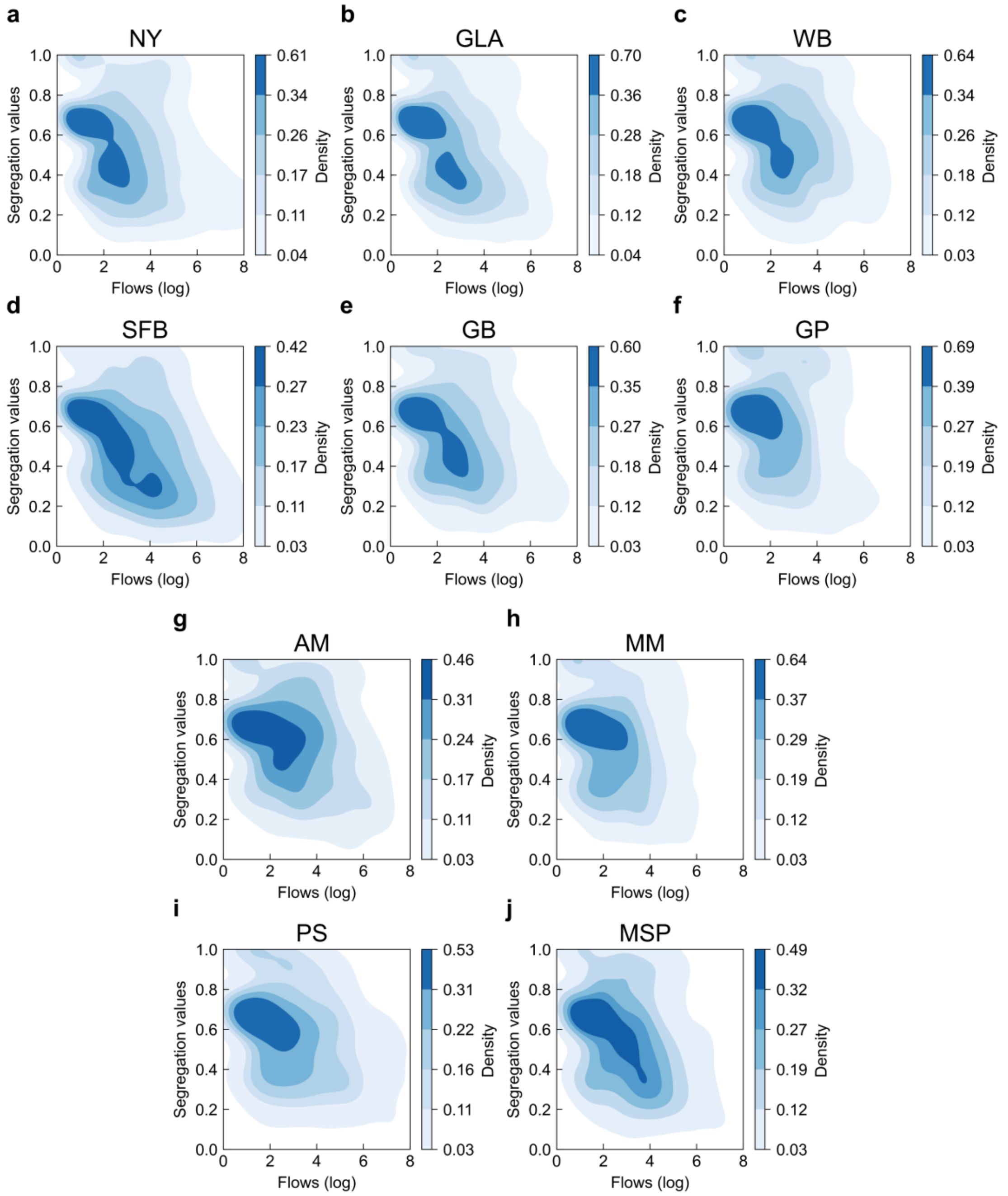

Figure S9. Correlation of mobility flows and segregation values for all census tracts within each city. The colors represent the density of the scattered data points.

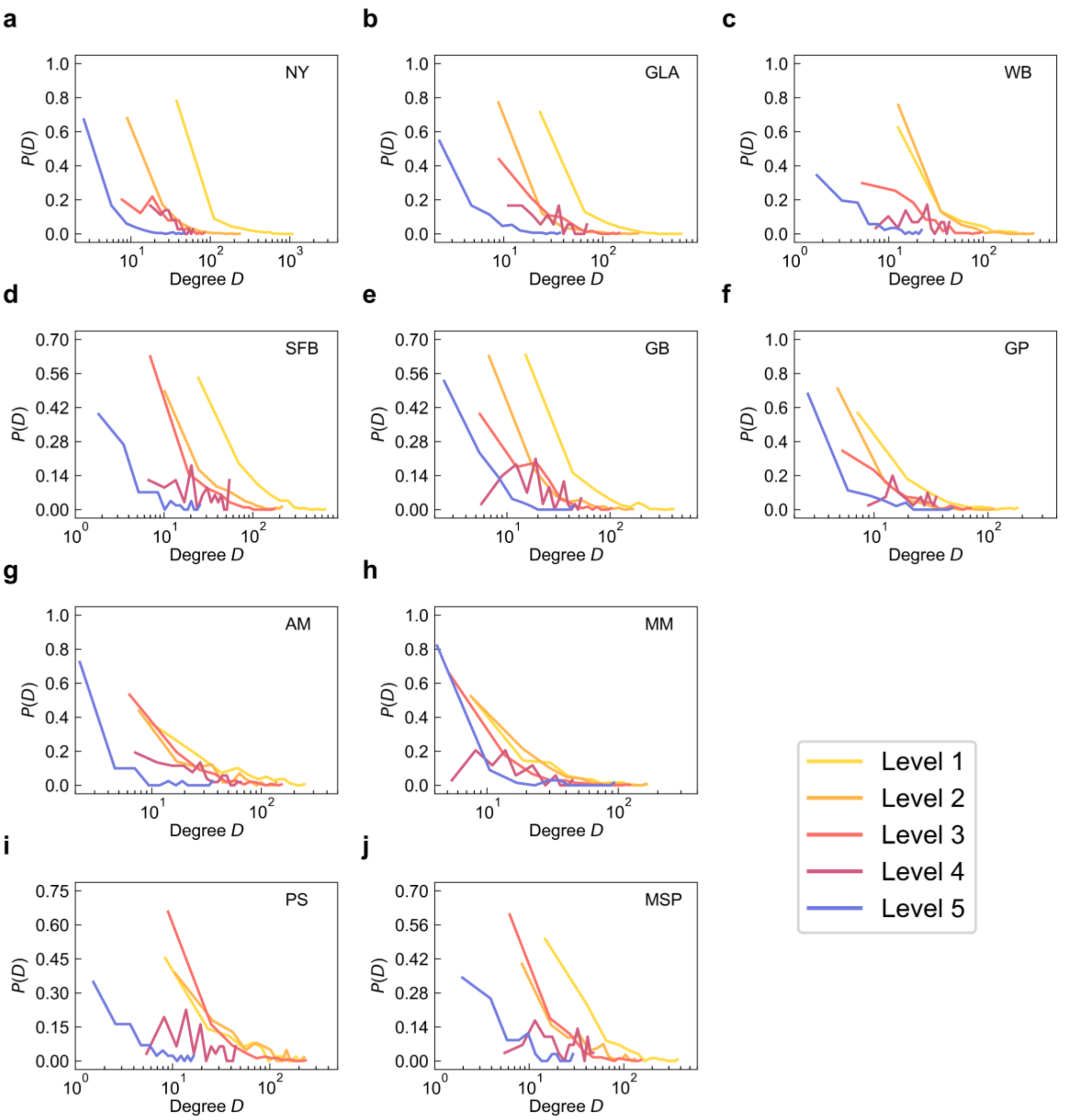

Figure S10. Travel degree distribution of locations across different segregation levels for all large cities.

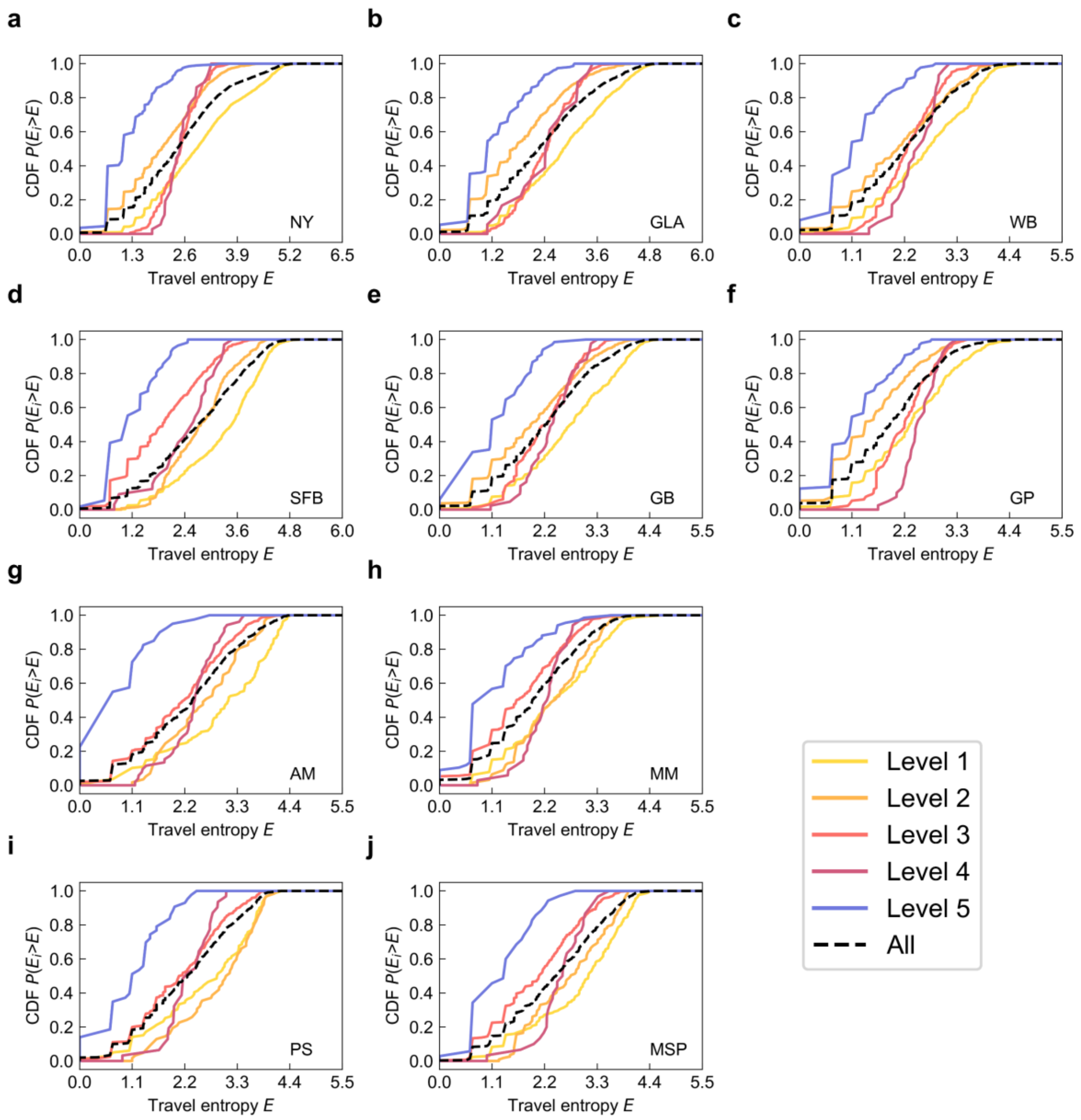

Figure S11. Travel entropy distribution of locations across different segregation levels for all large cities. The black dashed line represents the travel entropy distribution for all locations.

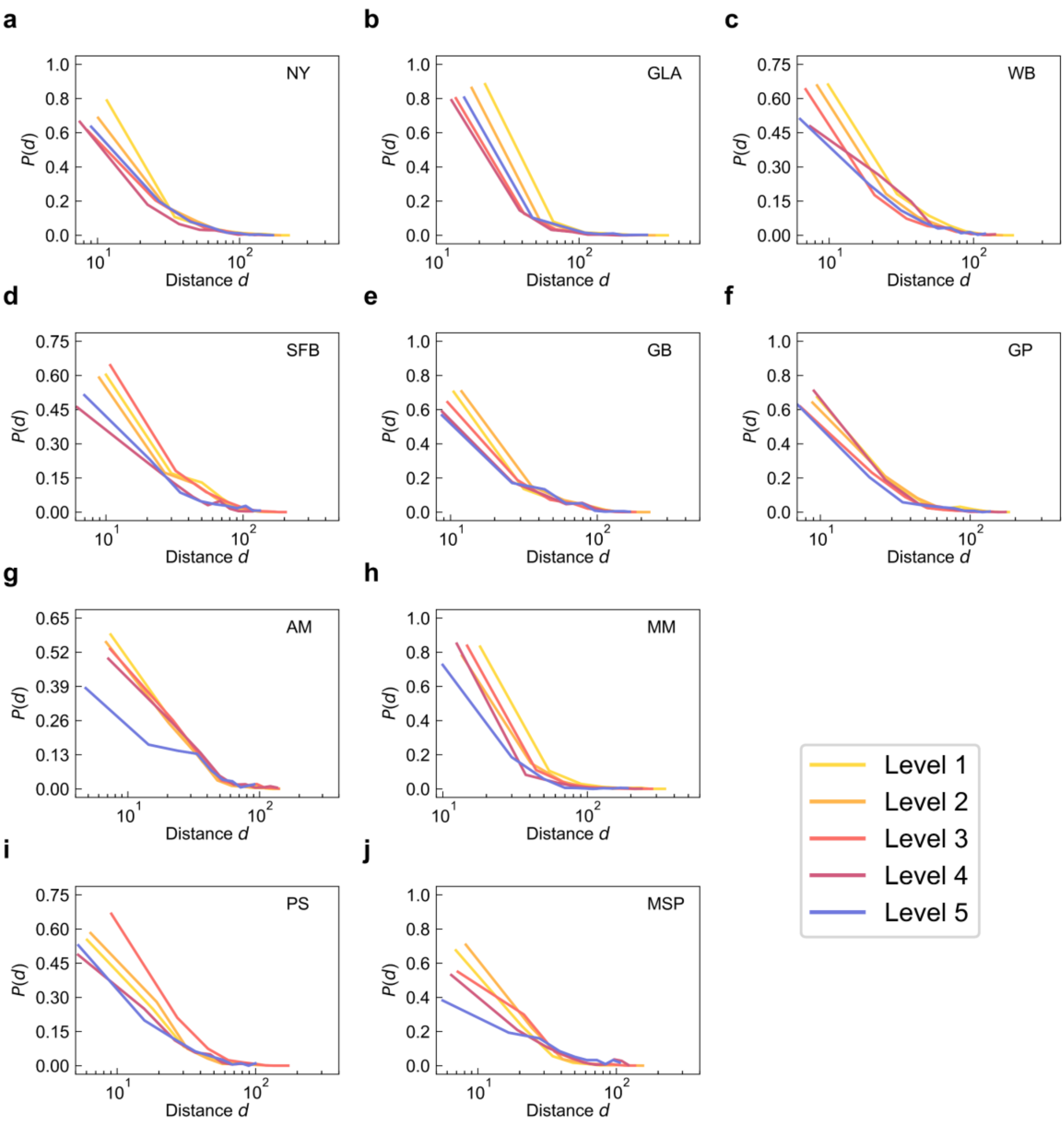

Figure S12. Travel distance distribution of locations across different segregation levels for all large cities.

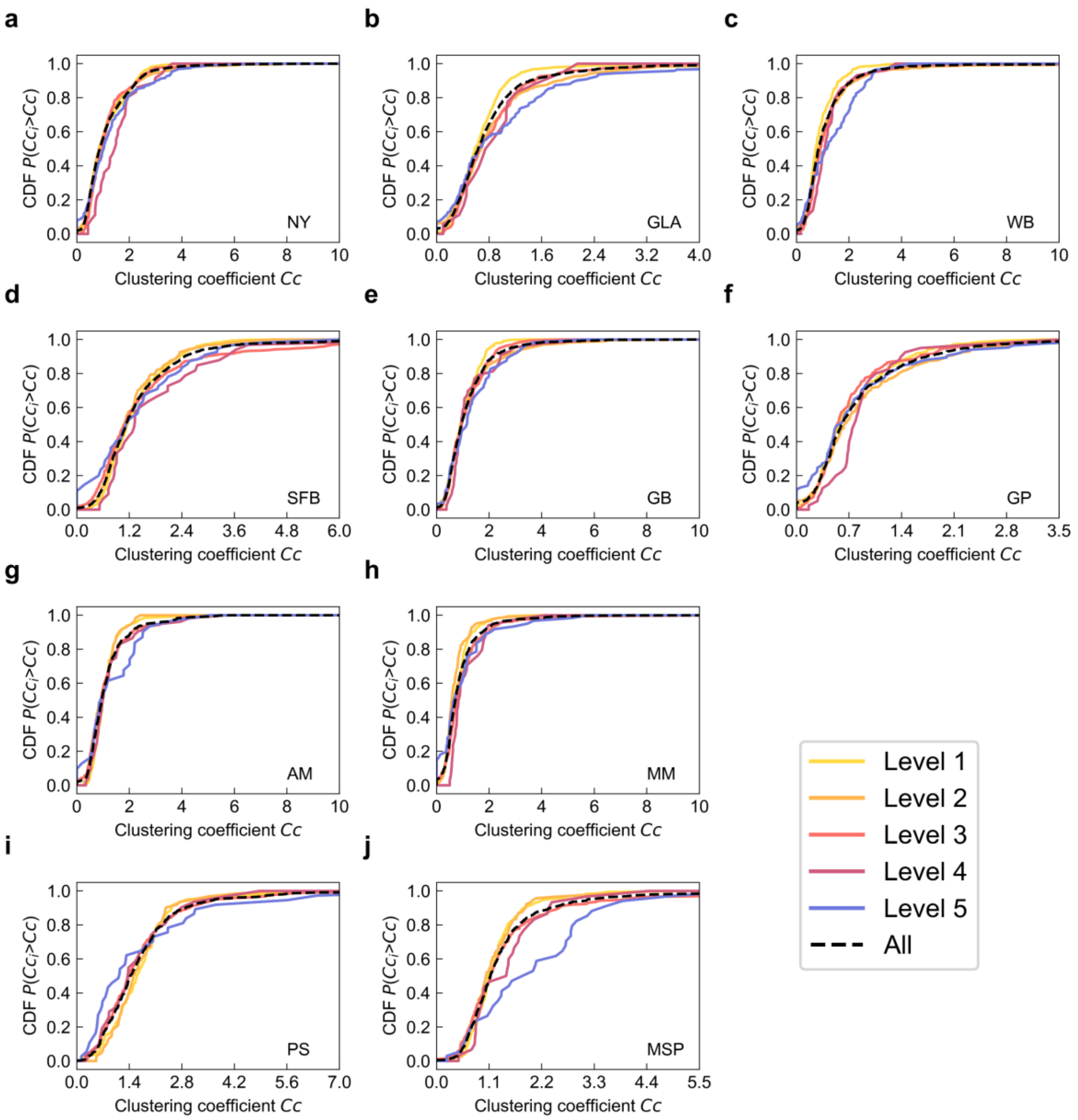

Figure S13. Clustering coefficient distribution of locations across different segregation levels for all large cities. The black dashed line represents the clustering coefficient distribution for all locations.

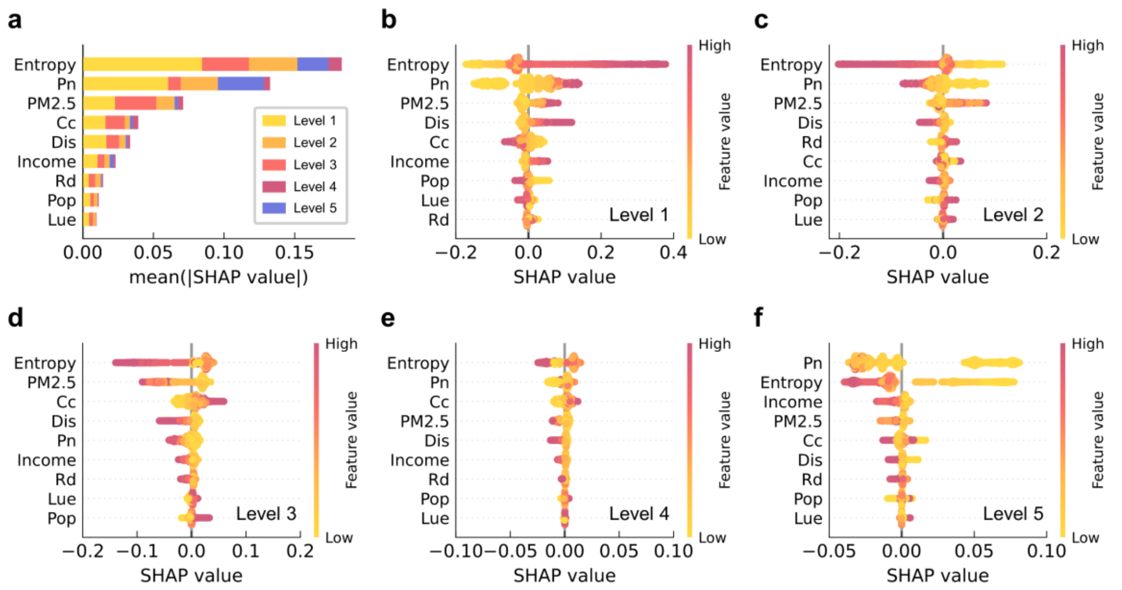

Figure S14. Impact of variables on segregation level classification. **a** Comparison assessment of the relative importance of features across segregation levels, with longer bars indicating greater significance. 'Pn', 'Pop', 'Rd', 'Dis', 'Cc', 'Lue', and 'PM2.5' denote the number of points of interest categories, population size, road density, travel distance, clustering coefficient, land use entropy, and local annual PM2.5 emissions, respectively. **b-f** Distribution of SHAP values of all features within each segregation level. Each scatter point represents a census tract, with color indicating feature value. Points positioned to the right of the x-axis signify a positive impact on model output, and vice versa.

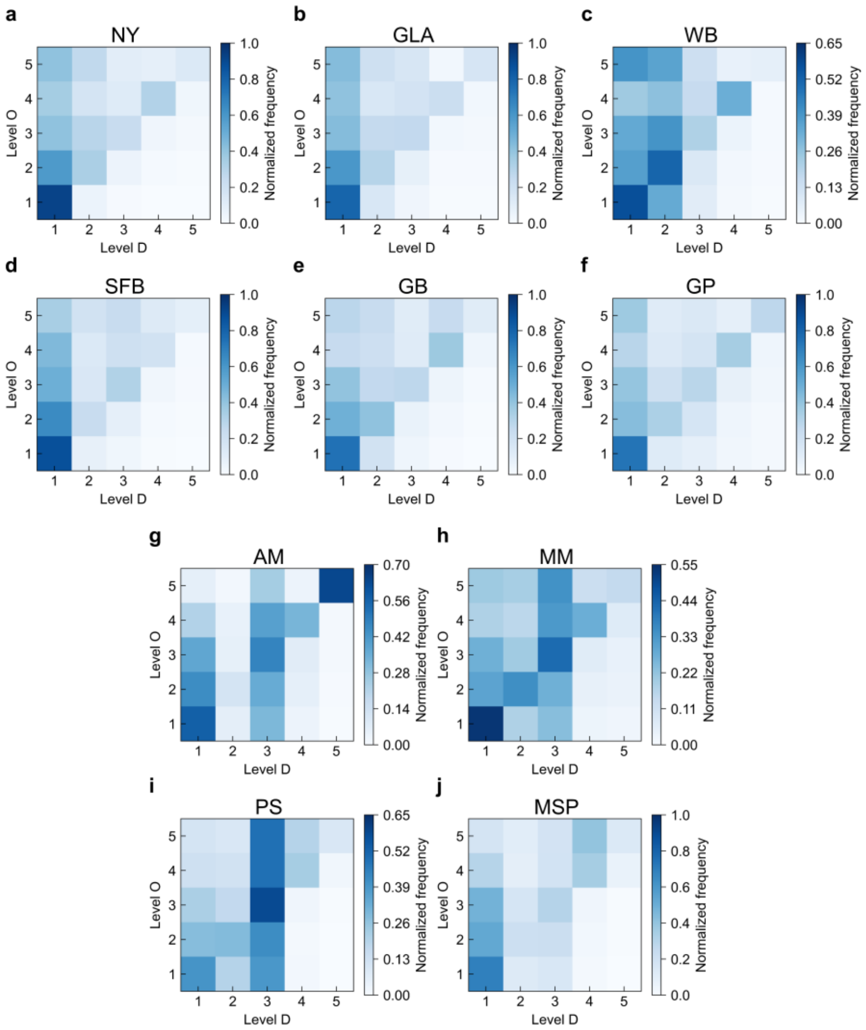

Figure S15. Segregation-constrained visitation matrix for 10 large cities. Color represents the visitation frequency between each segregation level, which is normalized based on the total number of trips from the origin level.

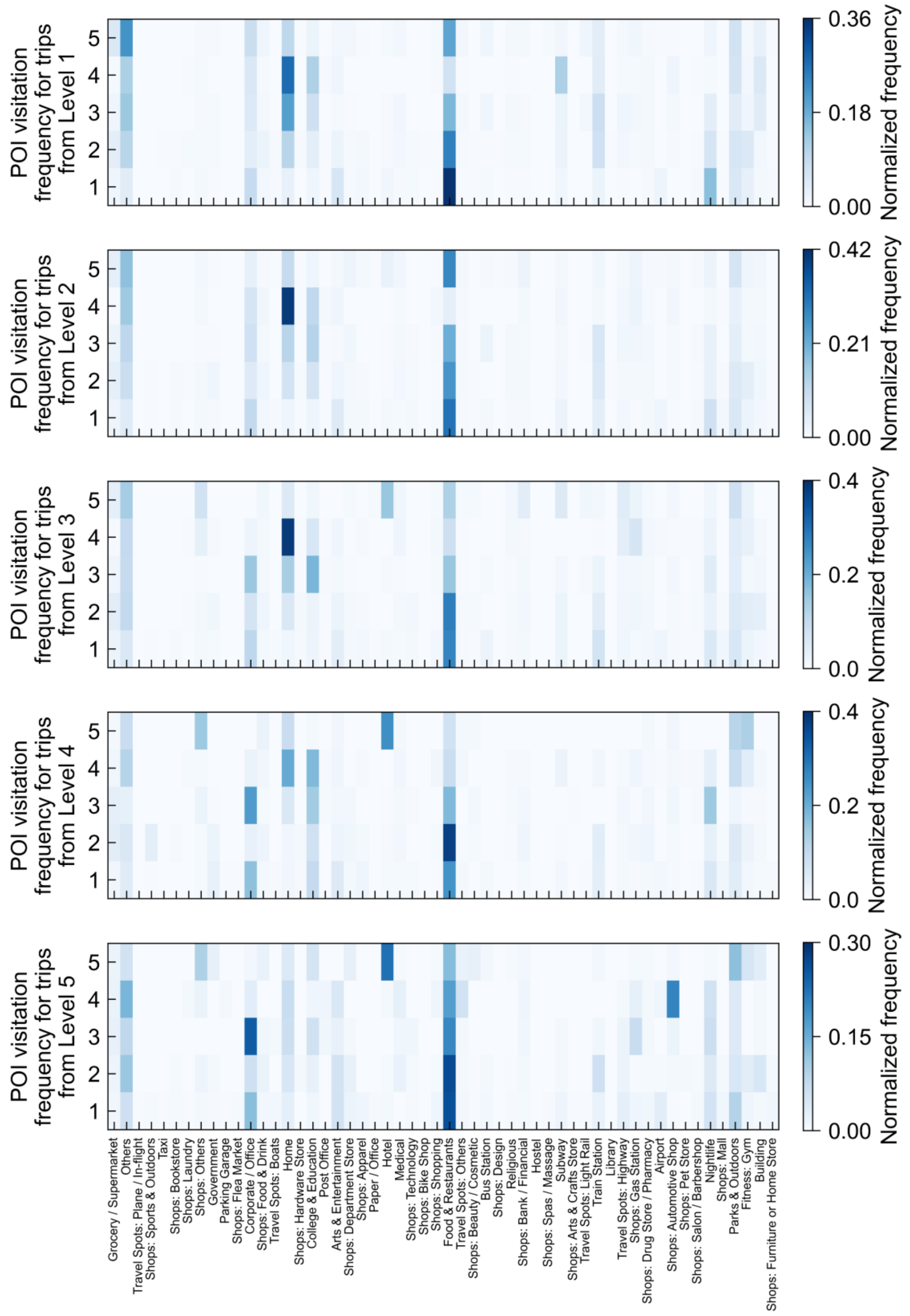

Figure S16. Distribution of POI visitation frequencies for trips from locations at a given segregation level to other locations with different segregation levels in the New York–Newark CSA. Color indicates the visitation frequencies between each segregation level, normalized based on the total number of trips to the destination level.

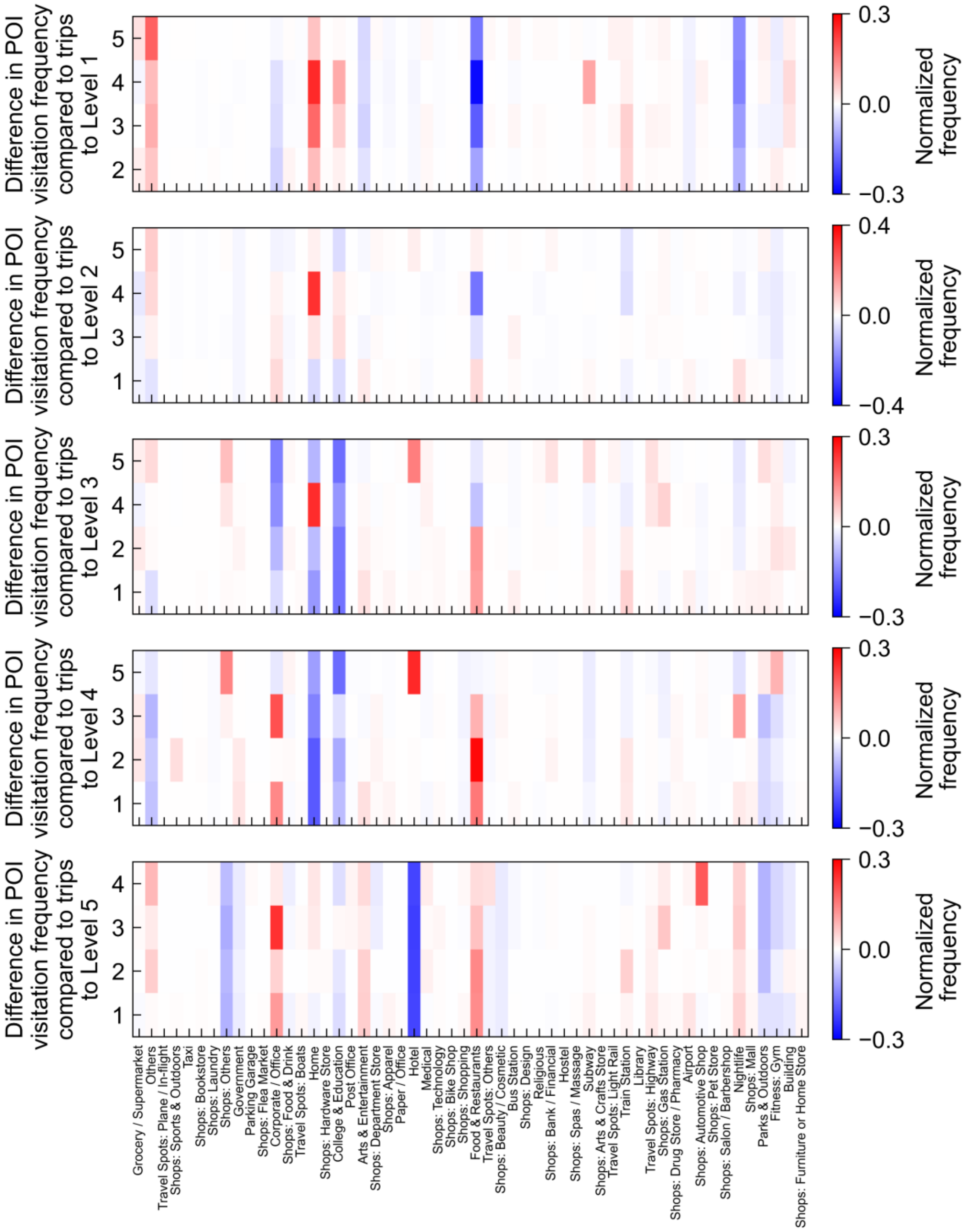

Figure S17. Differences in POI visitation frequencies for trips to locations with other segregation levels and to locations with the same segregation level as the departure location in the New York–Newark CSA.

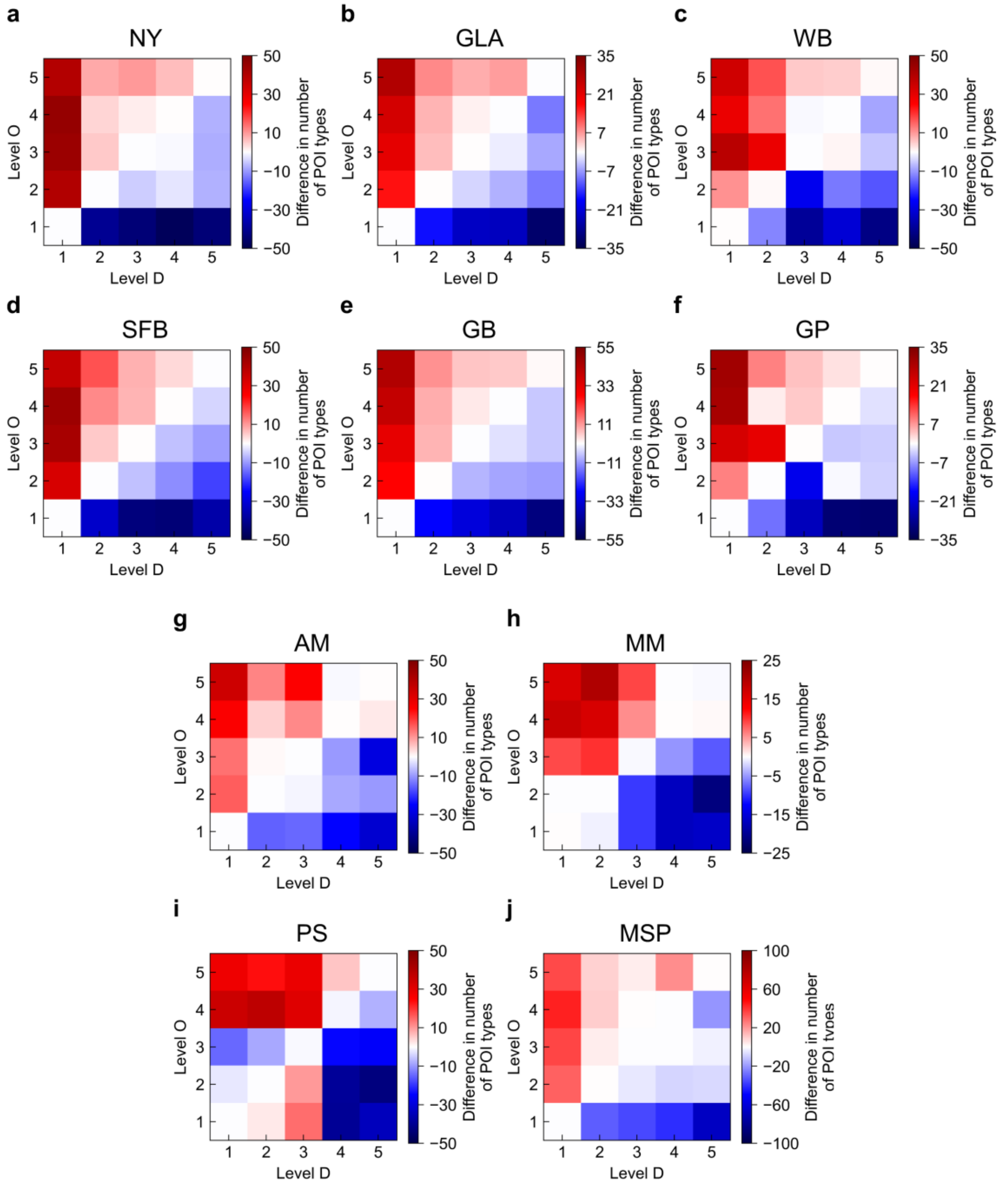

Figure S18. Differences in the number of POI types when traveling from origins with different segregation levels to destinations with different segregation levels. The color represents the average difference in the number of POI types at the destination compared to the origin.

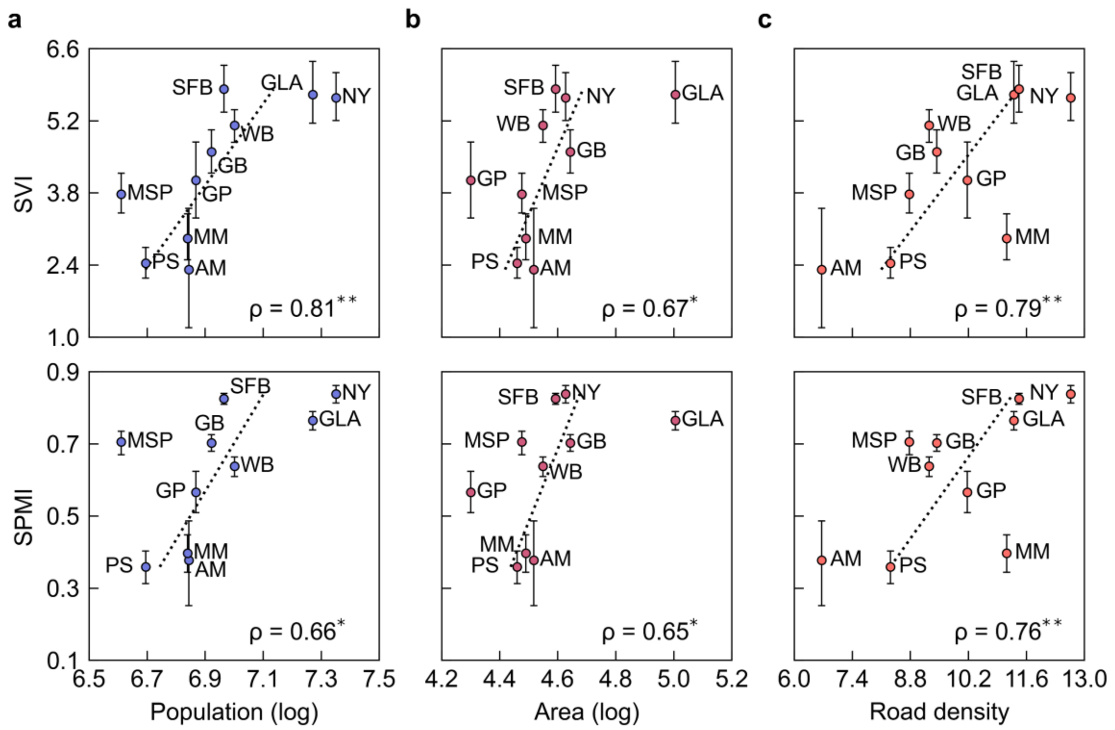

Figure S19. Correlation between two segregation visitation indices and three urban indicators. Panels show the correlation with: **a** Population, **b** Area, **c** Road density. ρ denotes the Spearman correlation coefficient. ** and * indicate that p-value is less than 0.01 and 0.05, respectively.

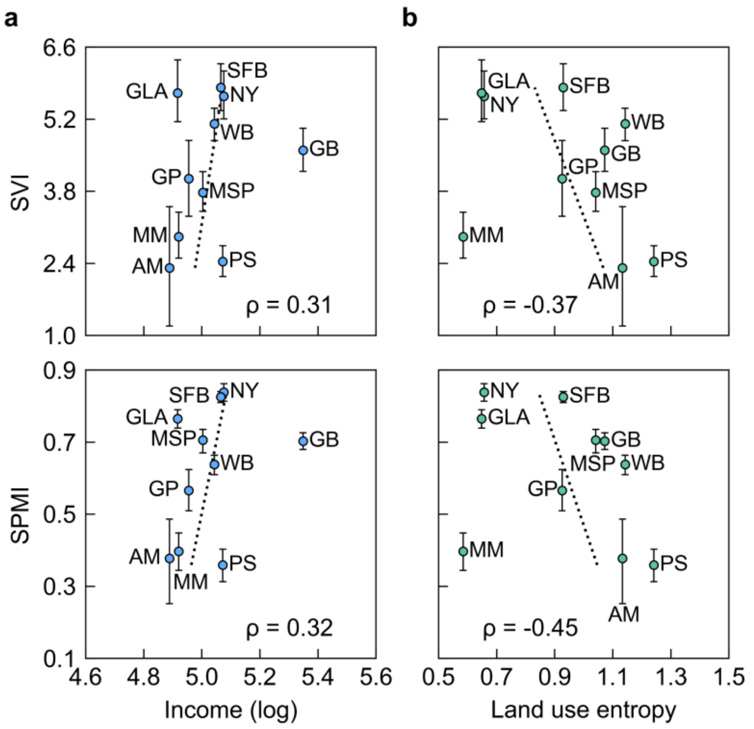

Figure S20. Correlation between two segregation visitation indices and two urban indicators. Panels show the correlation with: **a** Income, **b** Land use entropy. ρ denotes the Spearman correlation coefficient.

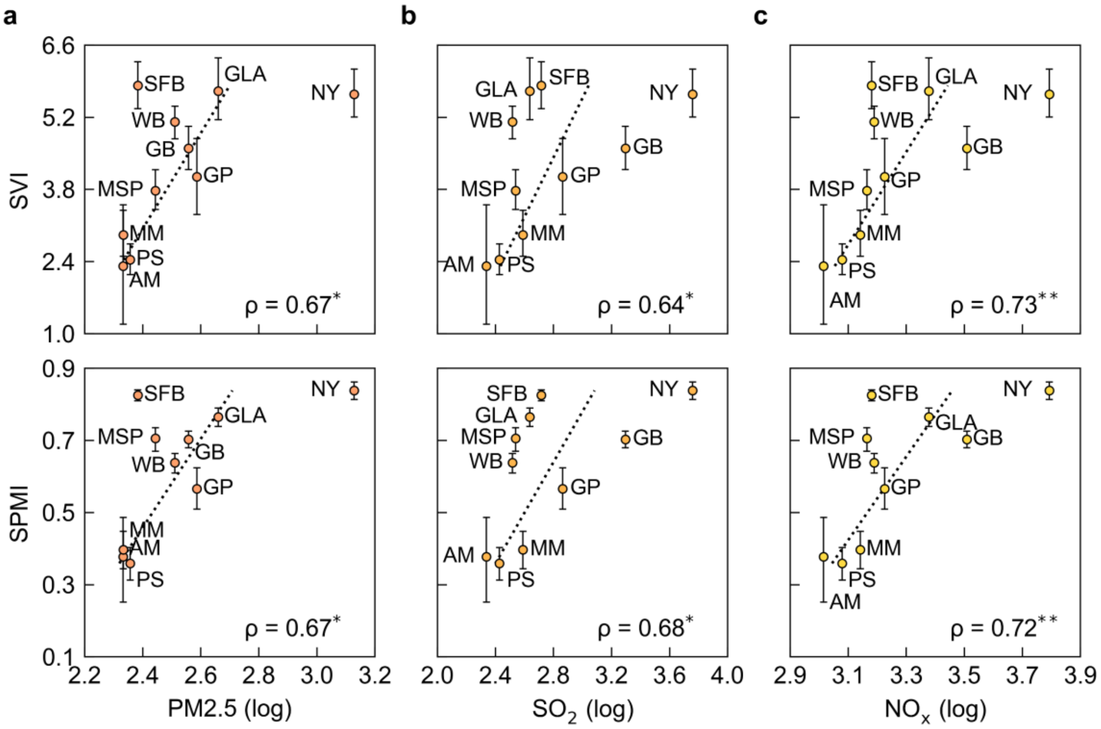

Figure S21. Correlation between two segregation visitation indices and two urban indicators. Panels show the correlation with: **a** PM2.5 emissions, **b** SO_2 emissions, **c** NO_x emissions. Pollutant emissions are the total annual emissions in tons. ρ denotes the Spearman correlation coefficient. ** and * indicate that p-value is less than 0.01 and 0.05, respectively.

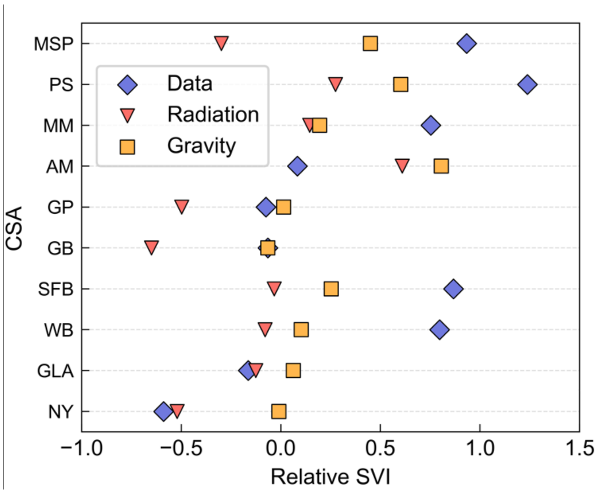

Figure S22. The relative difference between the SVI values of empirically observed (i.e., Data), radiation-based, gravity-based models (see Note S5 for details), and the SVI values of the null-based model.

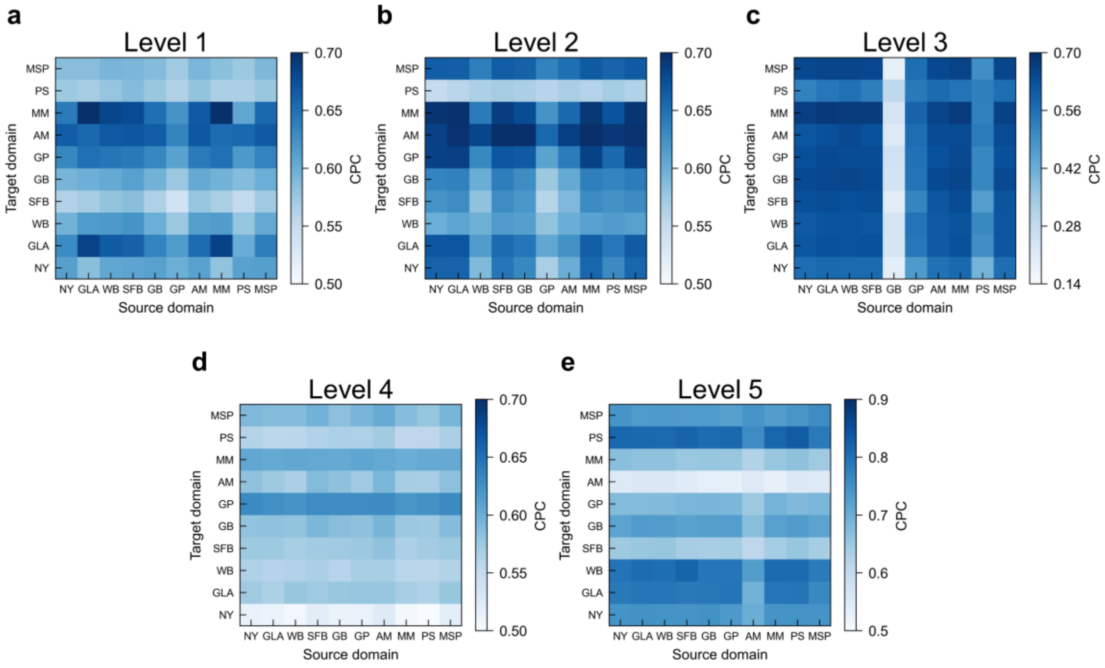

Figure S23. Transfer prediction performance for collective human flows at locations with the same segregation level between different large cities.

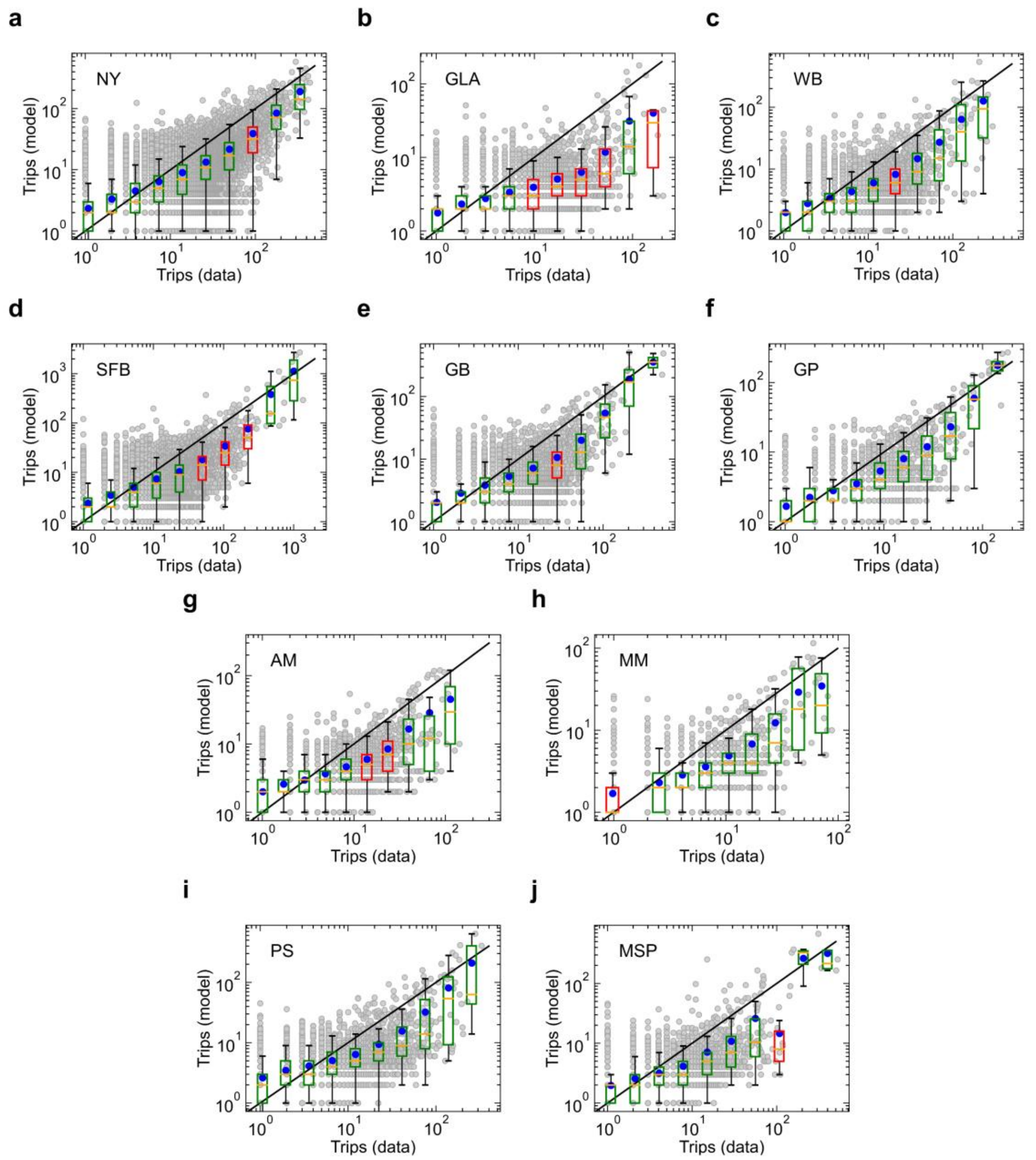

Figure S24. Paired comparisons of predicted and real trips for 10 large cities. Gray points indicate observed and predicted location pairs. The boxplot illustrates how the predicted trips are distributed across various ranges of observed trip counts. A green-shaded box indicates that the diagonal line $y = x$ falls within the 5th and 95th percentiles, and red otherwise. Blue points represent the average predicted trip counts across different bins.

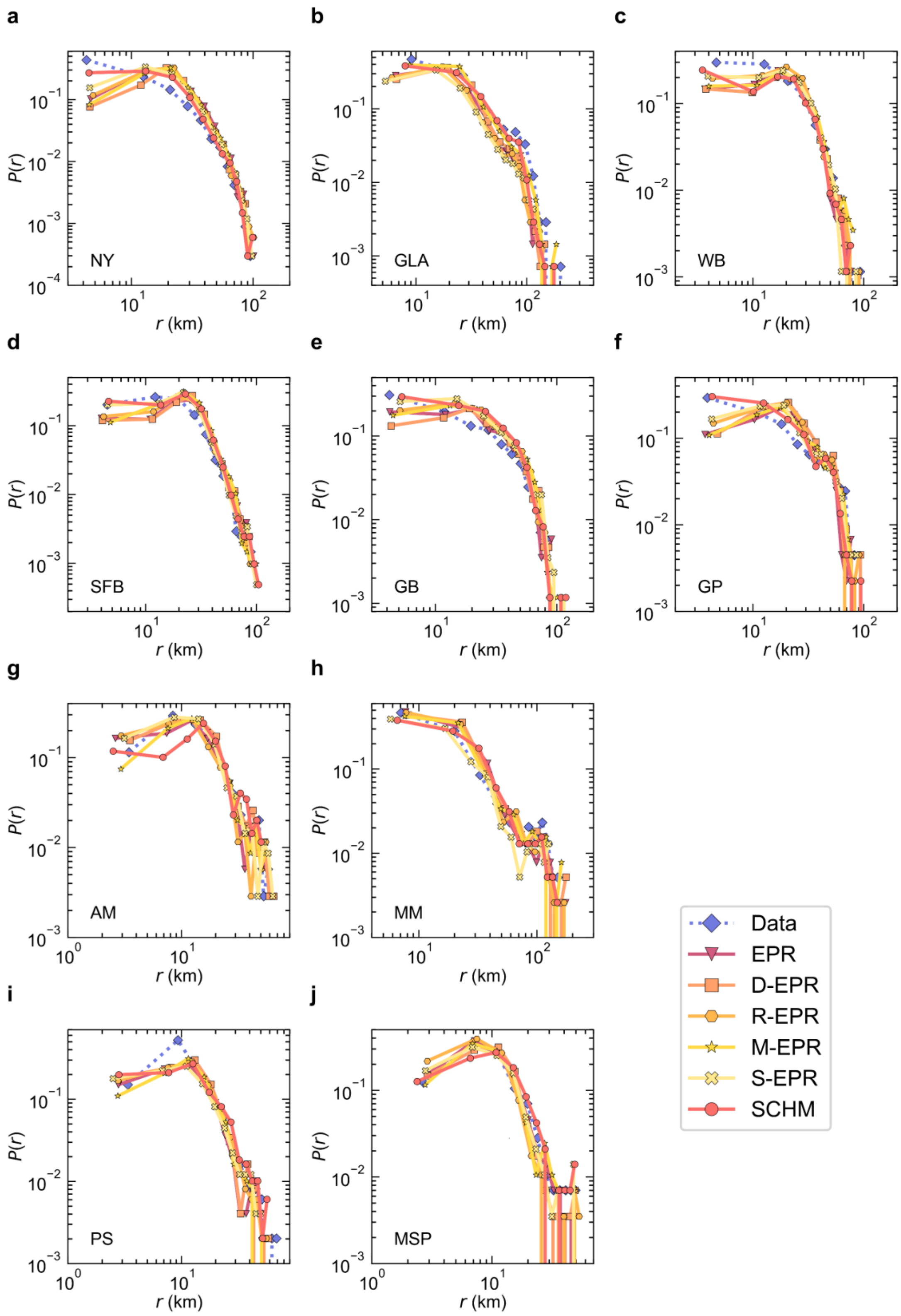

Figure S25. Comparison of radius of gyration distributions for 10 large cities.

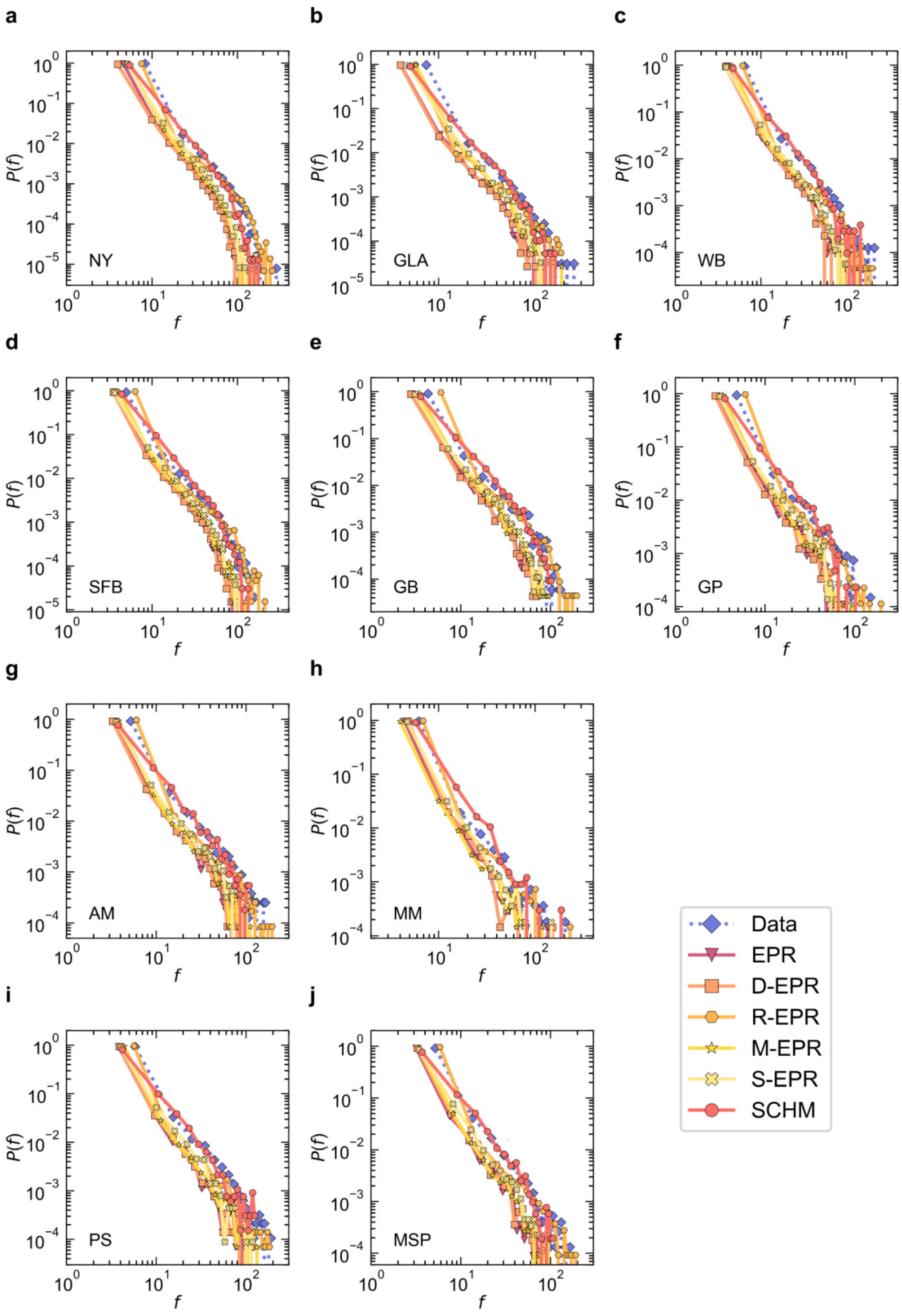

Figure S26. Comparison of distributions of individuals' location visitation frequency for 10 large cities.

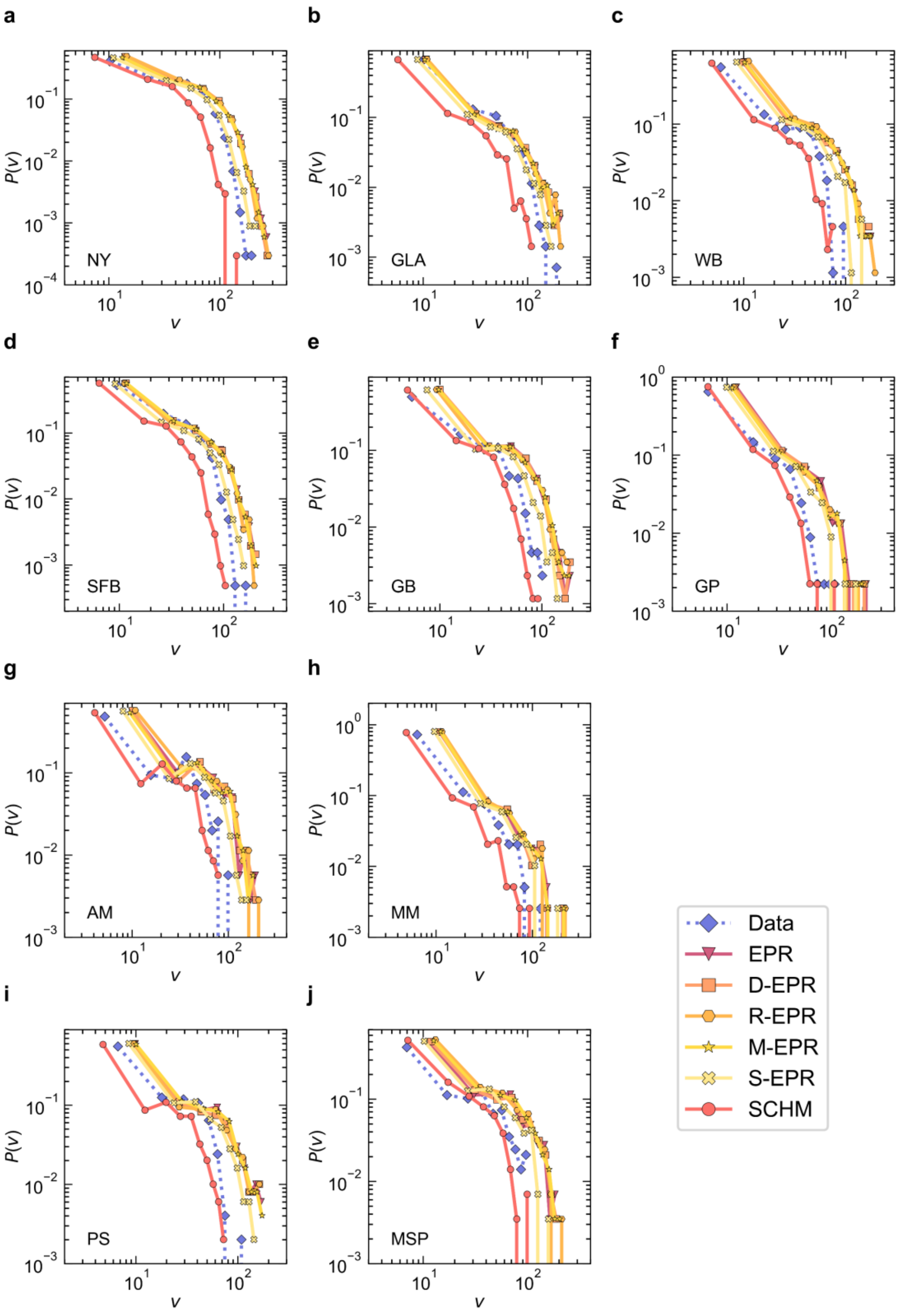

Figure S27. Comparison of distributions of the number of locations visited by individuals for 10 large cities.

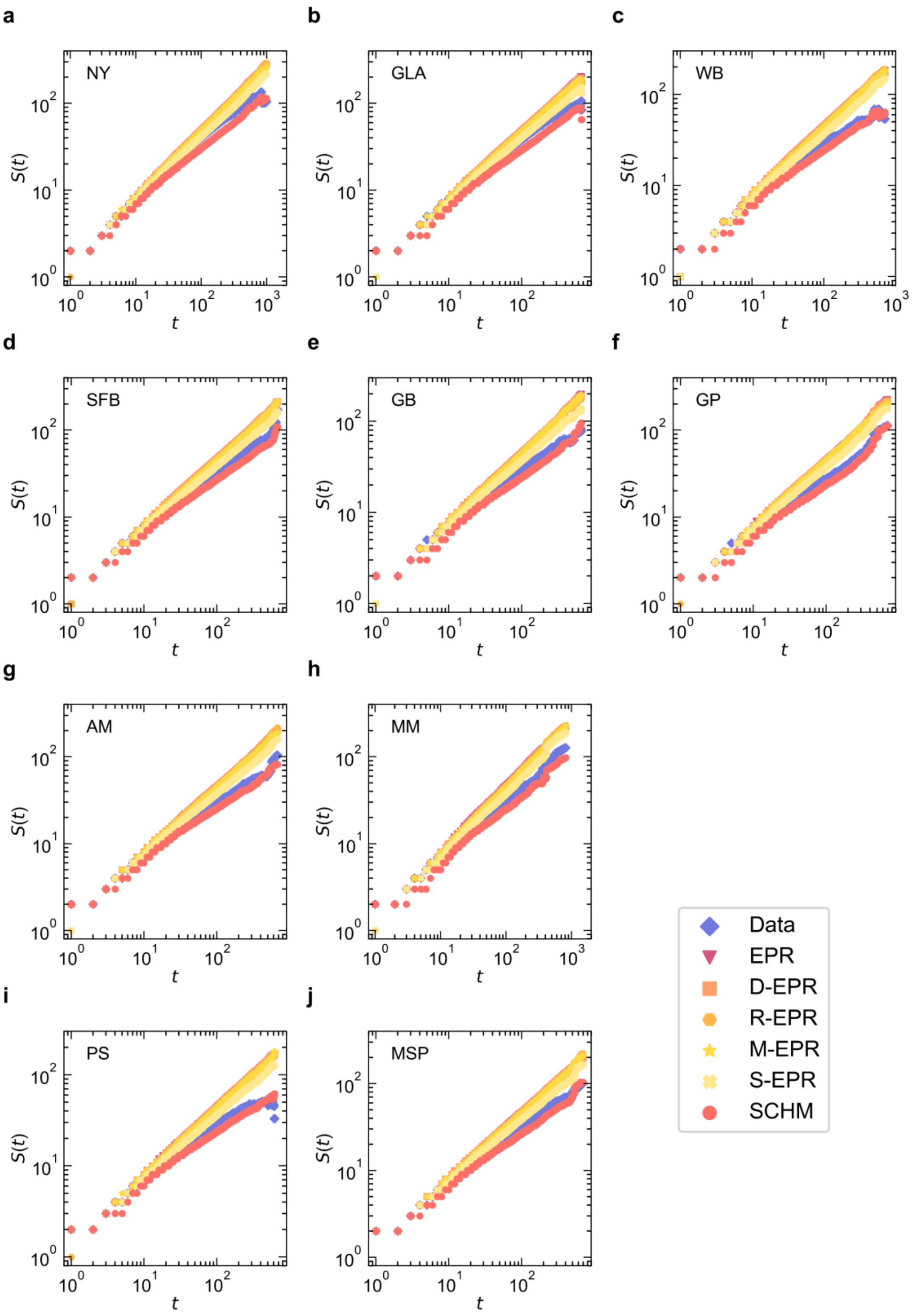

Figure S28. Comparison of distributions of the total number of locations visited within t trips for 10 large city datasets.

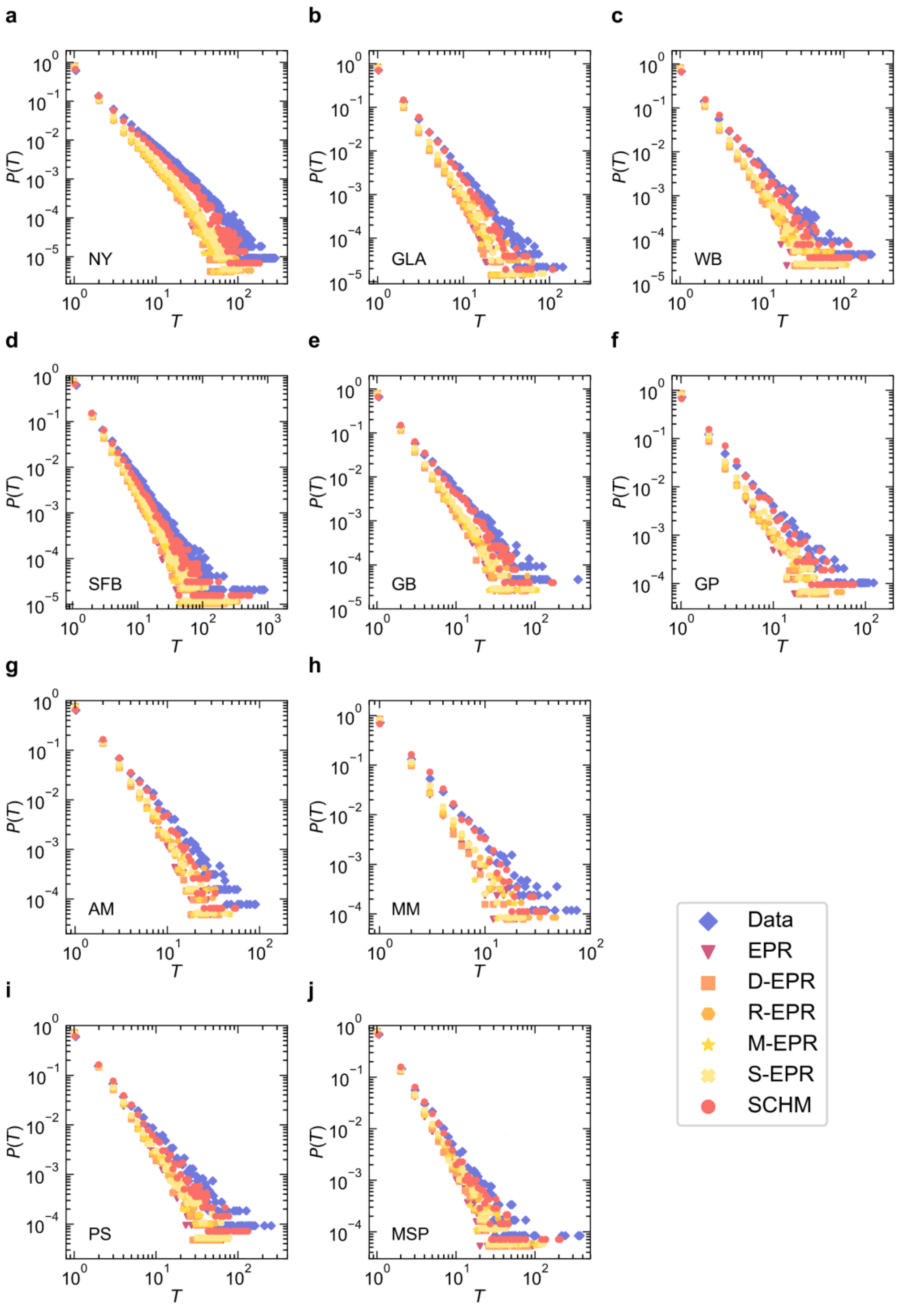

Figure S29. Comparison between predicted and actual distributions of the number of trips between two locations for 10 large cities.

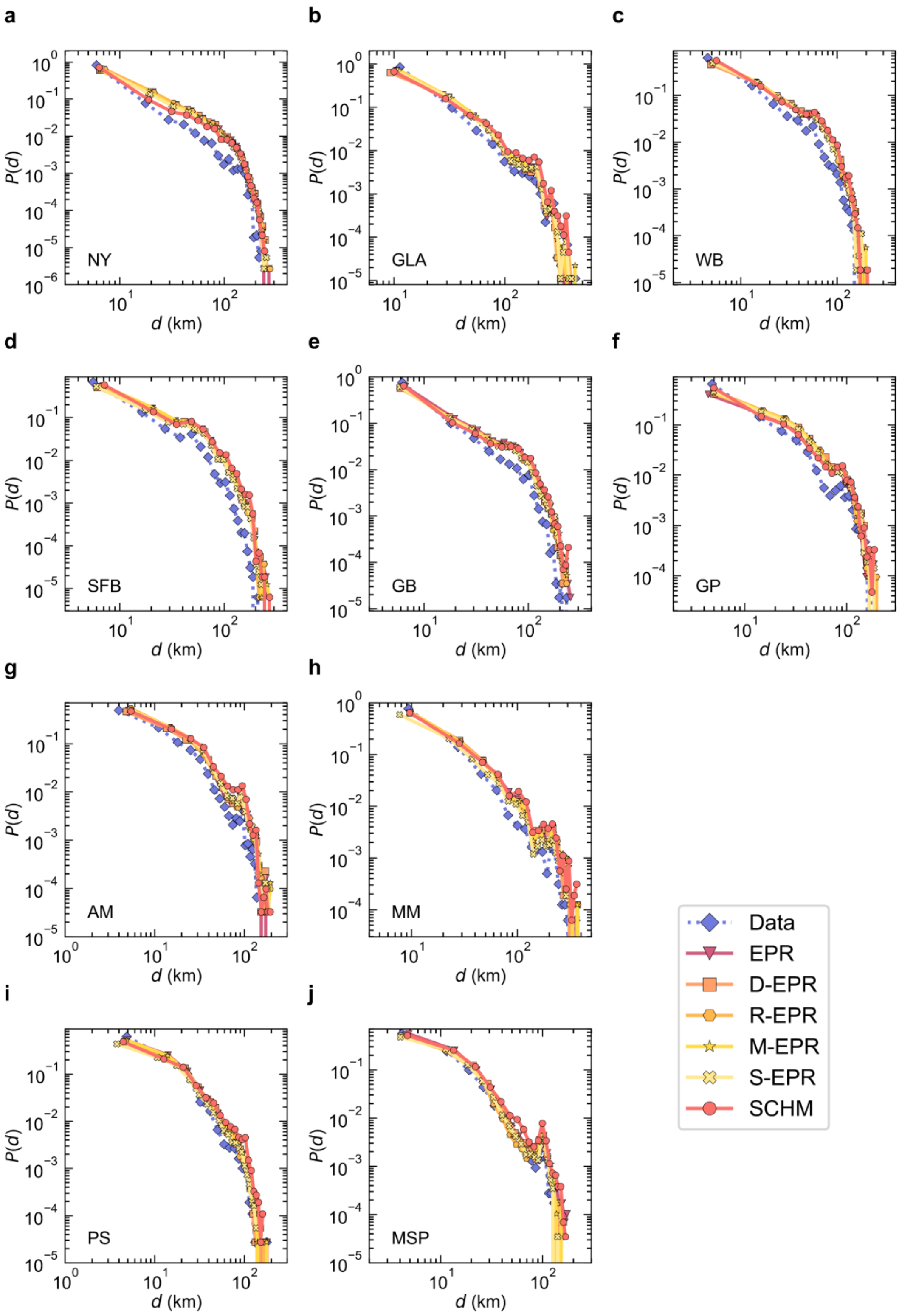

Figure S30. Comparison of travel distance distributions for 10 large cities.

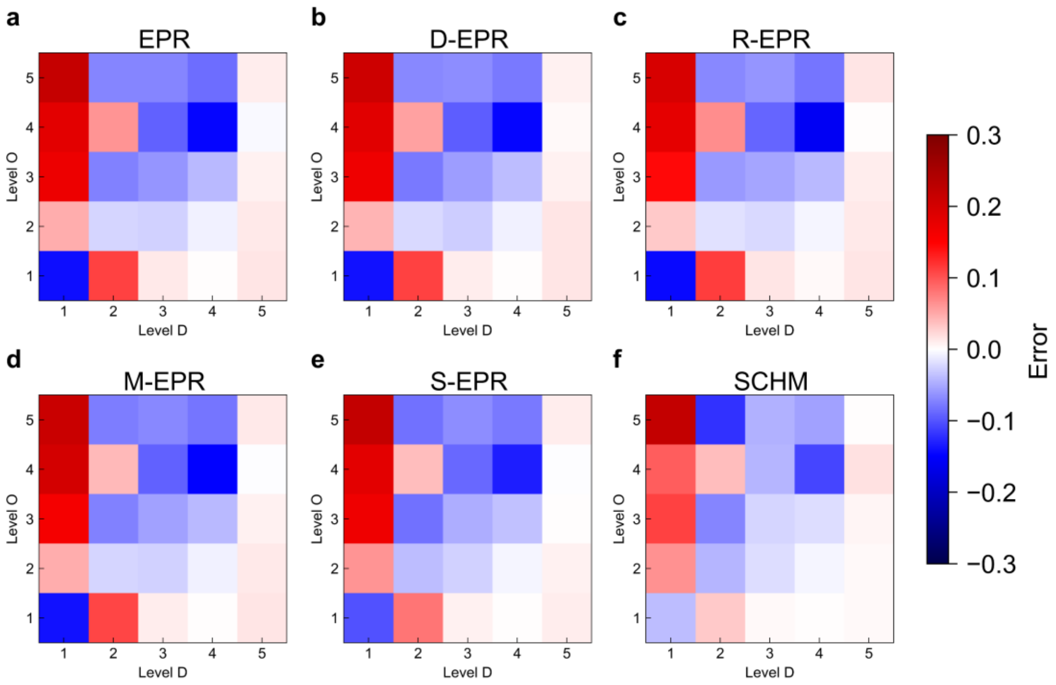

Figure S31. Visitation frequency error between locations with different segregation levels in the New York-Newark CSA. The color of the matrix is red (blue), indicating that the predicted value is greater (less) than the true value.

Supplemental tables

Table S1. Data statistics of the combined statistical area

Combined statistical area	Abb.	County number	Tract number	Population
New York–Newark	NY	31	5,310	22,431,833
Los Angeles–Long Beach	GLA	6	4,015	18,644,680
Washington–Baltimore–Arlington	WB	39	2,168	10,028,331
San Jose–San Francisco–Oakland	SFB	14	1,934	9,225,160
Boston–Worcester–Providence	GB	19	1,768	8,349,768
Philadelphia–Reading–Camden	GP	15	1,705	7,379,700
Atlanta–Athens-Clarke County–Sandy Springs	AM	41	1,105	6,976,171
Miami–Port St. Lucie–Fort Lauderdale	MM	7	1,362	6,908,296
Seattle–Tacoma	PS	8	892	4,953,421
Minneapolis–St. Paul	MSP	21	861	4,078,788

Note: "Abb." stands for abbreviation.

Table S2. Representativeness of segregation values calculated based on income quantiles of the sample and population

CSA name	NY	GLA	WB	SFB	GB	GP	AM	MM	PS	MSP
Coverage	65.0%	59.0%	79.0%	73.0%	63.7%	51.7%	62.2%	62.7%	65.8%	72.1%
PCC	0.58	0.44	0.76	0.75	0.55	0.52	0.48	0.52	0.60	0.70
p-value	0.00	0.00	0.00	0.00	0.00	0.00	0.00	0.00	0.00	0.00

Note: "PCC" denotes the Pearson correlation coefficient.

Table S3. Pearson correlation coefficients between segregation values calculated using quartiles and those obtained using other quantile interval settings (i.e., tertiles and quintiles)

CSA name	NY	GLA	WB	SFB	GB	GP	AM	MM	PS	MSP
Quartiles - tertiles	0.79	0.79	0.78	0.79	0.78	0.77	0.75	0.74	0.79	0.76
p-value	0.00	0.00	0.00	0.00	0.00	0.00	0.00	0.00	0.00	0.00
Quartiles-quintiles	0.85	0.86	0.85	0. 86	0.85	0.85	0.83	0.85	0.86	0.85
p-value	0.00	0.00	0.00	0.00	0.00	0.00	0.00	0.00	0.00	0.00

Table S4. Pearson correlation coefficients between segregation values calculated based on the segregation metric used in this study and those obtained from alternative segregation metrics

CSA name	NY	GLA	WB	SFB	GB	GP	AM	MM	PS	MSP
Euclidean distance	0.955	0.953	0.953	0.952	0.951	0.954	0.952	0.953	0.951	0.953
p-value	0.00	0.00	0.00	0.00	0.00	0.00	0.00	0.00	0.00	0.00
Entropy	-0.979	-0.979	-0.979	-0.979	-0.979	-0.979	-0.979	-0.980	-0.979	-0.980
p-value	0.00	0.00	0.00	0.00	0.00	0.00	0.00	0.00	0.00	0.00

Table S5. Confidence intervals of the segregation visitation index estimated using segregation values derived from different segregation metrics

CSA name	Euclidean distance-based metric	Entropy-based metric	Our used metric
NY	[-6.14, -5.21]	[-7.33, -6.29]	[-7.22, -5.93]
GLA	[-6.35, -5.15]	[-7.94, -6.79]	[-5.35, -4.49]
WB	[-5.41, -4.78]	[-7.70, -6.78]	[-7.67, -6.56]
SFB	[-6.28, -5.37]	[-8.22, -7.12]	[-8.33, -7.22]
GB	[-5.02, -4.18]	[-6.75, -5.55]	[-6.79, -5.73]
GP	[-4.79, -3.31]	[-6.69, -5.20]	[-6.70, -5.02]
AM	[-3.50, -1.18]	[-7.38, -5.99]	[-7.27, -5.71]
MM	[-3.39, -2.50]	[-7.06, -6.05]	[-6.96, -5.98]
PS	[-2.74, -2.14]	[-7.71, -6.21]	[-7.52, -6.10]
MSP	[-4.18, -3.41]	[-6.18, -5.00]	[-6.37, -5.08]

Table S6. Confidence intervals of the segregation visitation index obtained from uncertainty estimation using different index functions

CSA name	Proposed index		SPMI	
	Confidence interval	Mean	Confidence interval	Mean
NY	[-6.14, -5.20]	-5.65	[-0.86, -0.81]	-0.84
GLA	[-6.35, -5.15]	-5.71	[-0.79, -0.74]	-0.77
WB	[-5.41, -4.78]	-5.11	[-0.66, -0.61]	-0.64
SFB	[-6.28, -5.37]	-5.82	[-0.84, -0.81]	-0.83
GB	[-5.02, -4.18]	-4.60	[-0.73, -0.68]	-0.70
GP	[-4.79, -3.31]	-4.04	[-0.62, -0.51]	-0.57
AM	[-3.50, -1.18]	-2.31	[-0.49, -0.25]	-0.38
MM	[-3.39, -2.50]	-2.92	[-0.45, -0.34]	-0.40
PS	[-2.74, -2.14]	-2.44	[-0.40, -0.31]	-0.36
MSP	[-4.18, -3.41]	-3.77	[-0.74, -0.67]	-0.71

Table S7. Spearman correlation of segregation values between travel flow and travel degree for 10 large cities

Mobility measure	Metric	NY	GLA	WB	SFB	GB	GP	AM	MM	PS	MSP
Flow	SPC	-0.31	-0.36	-0.26	-0.40	-0.31	-0.20	-0.27	-0.26	-0.31	-0.33
	p-value	0.00	0.00	0.00	0.00	0.00	0.00	0.00	0.00	0.00	0.00
Degree	SPC	-0.39	-0.43	-0.36	-0.49	-0.39	-0.26	-0.40	-0.31	-0.37	-0.36
	p-value	0.00	0.00	0.00	0.00	0.00	0.00	0.00	0.00	0.00	0.00
Income	PCC	0.35	0.20	0.20	0.16	0.16	0.10	0.31	0.08	0.18	0.10
	p-value	0.00	0.00	0.00	0.00	0.00	0.00	0.00	0.00	0.00	0.00

Note: "SPC" denotes the Spearman correlation coefficient. "PCC" denotes the Pearson correlation coefficient.

Table S8. Performance comparison of different models for 10 large cities

CSA	Metric	EPR	D-EPR	M-EPR	R-EPR	S-EPR	SCHM
NY	CPC	0.499	0.500	0.508	0.494	0.563	**0.636**
	RMSE	6.550	6.550	6.450	6.224	5.836	**4.688**
	MAE	2.376	2.379	2.351	2.431	2.217	**2.055**
GLA	CPC	0.378	0.373	0.391	0.383	0.422	**0.469**
	RMSE	3.068	3.051	2.916	3.016	2.824	**2.588**
	MAE	1.551	1.556	1.537	1.578	1.510	**1.494**
WB	CPC	0.437	0.435	0.457	0.436	0.486	**0.558**
	RMSE	4.967	4.962	4.689	4.745	4.512	**3.613**
	MAE	1.891	1.888	1.846	1.922	1.812	**1.720**
SFB	CPC	0.528	0.526	0.538	0.528	0.579	**0.640**
	RMSE	9.096	9.099	8.598	8.112	7.763	**5.546**
	MAE	2.296	2.298	2.265	2.338	2.152	**1.980**
GB	CPC	0.461	0.457	0.477	0.469	0.509	**0.567**
	RMSE	5.237	5.258	4.969	4.977	4.811	**3.935**
	MAE	1.957	1.966	1.920	1.973	1.870	**1.816**
GP	CPC	0.377	0.383	0.396	0.380	0.438	**0.513**
	RMSE	4.010	3.905	3.947	3.936	3.689	**2.928**
	MAE	1.738	1.723	1.711	1.771	1.660	**1.598**
AM	CPC	0.472	0.469	0.476	0.469	0.504	**0.528**
	RMSE	3.587	3.628	3.590	3.564	3.445	**3.270**
	MAE	1.759	1.774	1.759	1.808	1.726	**1.739**
MM	CPC	0.352	0.350	0.386	0.373	0.405	**0.636**
	RMSE	2.869	2.876	2.771	2.633	2.719	**4.688**
	MAE	1.552	1.550	1.506	1.538	1.492	**2.055**
PS	CPC	0.535	0.536	0.554	0.546	0.578	**0.633**
	RMSE	6.384	6.450	6.198	5.971	5.729	**4.645**
	MAE	2.314	2.314	2.267	2.306	2.197	**2.058**
MSP	CPC	0.470	0.469	0.488	0.484	0.517	**0.564**
	RMSE	5.581	5.739	5.325	5.072	4.964	**3.753**
	MAE	1.797	1.800	1.770	1.820	1.712	**1.645**

Note: Bold font represents the optimal value of each metric.

Table S9. Prediction error of cross-segregation level visitation probability

CSA	Metric	EPR	D-EPR	M-EPR	R-EPR	S-EPR	SCHM
NY	RMSE	0.091	0.089	0.091	0.087	0.071	**0.069**
	MAE	0.068	0.066	0.067	0.064	0.054	**0.048**
GLA	RMSE	0.051	0.057	0.050	0.049	0.051	**0.037**
	MAE	0.038	0.043	0.038	0.037	0.035	**0.025**
WB	RMSE	0.043	0.051	0.046	0.058	0.044	**0.027**
	MAE	0.031	0.037	0.034	0.043	0.033	**0.016**
SFB	RMSE	0.070	0.066	0.069	**0.063**	0.063	0.064
	MAE	0.046	0.045	0.046	0.042	0.044	**0.037**
GB	RMSE	0.084	0.081	0.078	0.073	0.064	**0.065**
	MAE	0.059	0.056	0.054	0.049	0.044	**0.042**
GP	RMSE	0.058	0.060	0.053	0.047	0.046	**0.025**
	MAE	0.043	0.045	0.039	0.033	0.037	**0.019**
AM	RMSE	0.052	0.047	0.048	0.048	0.057	**0.036**
	MAE	0.039	0.035	0.035	0.036	0.037	**0.022**
MM	RMSE	0.045	0.045	0.036	0.044	0.041	**0.030**
	MAE	0.034	0.033	0.027	0.032	0.031	**0.022**
PS	RMSE	0.047	0.048	0.046	0.051	0.058	**0.045**
	MAE	0.031	0.034	0.029	0.037	0.041	**0.028**
MSP	RMSE	0.066	0.077	0.071	0.071	0.059	**0.037**
	MAE	0.047	0.052	0.046	0.050	0.037	**0.025**

Supplemental notes

Note S1. Calculation of income segregation

The most commonly used method for calculating income segregation based on human mobility data is to assess whether a target location is uniformly visited by various income groups. Following previous empirical studies[1,2], the income segregation experienced by various locations within large cities can be calculated by

$$S_i = \frac{Q}{2Q-2}\sum_{q=1}^{Q} |\tau_{qi} - \frac{1}{Q}| \tag{1.1}$$

where S_i denotes the income segregation value experienced by location i. Q denotes the number of income quantiles, which is set to four in this study. τ_{qi} denotes the proportion of people with income quantile q who visit location i. If the visit ratio of different groups is $1/Q$, the segregation value of location i is zero, that is, it does not suffer from income segregation.

Note S2. Income representativeness used for segregation calculation

In this study, we determine the income segregation value of a location by evaluating the proportion of each income group that visits it. Meanwhile, we use the average income distribution of individuals active within the target city to establish quantiles for categorizing income levels. To ensure the robustness of our segregation values against the quantile definition, we conducted a correlation analysis comparing segregation values derived from the income quantile of the sample versus those of the overall population. Specifically, the sample refers to the average income distribution across all census tracts covered by active individuals in the target city. The population refers to the average income distribution across all census tracts in the target city. If the quantiles calculated from both distributions are similar, it indicates that the income distribution of the sample areas closely resembles that of the population, suggesting representativeness. Table S2 shows that our data achieve at least 60% coverage, where income levels correspond to the same bin as those based on the population data. The correlation between segregation values based on samples and population is strong, with almost all p-values approaching zero. It is important to note that the segregation value calculated based on the population uses the quantiles derived from the population's income distribution to categorize individual's income level, which is then used to calculate the segregation value. Given that our data sample represents only a fraction of the population, we further evaluated the variability of the income distribution and its impacts on the model results. We follow the pipeline of previous work[3] and use the bootstrapping method for representative statistical analysis of the sample's income distribution. We performed 1,000 iterations for each city's sample, generating 1,000 resampled datasets. As illustrated in Figure S4, the mean user income of these resampled sets closely aligns with the target population, with a 95% confidence interval indicating that the income level error is within 0.2 units. This confirms that our sample does not significantly deviate from the overall population[3].

Note S3. Sensitivity analysis for income segregation calculation

To assess the robustness of the income segregation calculation, we conducted sensitivity analyses with respect to both quantile interval settings and the choice of segregation metrics. First, we altered the

number of income quantile intervals (three, four, and five) and examined the correlation of segregation values across all locations (see Table S3). The results show that our segregation metric is largely insensitive to the number of quantile intervals: across cities, the segregation distributions remain highly correlated ($\mathrm{PCC} > 0.75,\ p \ll 0.05$). Second, with the quantile interval fixed at four, we compared segregation values derived from our metric with those calculated using two alternative measures—Euclidean distance–based and entropy-based segregation metrics, as shown in Table S4. We can also see that the segregation values obtained under different metrics exhibit strong correlations. The correlation between the segregation values calculated using our own metric, and the segregation values calculated using the Euclidean distance metric ($S_i^{euclidean} = \sum_{q=1}^{Q}\left(\tau_{qi} - \frac{1}{4}\right)^2$) and the segregation values calculated using entropy metric[4] ($S_i^{entropy} = \frac{1}{\log 4}\sum_{q=1}^{Q}\tau_{qi}\log(\tau_{qi})$) are greater than 0.95 ($p \ll 0.05$). Nevertheless, we still used our metric because it offers greater variability over the range of [0, 1], enabling finer discrimination of segregation levels across locations.

Note S4. Robustness analysis for segregation visitation index calculation

We further examined the robustness of the proposed segregation visitation index with respect to alternative segregation metrics. Specifically, we computed segregation values at each location using the three metrics described above, randomly sampled trips from each location, and constructed segregation-constrained visitation matrices to derive corresponding visitation indices. The confidence intervals of the resulting indices (see Table S5) show that our metric yields a more stable distribution, reflected by narrower confidence intervals than those from the Euclidean-based, and entropy-based metrics. Further, with the same number of quantile intervals and segregation metric settings, we introduced the socioeconomic preferential mobility index[5] (SPMI) as an alternative segregation index to test the reliability of the proposed segregation visitation index. Following the same sampling procedure, we calculate SPMI as follows:

$$SPMI = \frac{S_{lower} - S_{upper}}{S_{lower} + S_{upper}} \tag{1.2}$$

where S_{lower} (S_{upper}) denotes the total probability of trips from higher- (lower-) segregation origins to lower- (higher-) segregation destinations. An SPMI of 0 indicates exclusive within-level visits, while 1 (−1) reflects a complete preference for lower (higher) segregation destinations. As can be observed from Table S6, SPMI yields narrower confidence intervals because we considered the degree of across segregation levels in the proposed index, thus resulting in an index with a wider numerical range. We further performed correlation analyses between segregation indices (from both methods) and a range of urban and environmental variables (see Figures S19-S21). Both indices show comparable correlation strengths across variables, supporting the robustness of our findings. Notably, land-use entropy displays only weak correlation with the segregation visitation index, and average regional income levels are not strongly correlated with total segregation visitation intensity. These results suggest that income segregation patterns are shaped by more complex, multidimensional factors that extend beyond income alone.

Note S5. Metrics for mobility pattern analysis

We explore the variations in mobility patterns of groups at locations with different segregation levels, considering travel degree D, average travel distance d, travel entropy E, and travel clustering coefficient Cc (see Figures S10-S13). Specifically, travel degree indicates the number of different locations visited from a given location, while average travel distance represents the range of movement from that location. Travel entropy[6] represents the diffusion degree of travel flow starting from a location and is defined as follows:

$$E_i = -\sum_{j=1}^{N} p_{ij} \log_2 p_{ij} \tag{1.3}$$

where p_{ij} denotes the travel probability from location i to location j. N denotes the total number of locations visited from location i. A higher travel entropy indicates that people travel to a more diverse set of locations. Similarly, the travel clustering coefficient[7] describes the cliqueness or transitivity of people's travel network by measuring the presence of triangles in the travel network starting from location i. It is defined as follows:

$$Cc_i = \frac{1}{D_i(D_i - 1)} \sum_{j=1}^{N} \sum_{k=1}^{M} (p_{ij} p_{jk} p_{ki})^{1/3} \tag{1.4}$$

where D_i denotes the degree of node i. N and M denote the total number of locations visited from location i and location j, respectively. p_{ij}, p_{jk}, and p_{ki} represent the travel probabilities between the corresponding locations.

Note S6. SHapley Additive exPlanations (SHAP) analysis

We construct a segregation level classification model utilizing the XGBoost algorithm to investigate the correlations between different input variables and segregation levels. To thoroughly analyze each variable's impact on the segregation value across different segregation levels, and its contribution at each level, we employ the SHAP method[8] for visual analysis.

The SHAP method originates from the Shapley value concept grounded in cooperative game theory. SHAP assigns a "Shapley value" to each feature value by calculating its contribution to the predicted output across all possible permutations of the feature value. This value signifies the contribution of each feature to the final prediction, offering an intuitive and comprehensive way of interpreting the model output. Illustrated in Figure S14, we scrutinize the relationship between nine variables and five segregation levels. The color of the scatter points denotes the magnitude of the variable. If the scatter points appear on the right side of the x-axis, it implies a positive impact on the model output, and vice versa. The variables on the y-axis are arranged in descending order of importance. As shown in Figure S14, the direction and intensity of influence of each variable differ under different levels. Higher segregation levels are linked with larger clustering coefficients and smaller travel entropy, while lower segregation levels are associated with greater travel entropy and a more diverse range of POIs. These results affirm the heterogeneity of human mobility patterns under varying segregation levels and underscore the complexity of factors influencing income segregation.

Note S7. Reference models

To validate that the segregation-constrained human flow patterns described result from the biased

visitation behaviors of groups with different segregation levels, we introduce three reference models for comparative analysis. We obtain the total number of trips at each location based on observation data, and then maintain the total constant to allocate travel flows based on different rules, including null-based, gravity-based, and radiation-based. The null-based reference model indicates that different locations have an identical visitation probability. Gravity-based and radiation-based reference models utilize gravity and radiation models to define the visitation probability between locations, respectively. The gravity model and radiation model are defined as follows:

$$p_{ij}^{GM} = \frac{Pop_j^{\gamma_1}}{d_{ij}^{\gamma_2}} \tag{1.5}$$

$$p_{ij}^{RM} = \frac{Pop_i Pop_j}{(Pop_i + s_{ij})(Pop_i + s_{ij} + Pop_j)} \tag{1.6}$$

where $d_{ij}^{\gamma_2}$ represents the distance between location i and location j. Parameters γ_1 and γ_2 are to be estimated. Pop_i and Pop_j denote the population of location i and location j, respectively, while s_{ij} denotes the number of intervention opportunities between location i and location j. Consequently, we derive three travel networks based on the three reference models. Further, we obtain the corresponding segregation-constrained visitation matrix, respectively, and calculate the corresponding SVI value (see Figure S19).

Note S8. Predictability analysis of human mobility at different segregation levels

Recognizing the variations in collective mobility patterns in locations across different segregation levels, we construct gravity models based on different training strategies to further explore the predictability of mobility patterns at these levels. Initially, we utilize travel data from all levels within a large city to construct an origin-constrained gravity model[9], simultaneously estimating human flows between all locations, denoted as Global GM. On the other hand, we construct dedicated gravity models based on the travel data of each segregation level to estimate human flows at the single segregation level, denoted as Local GM. Meanwhile, for each model, we divided the travel data in a ratio of 8:2 for model training and testing. In the main text (**Figure 6c**), we compare the predictive performance of these two models. Due to the heterogeneous mobility patterns among various segregation levels, local GM exhibits superior estimation performance in comparison.

Note S9. Baseline models

To assess the trajectory generation performance of the segregation-constrained human mobility model (SCHM), we compare SCHM's performance with the exploration and preference return[10] (EPR) model and its four variant models, namely, gravity-based EPR[11] (D-EPR), recency-based EPR[12] (R-EPR), memory-based EPR[13] (M-EPR), and social EPR[1] (S-EPR).

In each step of selecting a location for travel, the EPR model opts to return to the previously visited location with a probability of $1 - \rho S^{-\gamma}$, and explore a new location with a probability of $\rho S^{-\gamma}$. In this process, the selection of the returned location is contingent on the visitation frequency. Parameters ρ and γ drawn from empirical research[10,14]. Concerning the variant models, the D-EPR model extends the EPR's exploration process, and enhances individual exploration accuracy by incorporating a gravity model. The R-EPR model extends the return stage of the EPR model, and improves individual return

selection accuracy based on frequency ranking $Rank_f(loc_i)^{-1-\gamma}$ and recency ranking $Rank_r(loc_i)^{-\eta}$ of location loc_i. Wherein parameters α, γ, and η are determined to be 0.6, 0.21, and 0.8 based on empirical research[12]. Similarly, the M-ERP model also enhances the return stage of the EPR model by constraining only the memory impact of the individual's recent M days. Moreover, the S-EPR model introduces individual income segregation visitation constraints by optimizing the exploration phase of the EPR model. That is, individuals decide with probability σ_s whether to visit a location where the income level of the vast majority of people is not comparable to their own, or with probability $1-\sigma_s$ to visit a location where the income level of the vast majority of people is comparable to their own. Meanwhile, σ_s denotes the proportion of places visited by the user where their income group is the minority.

Note S10. Performance evaluation

Three widely used metrics are used to measure the model performance, including the common part of commuters[9] (CPC), mean absolute error (MAE), and root mean squared error (RMSE):

$$\mathrm{CPC} = \sum_{i}^{N}\sum_{j\neq i}^{N}\frac{2\times\min\{T_{ij},T'_{ij}\}}{\sum_{i,j}T_{ij}+\sum_{i,j}T'_{ij}} \tag{2.1}$$

$$\mathrm{MAE} = \frac{1}{N(N-1)}\sum_{i}^{N}\sum_{j\neq i}^{N}|T_{ij}-T'_{ij}| \tag{2.2}$$

$$\mathrm{RMSE} = \sqrt{\frac{1}{N(N-1)}\sum_{i}^{N}\sum_{j\neq i}^{N}\left(T_{ij}-T'_{ij}\right)^2} \tag{2.3}$$

where T_{ij} and T'_{ij} denote the actual and predicted trips from location i to location j, respectively. N denotes the total number of locations.

Supplemental references

1. Moro, E., Calacci, D., Dong, X., and Pentland, A. (2021). Mobility patterns are associated with experienced income segregation in large US cities. Nat. Commun. *12*, 4633. https://doi.org/10.1038/s41467-021-24899-8.
2. Sun, C., Shibuya, Y., and Sekimoto, Y. (2024). Social segregation levels vary depending on activity space types: Comparison of segregation in residential, workplace, routine and non-routine activities in Tokyo metropolitan area. Cities *146*, 104745. https://doi.org/10.1016/j.cities.2023.104745.
3. Hilman, R. M., Iñiguez, G., and Karsai, M. (2022). Socioeconomic biases in urban mixing patterns of US metropolitan areas. EPJ Data Sci. *11*, 32. https://doi.org/10.1140/epjds/s13688-022-00341-x.
4. Massey, D. S., and Denton, N. A. (1988). The dimensions of residential segregation. Soc. forces *67*, 281–315. https://doi.org/10.1093/sf/67.2.281.
5. Liao, Y., Gil, J., Yeh, S., Pereira, R. H., and Alessandretti, L. (2025). Socio-spatial segregation and human mobility: A review of empirical evidence. Comput. Environ. Urban Syst. *117*, 102250. https://doi.org/10.1016/j.compenvurbsys.2025.102250.
6. Mowshowitz, A. (1968). Entropy and the complexity of graphs: I. An index of the relative complexity of a graph. Bull. Math Biophys. *30*, 175–204. https://doi.org/10.1007/BF02476948.
7. Onnela, J. P., Saramäki, J., Kertész, J., and Kaski, K. (2005). Intensity and coherence of motifs in weighted complex networks. Phys. Rev. E *71*, 065103. https://doi.org/10.1103/PhysRevE.71.065103.
8. Lundberg, S. M., and Lee, S. I. (2017) A unified approach to interpreting model predictions. In Proc. 31st Conf. on Neural Inf. Process. Syst. pp. 4768–4777.
9. Barbosa, H., Barthelemy, M., Ghoshal, G., James C., Lenormand, M., Louail, T., Menezes, R., Ramasco, J., Simini, F., and Tomasini, M. (2018). Human mobility: Models and applications. Phys. Rep. *734*, 1–74. https://doi.org/10.1016/j.physrep.2018.01.001.
10. Song, C., Koren, T., Wang, P., and Barabási, A.L. (2010). Modelling the scaling properties of human mobility. Nat. Phys. *6*, 818–823. https://doi.org/10.1038/nphys1760.
11. Pappalardo, L., Simini, F., Rinzivillo, S., Pedreschi, D., Giannotti, F., and Barabási, A.L. (2015). Returners and explorers dichotomy in human mobility. Nat. Commun. *6*, 8166. https://doi.org/10.1038/ncomms9166.
12. Barbosa, H., de Lima-Neto, F.B., Evsukoff, A., and Menezes, R. (2015). The effect of recency to human mobility. EPJ Data Sci. *4*, 21. 10.1140/epjds/s13688-015-0059-8.
13. Alessandretti, L., Sapiezynski, P., Sekara, V., Lehmann, S., and Baronchelli, A. (2018). Evidence for a conserved quantity in human mobility. Nat. Hum. Behav. *2*, 485–491. https://doi.org/10.1038/s41562-018-0364-x.
14. Song, C., Koren, T., Wang, P., and Barabási, A.L. (2010). Modelling the scaling properties of human mobility. Nat. Phys. *6*, 818–823. https://doi.org/10.1038/nphys1760.
